# Not‐So‐Innocent Anions Determine the Mechanism of Cationic Alkylators

**DOI:** 10.1002/chem.202004208

**Published:** 2021-01-18

**Authors:** S. Maryamdokht Taimoory, Vincenzo Alessandro Cataldo, Andreas Schäfer, John F. Trant, Ryan Guterman

**Affiliations:** ^1^ Department of Chemistry and Biochemistry University of Windsor 401 Sunset Ave. ON N9B 3P4 Windsor Canada; ^2^ Department of Chemistry University of Michigan 930 N University Ave. Ann Arbor MI 48109 USA; ^3^ Colloid Chemistry Department Max Planck Institute for Colloids and Interfaces Am Mühlenberg 1 OT Golm 14476 Potsdam Germany; ^4^ Institut für Chemie und Biochemie–Organische Chemie Freie Universität Berlin Takustr. 3 14195 Berlin Germany

**Keywords:** alkylation, colloids, computational chemistry, ionic liquids, reaction mechanisms

## Abstract

Alkylating reagents based on thioimidazolium ionic liquids were synthesized and the influence of the anion on the alkylation reaction mechanism explored in detail using both experimental and computational methods. Thioimidazolium cations transfer alkyl substituents to nucleophiles, however the reaction rate was highly dependent on anion identity, demonstrating that the anion is not innocent in the mechanism. Detailed analysis of the computationally‐derived potential energy surfaces associated with possible mechanisms indicated that this dependence arises from a combination of anion induced electronic, steric and coordinating effects, with highly nucleophilic anions catalyzing a 2‐step process while highly non‐nucleophilic, delocalized anions favor a 1‐step reaction. This work also confirms the presence of ion‐pairs and aggregates in solution thus supporting anion‐induced control over the reaction rate and mechanism. These findings provide new insight into an old reaction allowing for better design of cationic alkylators in synthesis, gene expression, polymer science, and protein chemistry applications.

## Graphical abstract

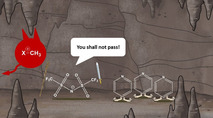

## Introduction

In nucleophilic substitution, an electron‐rich nucleophile attacks an electron‐poor electrophile, replacing a leaving group. Despite being an elementary reaction, challenges remain in predicting seemingly simple reaction outcomes whether in biochemical pathways,^[1]^ the design of cancer therapeutics,[^2^, ^3^] the preparation of challenging natural products and pharmaceuticals,[^4^, ^5^] the development of next‐generation alkylation technologies,[^6^, ^7^] or in understanding the mechanism of endogenous alkylators. For example, S‐Adenosyl methionine (SAM)[^8^, ^9^, ^10^] is a cofactor that regulates a series of biochemical transformations via transfer of the methyl group located on a positively charged sulfur atom (Scheme S1). Numerous studies have focused on understanding SAM's alkylation cycle and interaction with enzymes containing a negatively charged pocket;[^11^, ^12^, ^13^] however, no in‐depth studies have sought to understand the eventual role of counteranions on the function of SAM. This begs the question whether free anions or anionic residues in the active site have an active role on the alkylation reaction by lowering activation energies or changing reaction pathways.

Answering these questions is particularly important to drive progress in alkylation technology and the synthesis and understanding of custom reagents for niche applications. For example, solid‐supported alkylators are finding applications in flow‐chemistry since they eliminate the need for side product removal, simplify purification, and reimagine the role of alkylation reaction in complex reactor setups.[^14^, ^15^] Other “smart” alkylation technologies rely on the use of triggers such as light,[^16^, ^17^] certain enzymes,[^18^, ^19^] and electrophiles,^[20]^ which provides both temporal and spatial control over the alkylation process. Semi‐stable cations capable of controlled transalkylation have led to the discovery of an entirely new class of polymer vitrimers pioneered by Drockenmuller et al.^[21]^ and demonstrates a non‐conventional use for cationic alkylators beyond synthesis.[^22^, ^23^, ^24^]

**Scheme 1:**
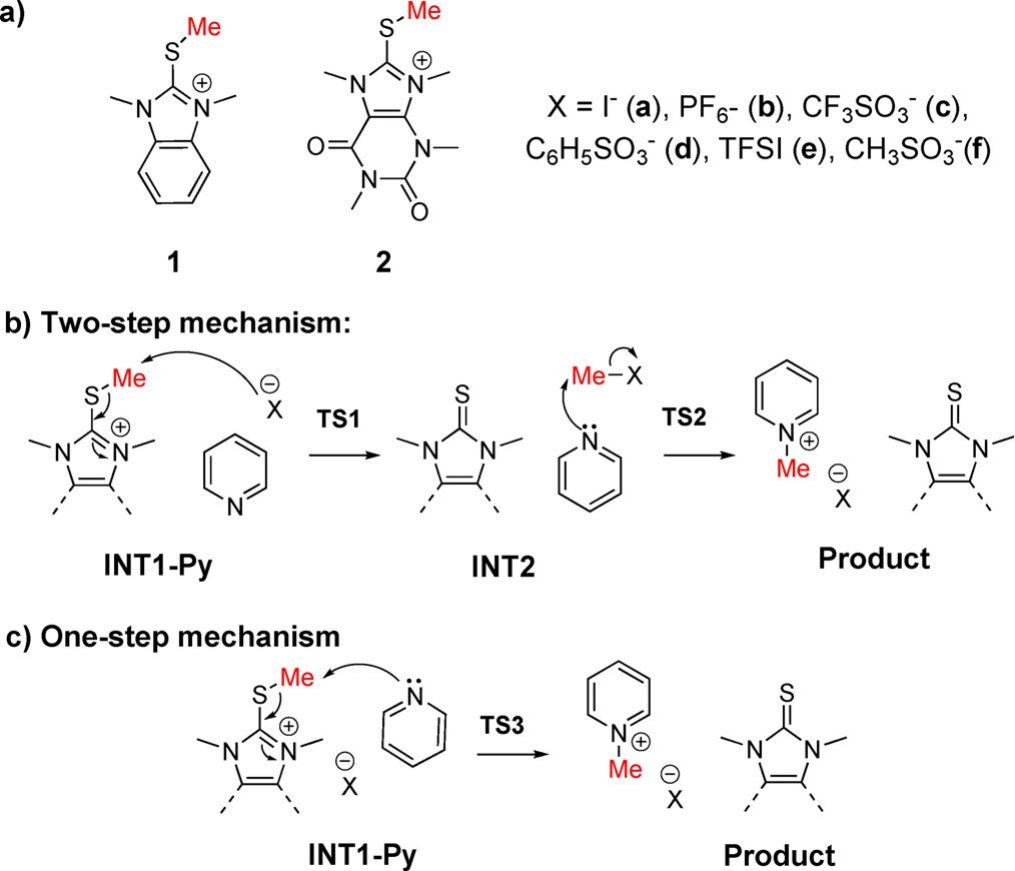
Chemical structures of the thiouronium salts prepared and the proposed mechanisms for their counteranion mediated alkylation reaction with pyridine.

We have been developing a new class of highly tunable and non‐volatile cationic alkylating agents based on thioimidazolium ionic liquids.[^25^, ^26^] In contrast with oxonium, ammonium, or sulfonium analogues, thioimidazoliums are more easily derivatizable and often milder alkylating reagents. For example, alkyl groups attached to the sulfur atom are exclusively transferred to a nucleophile under mild reaction conditions leaving all other positions unaffected, thus allowing for their derivatization without compromising product formation. Our preliminary investigations showed that electron deficient thioimidazolium cations have weaker S‐R bonds and correspondingly faster alkylation reactions^[25]^ and that exchanging the iodide counter‐anion with the much less nucleophilic bis(trifluoro‐methane)‐sulfonimide (TFSI) decreased the rate constant by 100‐fold for reactions with pyridine. The addition of KI (0.1–1.0 equiv) to this reaction mixture increased the reaction rate,^[25]^ thus demonstrating anion‐dependent reactivity. We postulated that iodide catalyzes alkylation via a transient alkyl iodide intermediate (Scheme 1 b), confirmed by the formation of MeI in solution upon heating,^[25]^ while the non‐nucleophilic TFSI anion likely forced a one‐step process (Scheme 1 c) that for an unknown reason, proceeded slower. Despite these observations, the existence of a two‐step mechanism for iodide does not preclude a concurrent one‐step mechanism mediated by an iodide‐cation complex, which may in fact be the lower energy pathway. These initial findings suggested mechanistic complexity for a seemingly simple reaction with implications for alkylation in material science, synthesis, and living systems. An understanding of the role of ion‐pairing dynamics, aggregation, and structure–activity relationships is required for effective synthetic reagents to be developed. This is a very understudied field, with only a few examples explicitly exploring the mechanistic role of counterions in complex reactions,^[27]^ especially when changing the ion changes the product outcome; but none focusing on the comparably subtle effects observed here. The former are highly exciting, but the latter class, where changing the counterion simply accelerates or retards a reaction are far more common, and very industrially and biologically relevant. We need a better understanding of these more routine, but far more common phenomena.

In this context, we here explore the role of the anion in cationic alkylations through a combination of experimental and computational analysis. Two series of thioimidazolium salts, based on benzimidazolium and caffeine scaffolds with six different anions were prepared as models to examine anion‐influenced reactivity. By coupling kinetic analysis, molar conductivity, and ion‐diffusivity data with molecular mechanics (MM), conformational analysis, and advanced quantum mechanical (QM) DFT‐studies, we obtained a comprehensive picture of how and why anions influence the reaction kinetics and mechanisms of these cationic alkylators. This combined‐arms investigation demonstrates how “spectator” anions determine reactivity and brings new life to seemingly simple S_N_2 reactions.

## Results and Discussion

### Thioimidazolium synthesis and kinetic evaluation

Thioimidazolium salts were synthesized according to a modified version of our published procedure (See Supporting Information and schemes S2 and S3).^[25]^ To assess the anion's effect on alkylation kinetics, all salts were treated with 1.0 equiv of pyridine in DMSO at 90 °C and conversion to 1‐methylpyridinium was monitored by ^1^H‐NMR spectroscopy over 15 h. Second‐order rate constants were obtained by plotting 1/[Pyr] as a function of time and determining the slope (Table 1). Caffeine‐based salts were more reactive than their benzimidazole analogues, consistent with previous findings.^[25]^ The relative reaction rates of the salts as a function of anion was the same in both series: I^−^≫PF_6_^−^>CF_3_SO_3_^−^>PhSO_3_^−^>TFSI>CH_3_SO_3_^−^, indicating that the effect of the anion on the reaction rate is not cation‐dependent for the series of tested cations. It was unclear what differentiates the other anions While we suspected that a transient MeI intermediate is responsible for the exceptionally high reactivity of the iodides, their reaction order is not consistent with the expected nucleophilicities of these anions based on their nucleofugality: I≫PhSO_3_^−^≈CH_3_SO_3_^−^>TFSI>CF_3_SO_3_^−^≫PF_6_^−^ (experimental Mayr values have not been computed for these anions).^[28]^ PF_6_^−^ cannot form another covalent bond, rendering it incapable of “shuttling” the methyl substituent, yet it is the second most reactive salt, trailed closely by triflate, a very weak nucleophile.

**Table 1:** Rate constants k (M^−1^ min^−1^) for the alkylation of pyridine by a given alkylator (462 mm) at a ratio of 1:1 in DMSO.^[a]^

Anion	Cation 1	Cation 2
iodide (a)	7.5×10^−1^±8.5×10^−2^	9.6×10^−1^±6.2×10^−2^
PF_6_^−^ (b)	8.4×10^−3^±0.6×10^−4^	9.3×10^−3^±2.1×10^−4^
CF_3_SO_3_^−^ (c)	7.7×10^−3^±1.8×10^−4^	8.8×10^−3^±1.0×10^−4^
C_6_H_5_SO_3_^−^ (d)	4.2×10^−3^±0.7×10^−4^	4.7×10^−3^±1.4×10^−4^
TFSI (e)	4.1×10^−3^±0.6×10^−4^	6.9×10^−3^±0.6×10^−4^
CH_3_SO_3_^−^ (f)	3.8×10^−3^±0.7×10^−4^	4.5×10^−3^±1.8×10^−4^

[a] Experimental rate constant for the reaction between methyl iodide and pyridine in DMSO at 25 °C is 4.0×10^−2^.

**Figure 1:**
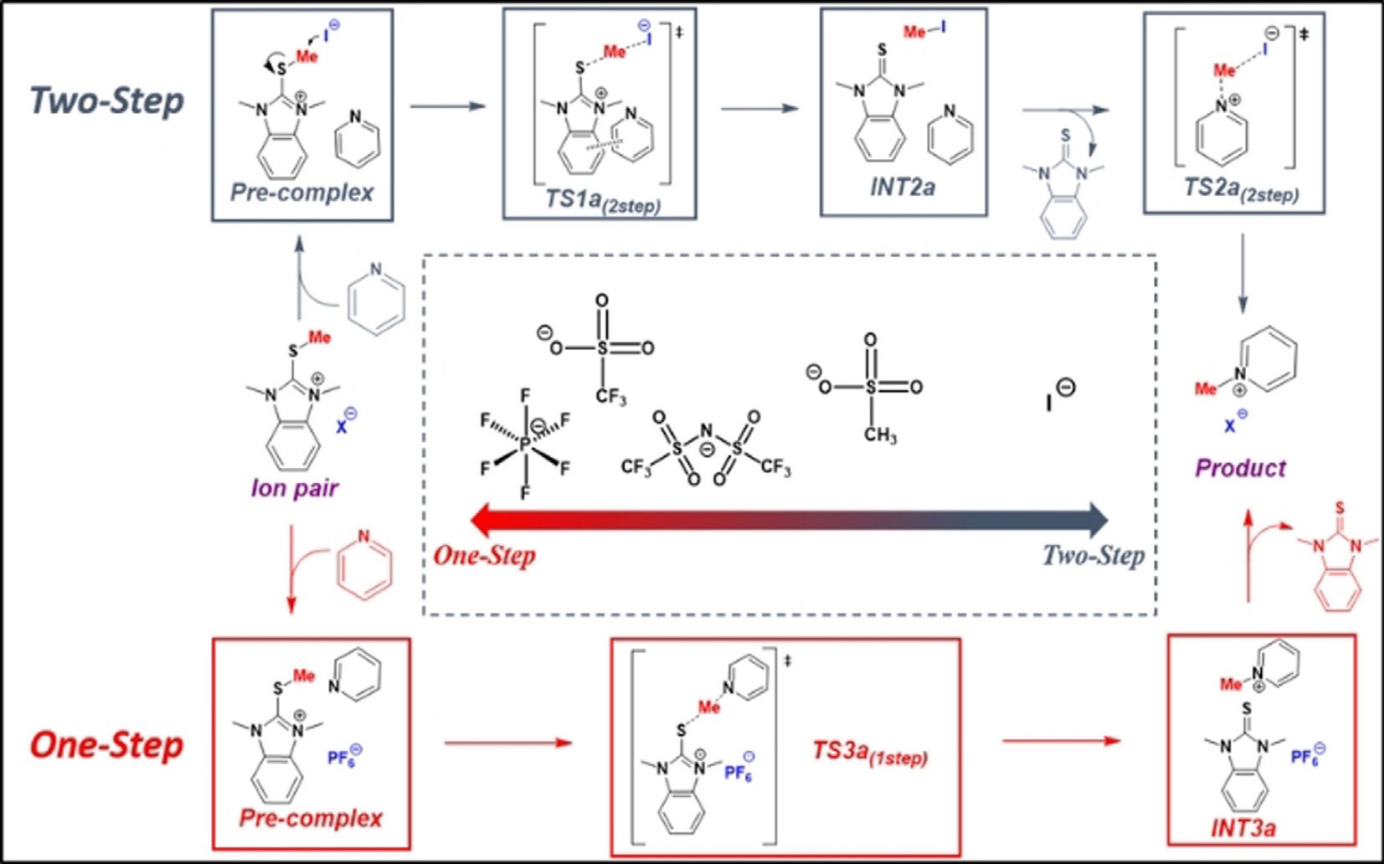
Overview of the two possible reaction paths for the presented salts.

Mesylate and TFSI salts are the least reactive despite being intermediate in nucleophilicity between the others. These results illustrate that there are likely multiple factors contributing to reactivity through possibly two or more different mechanisms. To better understand the anion effect, we have computationally modeled multiple alternative transition states and precomplexes within the potential energy surface using density functional theory (DFT, Figure 1).

### Structure determines conformation, determining mechanism

**Figure 2:**
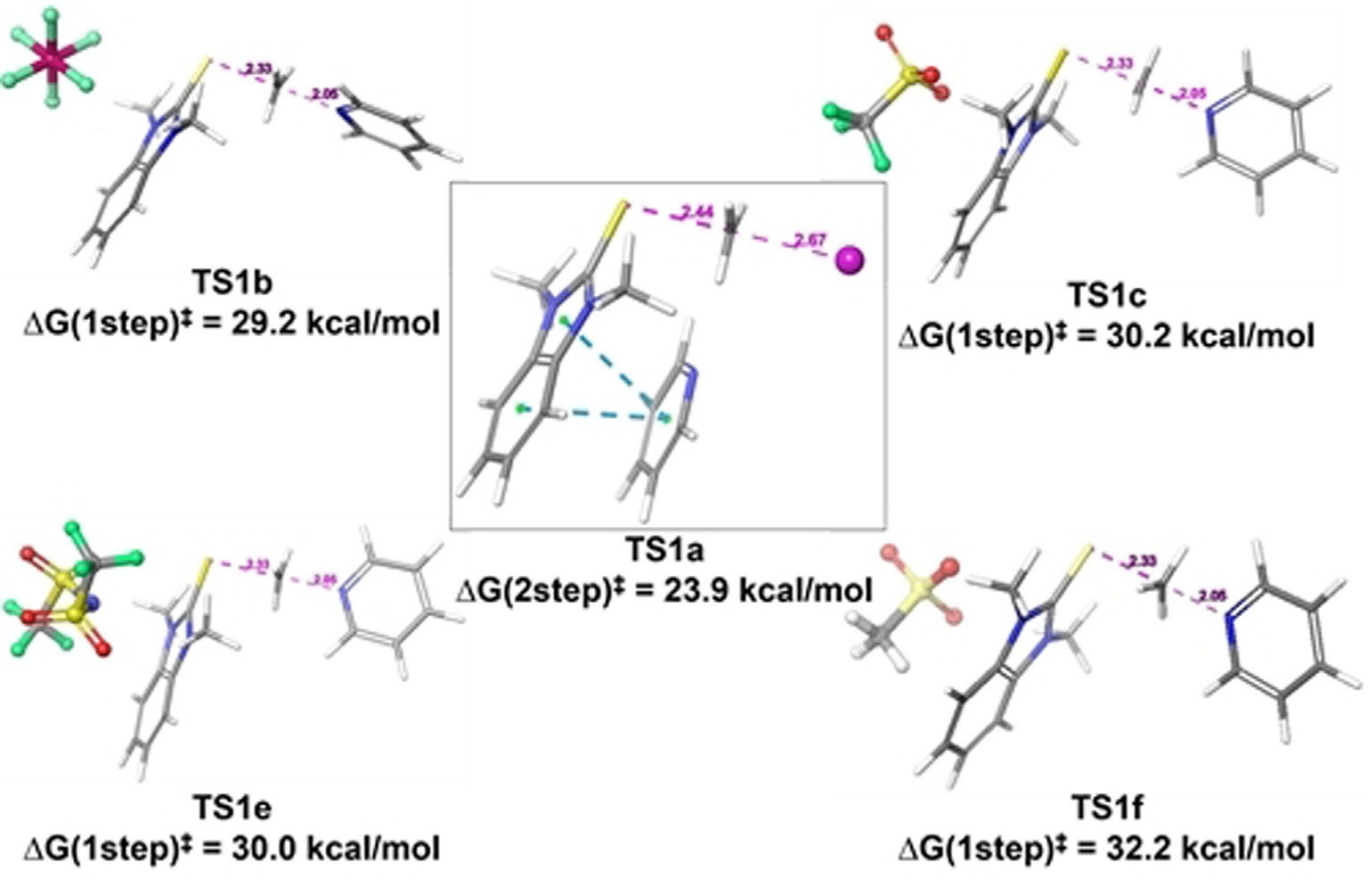
Major transition structures for the (a) competing two‐step iodide‐coordinated, (b) PF_6_^−^ mediated, (c) CF_3_SO_3_^−^, and (d) TFSI mediated concerted type transition states.

**Figure 3:**
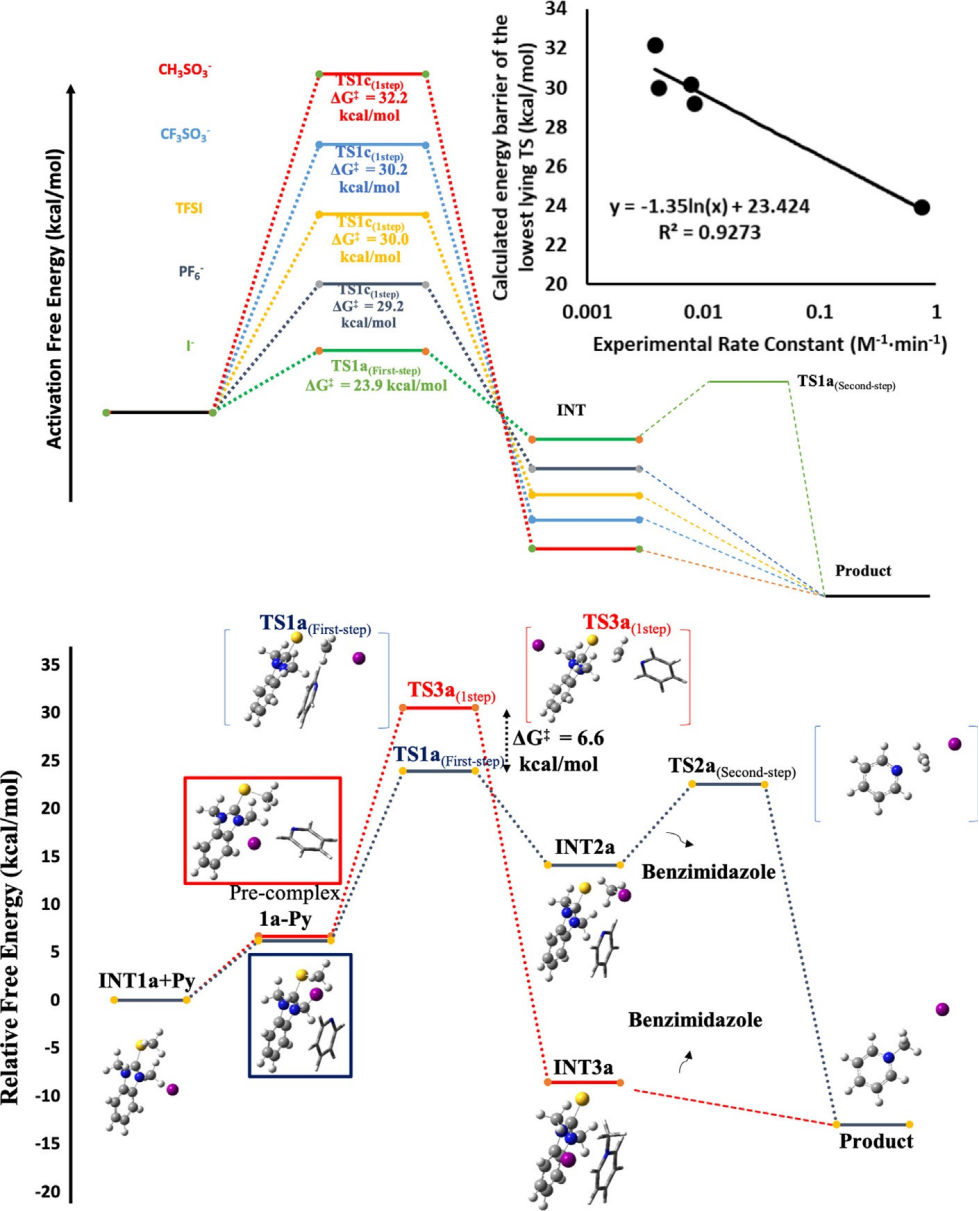
A) Comparison of the rate determining transition state barriers between the counterions **1a**, **1b**, **1c**, **1e**, **1f**. Inset: logarithmic relationship between calculated energy barrier and the experimental rate constant. B) The calculated potential energy surface for both investigated mechanisms for the iodide promoted alkylation process.

Selected lowest‐energy transition states (**TS1 a**–**f‐py**) are provided in Figure 2 (see Supporting Information for all other possible transition states). Each TS geometry differs substantially from the others relative to the positioning of the counterion; however, they fall into one of two broad classes. In the first, the counterion acts as the nucleophile (labelled as **TS2Step**); in the second, the pyridine is directly alkylated by the thioimidazolium cation (labelled as TS1Step). We found transition states for both mechanisms for all complexes, and identified that iodine alone works through the two‐step pathway (with the formation of methyl iodide being rate determining), while the others follow the one‐step route (Figure 3 A). Similar results for iodide‐mediated *N‐*alkylations has been previously found by using a combination of XPS and rheometry.^[29]^ We then computationally probed the minimum energy mechanistic pathways for both mechanisms for three different benzimidazolium salts (Figure 3 B; see Supporting Information for the other pathways).

In all cases, alkylation commences with the complexation of the benzimidazolium ion pair and pyridine (**Pre‐complex 1**, Figure 3 B). For the two‐step pathways, alkyl transfer proceeds by an initial interaction of the methyl group with the coordinated counter anion via a rate‐determining **TS1(First‐Step)** to form counterion alkylated intermediate (**INT2**). The second step proceeds via reorientation of pyridine and its nucleophilic attack on transient **INT2** via **TS2(Second‐Step)**, thereby releasing the benzimidazole. The rate of this two‐step mechanism depends on the nucleophilicity of the counteranion since its attack on the methyl group is rate determining.

The second mechanistic scenario, where the counterion mediates the direct nucleophilic attack of the methyl group by pyridine via **TS3(1Step)**, proceeds through charge‐separated intermediate **(INT3)**, followed by benzimidazole dissociation to provide the alkylated product (Figure 3 and S15).

These mechanisms suggest that strong nucleophilic anions like iodide would favor a two‐step mechanism while highly dissociative anions (like PF_6_^−^), less capable of delocalizing the positive charge on the benzimidazolium (thereby making it more reactive), would favor the one‐step direct alkylation mechanism (Figure 3 A; see Supporting Information). To explain this counterion dependent mechanistic behaviour, we compared the activation barrier differences (Δ*G* ^≠^) between the rate determining TS3(1Step) and TS1(2Step) for each system. The lowest energy transition states for each salt support the mechanistic hypothesis. For example, in the case of iodide, the precomplexed pyridine even assists in better orienting the iodide to capture the methyl group (**TS1 a(2Step)**=23.9 kcal mol^−1^); this route lies 6.6 kcal mol^−1^ lower than the direct attack pathway (**TS3 a(1Step)**, 30.5 kcal mol^−1^, Figure 3). Iodide consequently has by far the lowest energy barrier of any process examined in this study (Table S4), consistent with experiment. This trend was maintained regardless of level of theory or size of basis set. This especially low energy barrier is a result of the pyridine being positioned through attractive interactions with the benzimidazolium. This better orients the iodide to capture the methyl group (**TS1 a(2 step)**=23.9 kcal mol^−1^) in a geometry that sets up the subsequent pyridine alkylation (**TS2 a(2 step)**=22.5 kcal mol^−1^, Figure S15). Direct alkylation for iodide salts is disfavored (Δ*G*(1step)^≠^=30.5, Tables S4 and S15). Iodide is unique as other counterions (CF_3_SO_3_^−^, CH_3_SO_3_^−^, TFSI, and PF_6_^−^) do not act as nucleophiles (Table S4). The least nucleophilic anion PF_6_^−^ greatly favors the one‐step process with **TS3 b** lying 15.5 kcal mol^−1^ below two‐step TS1b. This is consistent with the non‐nucleophilic nature of PF_6_^−^.

The other salts are intermediate: none are as nucleophilic as iodide, and none are as dissociated as PF_6_^−^; the computational model appears to be accurate as the calculated activation energies match the experimental trend (Figure 3 A).

To elucidate the role of the counterion in the one step nucleophilic addition pathway, we focused on the most reactive (PF_6_^−^) and least reactive (CH_3_SO_3_^−^) systems (**TS1 f(1 step)** and **TS1 b(1 step)**). To this end, the electronic changes along the Npy⋅⋅⋅CH_3_ bond forming and H_3_C⋅⋅⋅S bond breaking processes were evaluated using natural bond orbital (NBO) analysis. During nucleophilic attack, significantly better orbital overlap between the donor orbital of nucleophile and the acceptor NBO orbital of the methyl group was observed for the PF_6_^−^ mediated **TS1 b(1 step)** (Δ_*E*NBO_(total)=308.1 kcal mol^−1^) than for CF_3_SO_3_^−^ **TS1 f(1 step)** (Δ_*E*NBO_(total)=130.5 kcal mol^−1^), helping to explain their differential reactivity.

Strong nucleophilic character is essential for a favorable two‐step mechanism. Mesylate is a poor nucleophile and so it would not obviously favor the two‐step process like that of iodide, however, it is certainly more competent than even less nucleophilic PF_6_^−^. In contrast, the one‐step direct alkylation is facilitated by a strongly delocalized counterion that increases the local positive charge on the methyl group, best exemplified by PF_6_^−^. Mesylate again, although charge delocalized, does form a stronger direct interaction with the transferrable methyl group, making this pathway more inaccessible too. Sluggish under both mechanisms, mesylate results in the lowest reaction rate.

### Orientation and anion nature define interaction energy and in turn ion‐pair physical behavior

**Figure 4:**
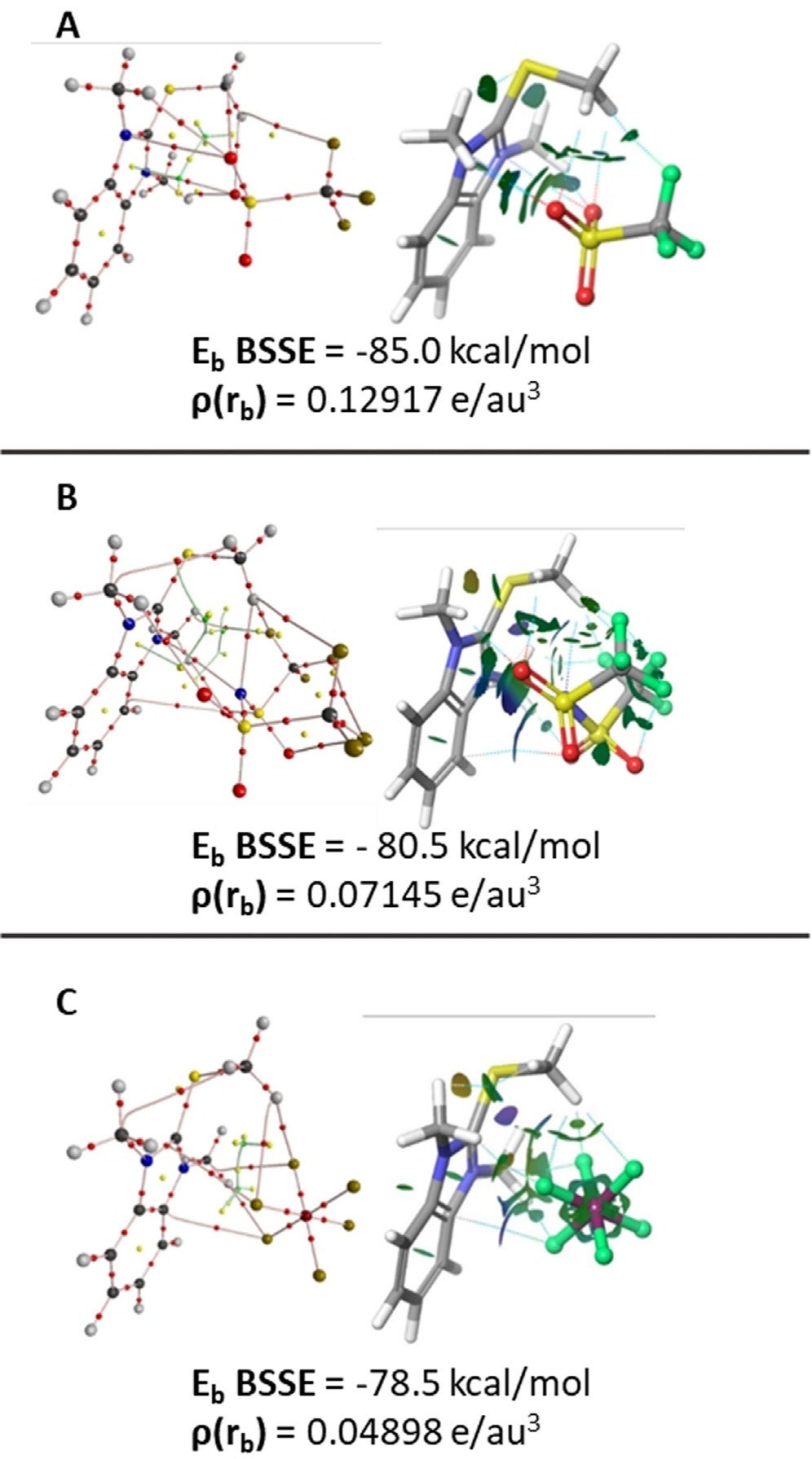
The distribution of bond, ring, and cage critical points in the inner cavity of a) **1 c** (triflate), b) **1 e** (TFSI), c) **1 b** (PF6‐) using QTAIM and non‐covalent isosurfaces by NCI as well as the BSSE corrected binding energy calculated at **ω**b97xd/6–311G(d,p) level of theory.

To better correlate the energies of the one‐step mechanisms with the structural parameters, we performed a conformational energy search (see Supporting Information) and an in‐depth DFT (ωB97X‐D/6–311G(d,p) study of the counter‐ion coordinated pre‐complexes of PF_6_^−^ (strongly reactive), CF_3_SO_3_^−^ (medium reactive) and TFSI (weakly reactive) mediated transition state structures **(TS3 b(1Step)**, **TS3 c(1Step)**, and **TS3 e(1Step)**). While it is likely that the active species is not a single ion‐pair precomplex but rather an aggregate due to the high reaction concentrations (462 mm), this is likely not a critical parameter that needs to be considered for these energy calculations. Others have extensively investigated the effect of ionic liquid aggregation/self‐assembly on bulk physical and chemical properties,[^30^, ^31^, ^32^, ^33^] and in most cases found that varying the number of molecules in the cluster had little effect on the interactions between the components of a single ion pair or the coordination capability of the anion. Consequently, we focused our efforts on the solvated optimized (IEFPCM model; DMSO) single pre‐complexes of **1 b** (PF_6_^−^), **1 c** (triflate), and **1 e** (TFSI) using QTAIM based analysis at the ωB97X‐D/6–311G(d,p)/SDD for iodine. These calculations indicate that in the optimal binding mode, the anions interact with the benzimidazolium cation's S‐Me moiety through a rich network of attractive noncovalent interactions including hydrogen bonds and attractive X–X (X=O, N, or F) contacts (Figure 4). In **1 c** and **1 e**, the assemblies benefit from the specific orientation of the anion since the sulfonic functionalities form a series of interactions via the O and N atoms with the benzimidazole ring, while the F atoms in both the triflate and TFSI associate with the aromatic protons and the alkyl moieties of the cation. In contrast, PF_6_^−^ is a non‐coordinating anion and can only establish weak electrostatic interactions, thus limiting the strength attractive forces between the cation and anion.

The interaction strength of the non‐covalent interactions between thiobenzimidazolium and TFSI, triflate and PF_6_^−^ can be described by the ion pair binding energy (Eb) and its components: electron correlation effects (EMP2) and electron density strength (Ex, Figure S16). Analysis of the density parameters at the bond critical points (BCP) of the key interactions in the binary precomplexes also highlights the same order affinity: triflate forms stronger interactions (total electron density at BCPs, triflate 0.13 e/au3) than the TFSI (0.071 e/au3) or PF_6_^−^ systems (0.049 e/au3). This indicates that PF_6_^−^ has the weakest ion‐pairing binding energy of the three, with triflate being the strongest.

These interactions explain the higher calculated binding energies of **1 c** and **1 e** (−85.0 and 80.5 kcal mol^−1^, respectively) than in the weaker bound PF_6_^−^ **1 b** (−78.5 kcal mol^−1^). QTAIM analysis allows us to more closely evaluate the density characteristics of the interactions in these three systems, confirming that the intermolecular interactions are non‐covalent (see Supporting Information for a detailed discussion). In addition, several key X–X contacts (N–N, N–O, N–F, F–S, O–S and F–Car, Figure S17) clearly play an important role in properly orienting all three anions within the cavity. In particular, the N–N contacts in **1 e** (Figure S17) show the same topological behaviour as the weak H‐bonds. The components of the Laplacian for the three systems indicate that the electrostatic component of the dipole–dipole and polarization interactions are more important than the charge transfer between cation and anion, for TFSI, as a result of its increased delocalization, and triflate to a lesser extent. For PF_6_^−^, charge transfer and electrostatic attraction both contribute more equally (and weakly); this is completely consistent with the energetic analysis described above (Figure 4; Table S6).

The interaction energy comprises several orthogonal components:^[34]^ the electrostatic energy representing the affinity between the charge distributions of undistorted monomers Ees; Eex the Pauli exchange‐repulsion energy, Epl the polarization term representing the Coulombic interaction between the distorted ions, and the Morokuma delocalization, or charge transfer term, Ect. To evaluate the nature and strength of the binding interactions in **1 b**, **1 c**, and **1 e** complexes using a virial theorem approach, the energy density contributions were further analyzed using QTAIM. The total electronic energy density at the interaction BCP (H(rb)) can be deconstructed into the Laplacian, local electron energy at the BCP. 1/4 ∇2ρ(rb) (correlated with Ect and Eex), and the kinetic energy density at the BCP −Gb (correlated with Ees and Epl). Variations of 1/4 r (∇2ρ(r)+(−G(r)) and the H(b) energy densities were evaluated as a function of the electronic charge density (ρ(r)) at the BCP (Figure S16 and accompanying text). Plotting (−G(rb)), 1/4 ∇2ρ(rb) and H(rb)) as a function of the ρ(rb) values (ranging from 0.0019 to 0.0113) for the three precomplexes (Figure 4) shows that the Laplacian (ranging from 0.0092 to 0.0473, Table S5, Figure S17) increases proportionally to an increase in the strength of the individual cation anion interaction electron density, while the kinetic energy density, (−G(rb)) decreases. The overall local electronic energy density, H(rb), increases slightly as the electron density increases. The energy densities and the interaction energy components correlate better (R2) with the ρ(rb) in **1 c** and 1e than with the **1 b** precomplex although the agreement is good in all cases. In addition, (−G(rb)) and ρ(rb) correlate better than the Laplacian for **1 c** and **1 e** indicating that the electrostatic component of the dipole‐dipole and polarization interactions are more important than the charge transfer between cation and anion, especially in the case of the TFSI because of its increased delocalization. However, this trend is not significant for PF_6_^−^ as a nearly ideal linear correlation was found between both (−G(rb)) and 1/4 ∇2ρ(rb) and bonding strength suggesting a more balanced contribution from both components.

These independent analyses all support a single conclusion: the PF_6_^−^ coordinated precomplex is more loosely associated than either the TFSI or triflate ones. This is because the interactions between the anions’ individual atoms and the benzimidazolium are tighter for the coordinating ions; especially the O–N interactions. PF_6_^−^ on the other hand can both dissociate more easily and has a lower tendency to form higher order aggregates.

Together these phenomena result in PF_6_^−^ providing a loose ion pair, facilitating the repositioning of the pyridine, and consequently accelerating alkylation. Our calculations show that the anion has a crucial influence on the alkylation mechanism and reactivity of benzimidazolium cations. However, this entire mechanistic discussion assumes that ion‐pairs or aggregates are present in solution. The model would be inaccurate should the ions be fully solvated. In such a scenario, the identity of the anion would not matter. However, this complication can be readily addressed: ion‐pair binding energies not only describe the stability of the complex, but they also can be used to predict experimental viscosity and conductivity. The short‐range dispersion component of the ion‐pair binding energy correlates better with conductivity and viscosity properties of ion pairs while the long‐range electrostatic and polarization components correlate with the melting point of ILs.^[35]^ This motion must also be considered for a better understanding of the reactivity of these systems. Strongly paired cation‐anion complexes with few inter‐cluster interactions will show lower conductivities and viscosities with motion largely determined by electron dispersion; however, the existence of ion‐pairs or higher order aggregates has not yet been experimentally demonstrated with these compounds. Experiments were conducted in DMSO, which unlike water generally favors the formation of ionic liquid‐rich clusters even at concentrations below 10 wt %.[^36^, ^37^] The formation of aggregates would be consistent with this explanation of the observed reactivity.

### Diffusion and aggregation state of benzimidazolium salts in DMSO

**Figure 5:**
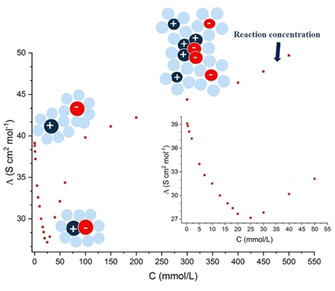
Molar conductivity of 1a as a function of concentration in DMSO. At very low concentrations, ions exist mainly as ionized species resulting in high molar conductivity. As the concentration further increases, neutral ion‐pairs form resulting in a drop in molar conductivity before increasing again as charged aggregates predominate. Alkylation reactions performed in this study is at a concentration where aggregates are present (462 mm).

To better understand the aggregation state of these salts and explore their anion‐dependency, we used a combination of conductivity measurements and DOSY NMR spectroscopy.^[38]^ Together this allows us to determine aggregation state as a function of concentration and an accurate determination of ion diffusivity and solvodynamic radius. Absolute conductivity increases as the concentration of salts **1 a**, **1 b**, **1 c**, and 1e in DMSO increases; however, replotting molar conductivity reveals an initial drop in conductivity with greater salt concentrations before rising again (Figure 5; See Supporting Information for full data). This is a result of the transition from freely solvated/ionized species to neutral‐contact pairs being formed, thus lowering the molar conductivity of the solution.^[39]^ While there is an equilibrium between ion‐pairs and solvent‐separated ions at low concentrations, the balance of the equilibrium depends on the concentration and identity of the counterions.^[40]^ Minimum molar conductivity is observed at: 10 mm (PF_6_^−^; **1 b**), 20 mm (TFSI; **1 e**), 25 mm (I^−^; **1 a**), and 30 mm (CF_3_SO_3_^−^; **1 c**), which is considerably lower than the concentrations used in this study for reacting with pyridine (462 mm). At such high concentrations an equilibrium^[40]^ between large charged aggregates, hydrogen bonded assemblies, charged triple ions and ion pairs is established.[^41^, ^42^] These results provide evidence for the presence of ion‐aggregates at reaction concentrations indicating that cation‐anion interactions likely affect reactivity while the comparatively narrow range for ion‐pair formation for **1 b** is consistent with weaker ion‐pair interactions that favors higher order aggregates as opposed to tightly bound pairs. These results also confirm that calculations treating these systems as ion pairs (as models of higher order aggregates) is the appropriate method. Unlike fully solvated ions, ion‐pairs and aggregates move slower in solution and therefore possess lower diffusion rates different and radii. To determine these parameters and provide complementary evidence for their formation, we performed ^1^H and ^19^F{^1^H} DOSY experiments on compounds **1 c**, **1 e** and **1 b** at both their minimum molar conductivities, and at our normal reaction concentration (462 mm) in [D_6_]DMSO (Tables S1–S2).^[43]^ The diffusion coefficients for the anions in all measured salts are smaller than those reported in literature for the single anions at either concentration.[^44^, ^45^, ^46^] This suggests the presence of bigger and thus slower moving objects in solution, such as strongly coordinated ion pairs or higher‐order aggregates instead of smaller, separated ions. As shown in Tables S1 and S2, the values for the cations are larger than those expected for isolated TFSI, PF_6_^−^, and CF_3_SO_3_^−^ anions.[^46^, ^47^, ^48^] The PF_6_^−^ anion has the highest diffusion coefficients under both conditions (4.28×10^−10^ m^2^s^−2^ at the conc. of 462 mm), while triflate shows the lowest diffusion of the test anions (3.23×10^−10^ m^2^s^−2^). These results are consistent with our computational model that PF_6_^−^ forms loose ion‐pairs with benzimidazolium due to its propensity to form weak electrostatic interactions, while triflate promotes several attractive non‐covalent interactions via the O and N atoms, resulting in tighter ion‐pairs. At low concentrations, all salts exhibit higher diffusion coefficients and smaller ionic radii than at 462 mm (e.g. 9.7×10^−10^ m^2^ s^−2^ against 4.3×10^−10^ m^2^ s^−2^ for PF_6_^−^ anion), consistent with the formation close contact ionic pairs and low solvation and with the low molar conductivity due to the formation of the overall neutral ion pairs. At higher concentrations we observed a decrease in the diffusion coefficients for both cations and anions compared to the same salts at a low concentration, and an increase in the ionic radii. While the increase in the radii indicates greater solvation of the ions Slower diffusion reflects the formation of bigger and charged aggregate of ions typical of ILs in DMSO, consistent with the well documented tendency of DMSO to promote the formation of solvent‐surrounded ion pairs over isolated free ions.^[36]^ As aggregation is concentration‐dependent, we can infer that the alkylator reactivity is also concentration‐dependent as the reaction proceeds through a minimum three‐component system. In this regard we measured the rate constants for the reaction of **1 c** with pyridine at the concentration where its molar conductivity is at its lowest (30 mm), and at 250 mm and 600 mm. We found that there is a concentration dependency on the rate constant with values ranging from 6.3×10^−4^ m ^−1^ min^−1^ at 30 mm to 1.3×10^−2^ m ^−1^ min^−1^ at 600 mm (Figure S13). This provides strong evidence that different aggregation states influence the activation energy of the reaction as described in the previous sections; however more investigations will provide further details to explain these observations, as ion pair‐dependent reactions represent an interesting tool in organic transformations and catalysis.^[49]^

## Conclusions

Often when reactivity differs based on the nature of the counterion the discussion focuses around p*K*_a_ values or a crude estimate of the relative coordinating ability of the anion. Here we reveal that this model is too simplistic and instead introduce a description of cation‐anion interactions based on experimental and computational analysis that explains trends in reactivity derived from a multivariant approach. Even for an apparently trivial transformation: two distinct mechanisms compete the methylation of pyridine with a cationic thiobenzimidazolium molecule, either a one‐step or two‐step process, and that reactivity in general is governed by cation‐anion pairing effects.

In the case of this system, greater reactivity can be obtained with highly nucleophilic counterions since they form a reactive alkylating intermediate, thus following a two‐step mechanism. Meanwhile reasonable activity can be obtained using a loosely‐associated counterion such as PF_6_^−^ since it can facilitate the interaction of pyridine with the benzimidazolium cation and promote a one‐step mechanism. Thus favoring neither reaction mechanism Anions such as mesylate are neither good nucleophiles nor assist pyridine, mesylate was the slowest of all tested anions. The reaction rate is further influenced by H‐bonds, electrostatic, polarization, and dispersive interactions around the reactive site caused by the specific interplay between N, O, and F atoms on the anion and the cation. By coupling molar conductivity and DOSY experiments with QM‐based computational models, we show that ion pairs and/or aggregates are formed and that these ion‐aggregates are likely responsible for the alkylation reaction in DMSO. Unlike free, cations present within an aggregate are strongly influenced by nearby. This model has been examined for benzimidazolium‐based alkylators fully solvated cations in solution, however our approach can be applied to other commonly used salts. The effect of different halides was not examined here and would be of great interest given the unique behaviour of the iodide. The spectator anion, especially in organic solvents, is not so innocent and can be involved as an important component of the overall reaction.

## Conflict of interest

The authors declare no conflict of interest.

## Supporting information

As a service to our authors and readers, this journal provides supporting information supplied by the authors. Such materials are peer reviewed and may be re‐organized for online delivery, but are not copy‐edited or typeset. Technical support issues arising from supporting information (other than missing files) should be addressed to the authors.

Supplementary
