# Supplementary material for: Not‐So‐Innocent Anions Determine the Mechanism of Cationic Alkylators
Source: Chemistry. 2021 Jan 18;27(10):3440–8. doi: 10.1002/chem.202004208 (PMC7898791; doi:10.1002/chem.202004208)
Supplement: Supplementary file 1 — Supplementary [file CHEM-27-3440-s001.pdf]

Chemistry–A European Journal

Supporting Information

Not-So-Innocent Anions Determine the Mechanism of Cationic Alkylators

S. Maryamdokht Taimoory+,[a, b] Vincenzo Alessandro Cataldo+,[c] Andreas Schäfer,[d] John F. Trant,*[a] and Ryan Guterman*[c]

Contains 106 pages, Experimental and Computational Characterization Details, 16 Supporting Figures, 6 Supporting Tables, and 2 Supporting Schemes.

Table of Contents

Experimental Procedures……………………………..…………………………………………. 3

Reagents ……………………………………………………………………………………… 3

Kinetics experimental procedure …………………………………...…………………………… 4

Synthesis ……………………………………………………………...………………………4

Results and discussion ………………………………………………………..…………………… 9

Computational study………………………………………………..………………………….. 23

Selected DFT calculated transition states, complex geometries & thermochemical data……..………30

Author Contributions…………………………………..…………………………………………105

Experimental Procedures

Materials and Instrumentation

1,3-Dimethylbenzoimidazole-2-thione was purchased from RareChemicals and used as received. Potassium hexafluorophosphate (≥99%), caffeine (99.9%, anhydrous), sulfur (99.98%), pyridine (99.9%, anhydrous) and diethyl ether (≥99.9%) were purchased from Sigma-Aldrich and used as received. Deuterated DMSO was purchased from Sigma-Aldrich and stored over 4 Å molecular sieves. Methyl iodide (99%) and methyl benzenesulfonate (98%) were purchased from Alfa Aesar and used as received. Acetonitrile (99.9%) and methanol (99.9%) were purchased from Merck and used as received. Methyl methanesulfonate (99%) and methyl trifluoromethanesulfonate (96%) were purchased from Acros and used as received. 2-propanol (100%) was purchased from VWR Chemicals and used as received. Potassium carbonate (99.92%, anhydrous) was purchased from Fischer-Scientific and used as received. Lithium bis(trifluoromethane)-sulfonylimide (LiTFSI, 99%) was purchased from IOLITEC and used as received. ^{1}H, $^{13}C\{^{1}H\}$ and $^{19}F\{^{1}H\}$ NMR spectra were collected on a Bruker DPX-400 spectrometer for the characterization of all compounds, while the alkylation kinetic experiments were conducted on a Varian 600 MR with a OneNMR probe. Spectra were acquired every hour for up to 12 h. DOSY spectra were performed on JEOL ECZ 600 spectrometer equipped with a SuperCOOL™ cryo probe outer coil tuned to Proton or Fluorine and a z-axis gradient coil. The standard JEOL pulse sequence (bpp-led-dosy-pfg) was used for ^{19}F DOSY experiments. For ^{19}F diffusion measurements, the delay δ was set to 2 ms and Δ was set to 400 ms. The individual slices of pseudo-2D diffusion spectra were phased. The diffusion coefficients were obtained from the signal attenuation curves of individual DOSY peaks. Elemental analysis (EA) was conducted on a VarioMICRO instrument. Thermogravimetric analysis (TGA) experiments were conducted using a Netzsch TG209-F1 apparatus with a heating rate of 10 K min^{-1} under nitrogen flow. An aluminum crucible was used for the measurement of 10 mg of sample under a flow of nitrogen (10 mL/min) and a purge flow of 10 mL min^{-1}. The samples were heated at a heat rate of 10 K min^{-1} to 600 °C. Data were recorded and analyzed by the Proteus (6.0.0) software package. Reported decomposition temperatures were taken at 1% mass loss. Differential scanning calorimetry (DSC) experiments were performed on a PerkinElmer DSC-1 instrument at a heating/cooling rate of 10 K min^{-1} under nitrogen flow and cycled five times. Melting points and glass transitions were acquired from the final heating cycle. If a melting point could not be observed, it was determined by visual observation using a Thermo Scientific 9300 melting point apparatus. Conductivity experiments were performed on a Mettler Toledo S30 SevenEasy conductivity apparatus using an InLab 751-4mm conductivity probe.

Kinetics Experimental Procedure

A 1:1 molar ratio of alkylating agent (compounds from series **1** and **2**) and pyridine was dissolved in DMSO-d_6 (0.4 mL) and added to a standard NMR tube. The sample was then heated to reaction temperature (90 °C) and analyzed in situ every hour by ^{1}H NMR spectroscopy for up to 13 h. Pyridine conversion was determined by comparing the integration values of pyridine (δ = 7.99 or 7.35) to 1-methylpyridinium product (δ = 8.13), while alkylating agent conversion was determined by comparing the integration values of the thioimidazolium salt to the thione product. Second-order rate constants were obtained by plotting 1/[Pyr]$_t$ as a function of time and measuring the slope. In all cases, we found the rate of consumption for the alkylator to be almost equal to the rate of production of 1-methylpyridinium.

Synthesis

Synthesis of compounds followed a modified previously described procedure[1] and it is described in Scheme S1 and S2.

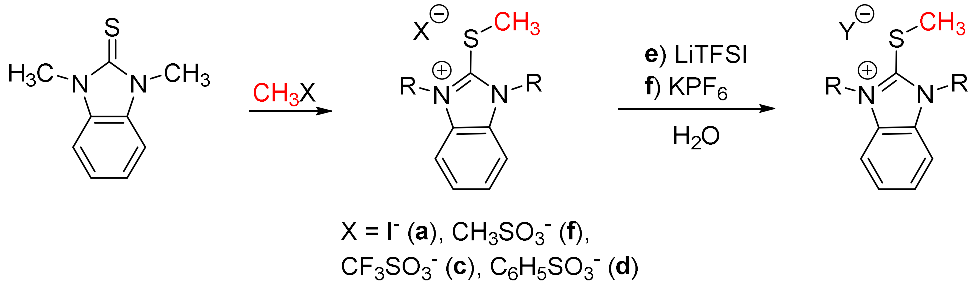

Scheme S1. Synthetic scheme for compounds **1a-f**.

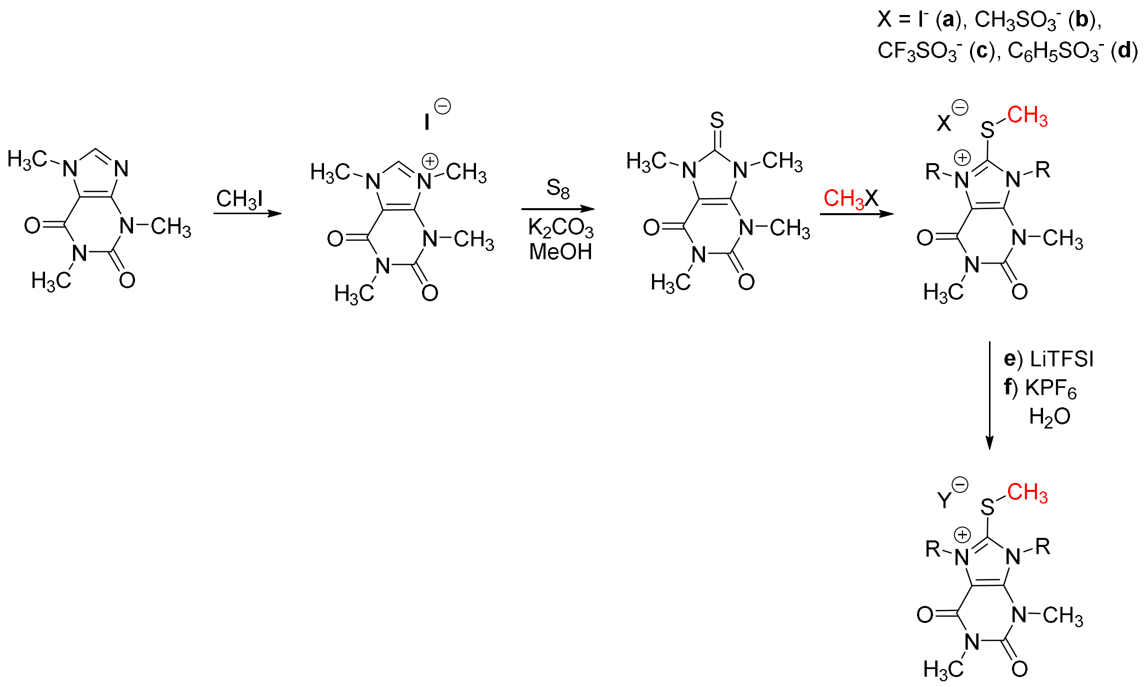

Scheme S2. Synthetic scheme for compounds **2a-f**.

1,3-dimethyl-2-(methylthio)-benzoimidazolium Iodide (**1a**): 1,3-dimethylbenzimidazole-2-thione (2.53 g, 1.42 mmol) was added to acetonitrile (35 mL) and stirred for 10 minutes before adding methyl iodide (3.02 g, 2.21 mmol). The solution was stirred for 24 hours, concentrated by rotary evaporation, and precipitated in diethylether (100 mL). The solution was then decanted and volatiles removed *in-vacuo*, leaving a white powder identified as **1a** (3.01 g, 84 %). ^{1}H-NMR (400 MHz, DMSO-d6): δ = 2.74 (s, 3H, S-CH_3), 4.14 (s, 6H), 7.71 (m, 2H), 8.08 (m, 2H). ^{13}C-NMR (100 MHz, DMSO-d6): δ = 17.5 (s), 33.5 (s), 113.3 (s), 126.7 (s), 132.1 (s), 149.9 (s). T_{dec} = 118.7 °C; T_g = 88.5 °C; T_m = 108.0-108.2 °C. EA calcd. for $C_{10}H_{13}IN_2S$: C, 37.51; H, 4.09; N, 8.75; S, 10.01. Found: C, 37.80; H, 4.10; N, 8.77.

1,3-dimethyl-2-(methylthio)-benzoimidazolium Methanesulfonate (**1b**): 1,3-dimethylbenzimidazole-2-thione (0.41 g, 2.33 mmol) was added to acetonitrile (15 mL) and stirred for 10 minutes before adding methyl methanesulfonate (1.36 g, 9.59 mmol). The solution was stirred for 24 hours in an oil bath at 60°C, concentrated by rotary evaporation, and precipitated in diethylether (100 mL). The solution was then decanted and volatiles removed *in-vacuo*, leaving a white powder identified as **1b** (0.48 g, 71 %). ^{1}H-NMR (400 MHz, DMSO-d6): δ = 2.26 (s, 3H), 2.74 (s, 3H, S-CH_3), 4.14 (s, 6H), 7.71 (m, 2H), 8.09 (m, 2H). ^{13}C-NMR (100 MHz, DMSO-d6): δ = 17.1 (s), 33.3 (s), 39.7 (s), 113.4 (s), 126.8 (s), 132.2 (s), 150.0 (s). T_{dec} = 170.8 °C; T_g = not obs; T_m = 150.2-150.5 °C. EA calcd. for $C_{11}H_{16}N_2O_3S_2$: C, 45.81; H. 5.59; N, 9.71. Found: C, 45.94; H. 5.62; N, 9.72.

1,3-dimethyl-2-(methylthio)-benzoimidazolium Trifluoromethanesulfonate (**1c**): 1,3-dimethylbenzimidazole-2-thione (0.21 g, 1.18 mmol) was added to dichloromethane (10 mL) in a glove box before adding methyl trifluoromethanesulfonate (0.20 g 1.23 mmol). The solution was stirred for 24 hours and precipitated twice in diethylether (100 mL). The solution was then decanted and volatiles removed *in-vacuo*, leaving a white powder identified as **1c** (0.38 g, 94%). ^{1}H-NMR (400 MHz, DMSO-d6): δ = 2.72 (s, 3H, S-CH_3), 4.13 (s, 6H), 7.72 (m, 2H), 8.06 (m, 2H). ^{19}F-NMR (376 MHz, DMSO-d6): δ = -77.79(s). ^{13}C-NMR (100 MHz, DMSO-d6): δ = 17.1 (s), 33.2 (s), 113.3 (s), 126.8 (s), 132.1 (s), 150.0 (s). T_{dec} = 97.4 °C; T_g = not obs; T_m = 89.5-89.7 °C. EA calcd. for $C_{11}H_{13}F_3N_2O_3S_2$: C, 38.59; H. 3.83; N, 8.18. Found: C, 38.41; H. 3.71; N, 8.02.

1,3-dimethyl-2-(methylthio)-benzoimidazolium Benzenesulfonate (**1d**): 1,3-dimethylbenzimidazole-2-thione (0.99 g, 2.33 mmol) was added to acetonitrile (15 mL) and stirred for 10 minutes before adding methyl benzenesulfonate (1.36 g, 9.59 mmol). The solution was stirred for 24 hours in an oil bath at 50°C, concentrated by rotary evaporation, and precipitated in diethylether (100 mL). The solution was then decanted and volatiles removed *in-vacuo*, leaving a white powder identified as **1d** (1.69 g, 86 %). ^{1}H-NMR (400 MHz, DMSO-d6): δ = 2.71 (s, 3H, S-CH_3), 4.11 (s, 6H), 7.29 (m, 3H), 7.56 (m, 2H), 7.68 (m, 2H), 8.04 (m, 2H). ^{13}C-NMR (100 MHz, DMSO-d6): δ = 17.1 (s), 33.2 (s), 113.3 (s), 125.4 (s), 126.7 (s), 127.6 (s), 128.3 (s), 132.1 (s), 148.3 (s), 150.0 (s). T_{dec} = 141.6 °C; not obs; T_m = 120.4-120.5 °C. EA calcd. for $C_{16}H_{18}N_2O_3S_2$: C, 54.84; H. 5.18; N, 7.99. Found: C, 54.63; H, 5.16; N, 7.83.

1,3-dimethyl-2-(methylthio)-benzoimidazolium Bis(trifluoromethylsulonyl)imide (**1e**): Compound **1a** (0.25 g, 0.78 mmol) was dissolved in deionized water (10 mL) followed by the dropwise addition of LiTFSI (0.29 g, 1.00 mmol) in deionized water (5 mL). The solution was stirred for 24 hours and the precipitate rinsed with deionized water (3x5 mL) and dried *in-vacuo* to isolate a white powder identified as **1e** (0.23 g, 63%). ^{1}H-NMR (400 MHz, DMSO-d6): δ = 2.72 (s, 3H, S-CH_3), 4.14 (s, 6H), 7.72 (m, 2H), 8.06 (m, 2H). ^{19}F-NMR (376 MHz, DMSO-d6): δ = -78.8 (s). ^{13}C-NMR (100 MHz, DMSO-d6): δ = 17.1 (s), 33.2 (s), 113.3 (s), 119.7 (q, $^1J_{C\text{-}F}$ = 320 Hz), 126.9 (s), 132.3 (s), 145.0 (s). T_{dec} = 265.9 °C; T_g = 13.9 °C; T_m = 120 °C. EA calcd. for $C_{12}H_{13}F_6N_3O_4S_3$: C, 30.44; H, 2.77; N, 8.88. Found: C, 30.72; H. 2.72; N, 8.90.

1,3-dimethyl-2-(methylthio)-benzoimidazolium Hexafluorophosphate (**1f**): Compound **1a** (0.27 g, 0.85 mmol) was dissolved in deionized water (10 mL) followed by the dropwise addition of KPF_6 (0.20 g, 1.10 mmol) in deionized water (5 mL). The solution was stirred for 24 hours and the precipitate rinsed with deionized water (3x5 mL) and dried *in-vacuo* to isolate a white powder identified as **1f** (0.16 g, 58%). ^{1}H-NMR (400 MHz, DMSO-d6): δ = 2.72 (s, 3H, S-CH_3), 4.14 (s, 6H), 7.73 (m, 2H), 8.06 (m, 2H). ^{19}F-NMR (376 MHz, DMSO-d6): δ = -69.7 (s), -71.6 (s) ^{13}C-NMR (100 MHz, DMSO-d6): δ = 17.1 (s), 33.2 (s), 113.3 (s), 126.8 (s), 132.2 (s), 150.0 (s). T_{dec} = 292.7 °C; T_g = not obs; T_m = 172.3-172.6 °C. EA calcd. for $C_{10}H_{13}F_6N_2PS$: C, 35.51; H, 3.87; N, 8.28. Found: C, 35.72; H. 3.82; N, 8.30.

1*H*-Purinium, 3,7-dihydro-1,3,7,9-tetramethyl-2,6-dioxo-1,3,7,9-Tetramethyl-8-thioxo-3,7,8,9-tetrahydropurine-2,6-dione (S2): Compound S2 was prepared according to a modified literature procedure (Bredereck, H.; Kupsch, G.; Wieland, H. *Chem. Ber*. 1959, 92, 566−582.). Compound R7 (11.46 g, 34.1 mmol) was dissolved in methanol (200 mL), followed by the addition of sulfur (1.64 g, 51.15 mmol) and potassium carbonate (9.42 g, 68.2 mmol). The mixture was stirred for 48 h, and volatiles were removed by rotary evaporation. The residue was rinsed with deionized water (3 × 50 mL) and recrystallized from 2-propanol, and volatiles were removed in vacuo leaving a white crystals identified as S2 (3.52 g, 43%). ^{1}H NMR (400 MHz, DMSO-d6): δ = 3.23 (s, 3H), 3.71 (s, 3H), 3.79 (s, 3H), 3.79 (s, 3H), 3.96 (s, 3H). Tm = 267.4−269.3 °C.

1,3,7,9-Tetramethyl-8-(methylthio)-2,6-dioxo-2,3,6,9-tetrahydropurinium Iodide (**2a**): Compound S2 (1.01 g, 4.20 mmol) was combined with methyl iodide (5.96 g, 42.03 mmol) in acetonitrile (20 mL) and stirred for 24 hours at room temperature. Volatiles were then removed in vacuo leaving a yellow powder identified as compound **2a**. (1.57 g, 98%). ^{1}H NMR (400 MHz, DMSO-d6): δ = 2.59 (s, 3H, S−CH3), 3.29 (s, 3H), 3.78 (s, 3H), 4.18 (s, 3H), 4.23 (s, 3H). ^{13}C NMR (100 MHz, DMSO-d6): δ = 17.9 (s), 28.6 (s), 32.2 (s), 35.8 (s), 36.6 (s), 108.7 (s), 140.1 (s), 145.9 (s), 150.00 (s), 152.90 (s). Tdec = 126.8 °C; Tg = not obs; Tm = not obs. EA calcd for $C_{10}H_{15}IN_4O_2S$: C, 31.42; H. 3.96; N, 14.66. Found: C, 31.80; H, 3.72; N, 14.69.

1,3,7,9-Tetramethyl-8-(methylthio)-2,6-dioxo-2,3,6,9-tetrahydropurinium Methanesulfonate (**2b**): Compound S2 (1.00 g, 4.16 mmol) was added to acetonitrile (15 mL) and stirred for 15 minutes before adding methyl methanesulfonate (1.14 g, 10.40 mmol). The solution was stirred for 24 hours in an oil bath at 60°C, concentrated by rotary evaporation, and precipitated in diethylether (200 mL). The solution was then decanted and volatiles removed *in-vacuo*, leaving a white powder identified as **2f** (1.11 g, 76 %). ^{1}H-NMR (400 MHz, DMSO-d6): δ = 2.26 (s, 3H), 2.60 (s, 3H, S-CH_3), 3.78 (s, 3H), 4.18 (s, 3H), 4.24 (s, 3H). ^{13}C-NMR (100 MHz, DMSO-d6): δ = 17.6 (s), 28.6 (s), 32.1 (s), 35.7 (s), 36.3 (s), 39.7 (s), 108.6 (s), 140.3 (s), 145.9 (s), 150.1 (s), 153.0 (s). T_{dec} = not obs; T_g = not obs; T_m = not obs. EA calcd. for $C_{11}H_{18}N_4O_5S_2$: C, 37.70; H. 5.18; N, 15.99. Found: C, 38.01; H. 5.13; N, 16.06.

1,3,7,9-Tetramethyl-8-(methylthio)-2,6-dioxo-2,3,6,9-tetrahydropurinium Trifluoromethanesulfonate (**2c**): Compound S2 (0.85 g, 3.54 mmol) was added to dichloromethane (15 mL) in a glove box before adding methyl trifluoromethanesulfonate (0.58 g, 3.56 mmol). The solution was stirred for 24 hours and precipitated twice in diethylether (200 mL). The solution was then decanted and volatiles removed *in-vacuo*, leaving a white powder identified as **2c** (1.34 g, 94%). ^{1}H NMR (400 MHz, DMSO-d6): δ = 2.60 (s, 3H, S−CH3), 3.29 (s, 3H), 3.79 (s, 3H), 4.19 (s, 3H), 4.24 (s, 3H). ^{13}C NMR (100 MHz, DMSO-d6): δ = 17.5 (s), 28.5 (s), 32.1 (s), 35.6 (s), 36.3 (s), 108.8 (s), 140.2 (s), 145.8 (s), 150.1 (s), 152.9 (s). Tdec = not obs.; Tg = not obs; Tm = 125.4-125.5 °C. EA calcd for $C_{10}H_{15}IN_4O_2S$: C, 32.67; H. 3.74; N, 13.86. Found: C, 32.80; H, 3.72; N, 13.79.

1,3,7,9-Tetramethyl-8-(methylthio)-2,6-dioxo-2,3,6,9-tetrahydropurinium Benzenesulfonate (**2d**): Compound S2 (0.50 g, 2.08 mmol) was added to acetonitrile (15 mL) and stirred for 15 minutes before adding methyl benzenesulfonate (1.27 g, 7.37 mmol). The solution was stirred for 24 hours in an oil bath at 50°C, concentrated by rotary evaporation, and precipitated in diethylether (200 mL). The solution was then decanted and volatiles removed *in-vacuo*, leaving a white powder identified as **2d** (0.74 g, 86 %). ^{1}H NMR (400 MHz, DMSO-d6): δ = 2.59 (s, 3H, S−CH3), 3.28 (s, 3H), 3.77 (s, 3H), 4.17 (s, 3H), 4.22 (s, 3H), 7.30 (m, 3H), 7.56 (m, 2H). ^{13}C NMR (100 MHz, DMSO-d6): δ = 17.4 (s), 28.4 (s), 31.8 (s), 35.4 (s), 36.3 (s), 108.5 (s), 124.9 (s), 127.6 (s), 128.4 (s), 139.8 (s), 145.5 (s), 147.7 (s), 149.8 (s), 152.8 (s). Tdec = not obs.; Tg = not obs; Tm = not obs. EA calcd for $C_{16}H_{20}N_4O_5S_2$: C, 46.59; H. 4.89; N, 13.58. Found: C, 46.65; H, 4.93; N, 13.46.

1,3,7,9-Tetramethyl-8-(methylthio)-2,6-dioxo-2,3,6,9-tetrahydropurinium Bis(trifluoromethylsulonyl)imide (**2e**): Compound **2a** (0.35 g, 0.91 mmol) was dissolved in deionized water (10 mL) followed by the dropwise addition of LiTFSI (0.27 g, 0.95 mmol) in deionized water (5 mL). The solution was stirred for 24 hours and the precipitate rinsed with deionized water (3x5 mL) and dried *in-vacuo* to isolate a white powder identified as **2e** (0.34 g, 69%). ^{1}H-NMR (400 MHz, DMSO-d6): δ = 2.59 (s, 3H, S-CH_3), 3.29 (s, 3H), 3.79 (s, 3H), 4.19 (s, 3H), 4.24 (s, 3H). ^{19}F-NMR (376 MHz, DMSO-d6): δ = -78.8 (s). ^{13}C-NMR (100 MHz, DMSO-d6): δ = 17.9 (s), 28.6 (s), 32.1 (s), 35.7 (s), 36.3 (s), 108.7 (s), 114.7 (s), 117.9 (s), 121.1 (s), 124.3 (s), 140.2 (s), 145.9 (s), 150.1 (s), 153.0 (s). T_{dec} = 269.1 °C; T_g = 75 °C; T_m = 143 °C. EA calcd. for $C_{12}H_{15}F_6N_5O_6S_3$: C, 26.92; H, 2.82; N, 13.08. Found: C, 27.09; H. 2.73; N, 13.05.

1,3,7,9-Tetramethyl-8-(methylthio)-2,6-dioxo-2,3,6,9-tetrahydropurinium Hexafluorophosphate (**2f**): Compound **2a** (0.50 g, 1.31 mmol) was dissolved in deionized water (10 mL) followed by the dropwise addition of KPF_6 (0.25 g, 1.37 mmol) in deionized water (5 mL). The solution was stirred for 24 hours and the precipitate rinsed with deionized water (3x5 mL) and dried *in-vacuo* to isolate a white powder identified as **2b** (0.32 g, 62%). ^{1}H-NMR (400 MHz, DMSO-d6): δ = 2.59 (s, 3H, S-CH_3), 3.29 (s, 3H), 3.78 (s, 3H), 4.19 (s, 3H), 4.23 (s, 3H). ^{19}F-NMR (376 MHz, DMSO-d6): δ = -69.7 (s), -71.6 (s) ^{13}C-NMR (100 MHz, DMSO-d6): δ = 17.6 (s), 28.7 (s), 32.2 (s), 35.8 (s), 36.5 (s), 109.1 (s), 140.4 (s), 145.9 (s), 150.3 (s), 153.2 (s). T_{dec} = not obs; T_g = not obs; T_m = not obs. EA calcd. for $C_{10}H_{15}F_6N_4O_2PS$: C, 30.01; H, 3.78; N, 14.00. Found: C, 30.09 H. 3.82; N, 13.93.

Results and Discussion

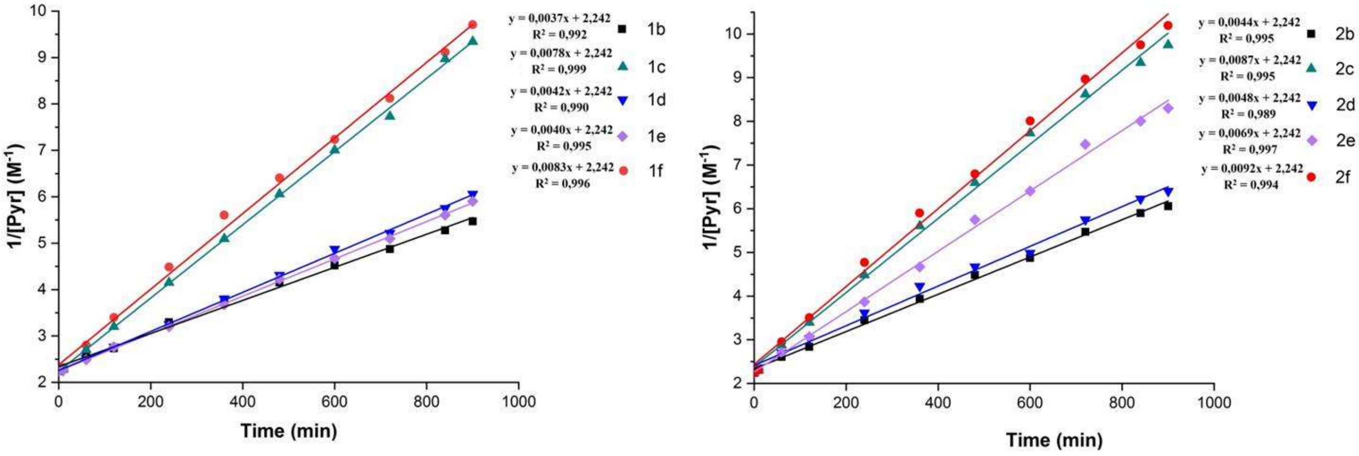

Figure S1: Second-order plots of kinetic data with their R^2 values and linear regression equation. A 1:1 molar ratio of alkylating agent (compounds **1** and **2**, with their counter anions) and pyridine (0.1851 mmol) were dissolved in DMSO-d6 (0.4 mL) and added to a standard NMR tube (reactant concentration was 0.45 M). The sample was then heated to reaction temperature (90 °C) and analyzed in-situ every hour by ^{1}H-NMR spectroscopy over 15 h.

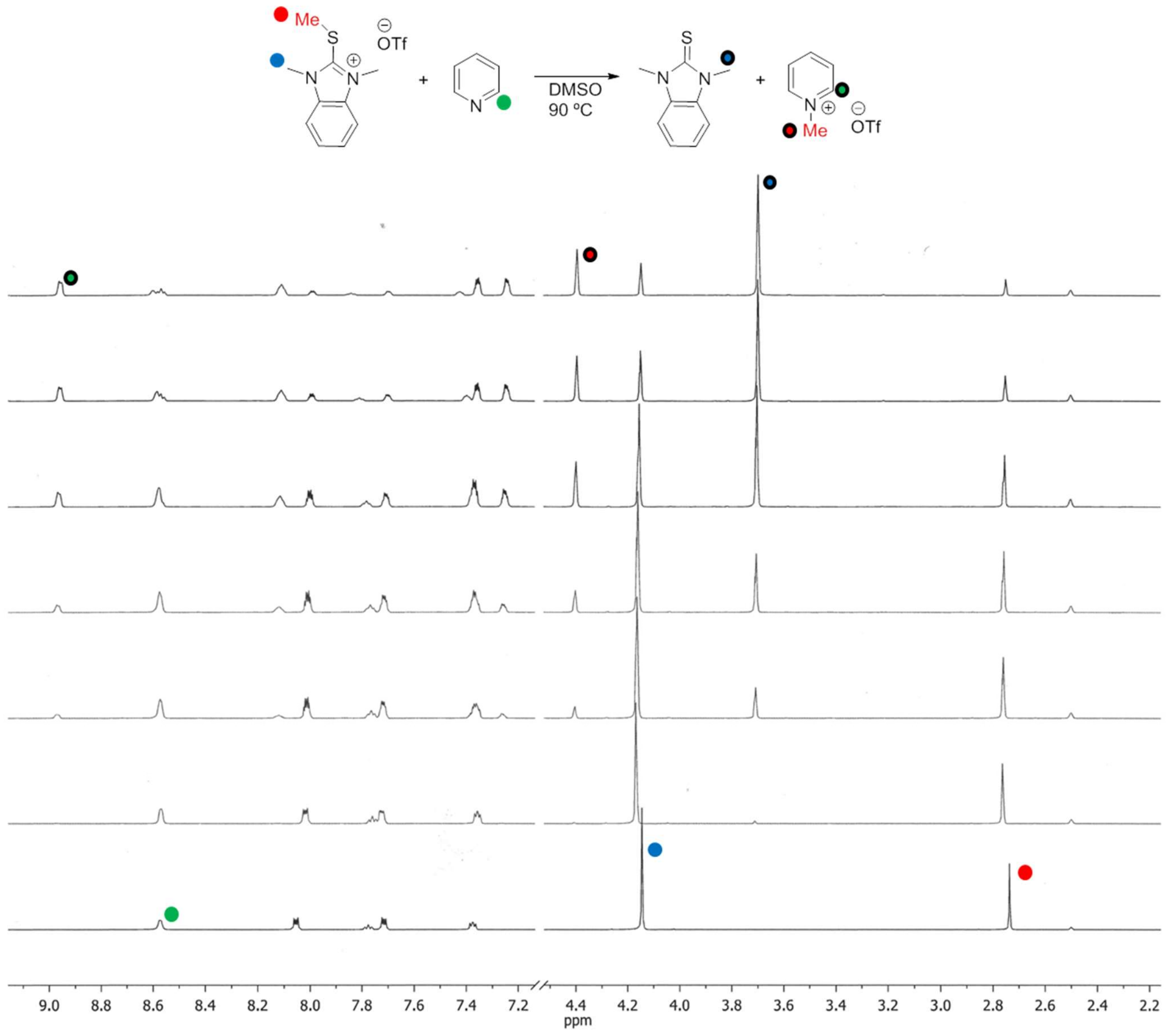

Figure S2: Stack plot of the reaction between **1c** and pyridine in DMSO-d6 at 90 ºC.

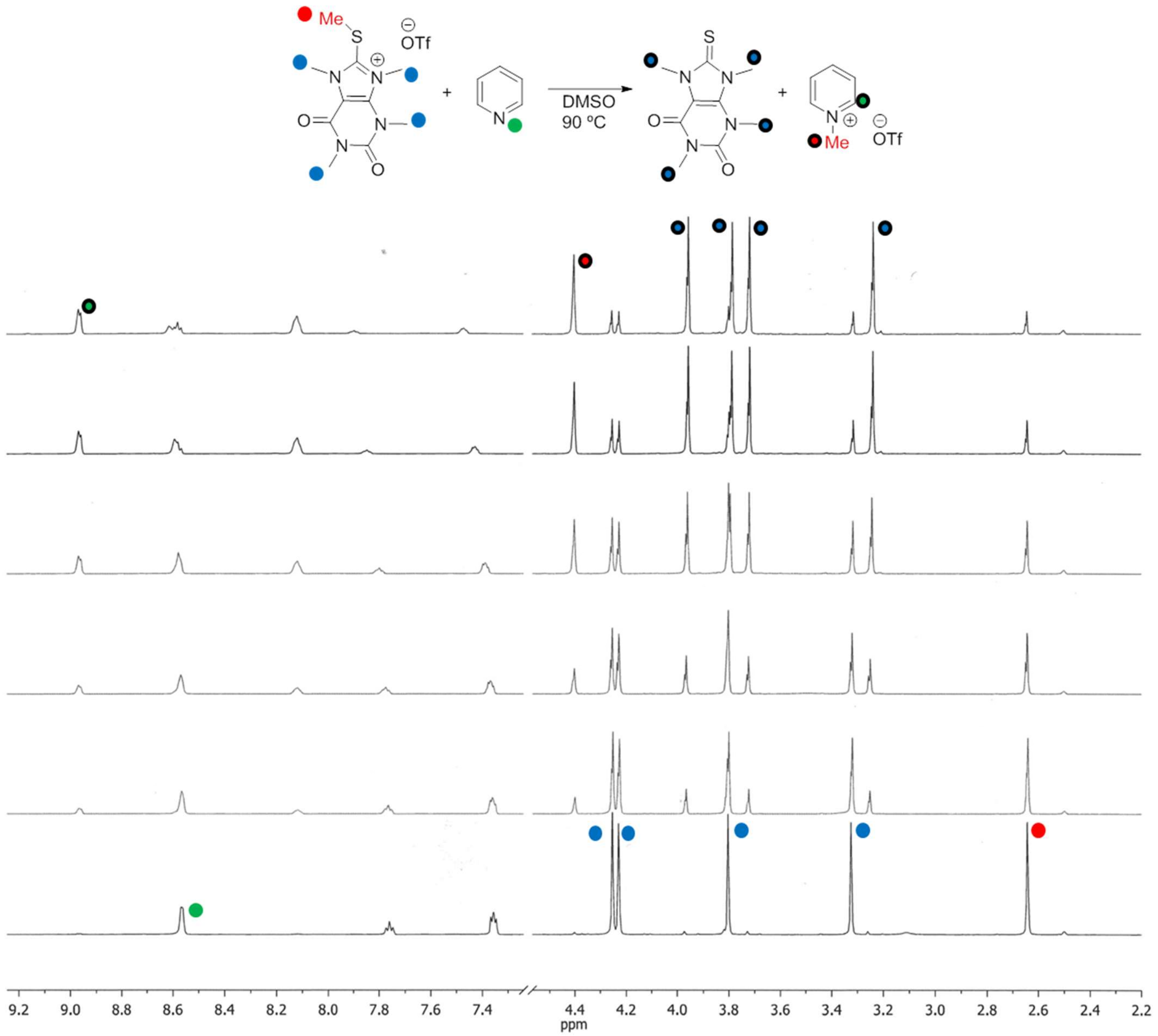

Figure S3: Stack plot of the reaction between **2c** and pyridine in DMSO-d6 at 90 ºC.

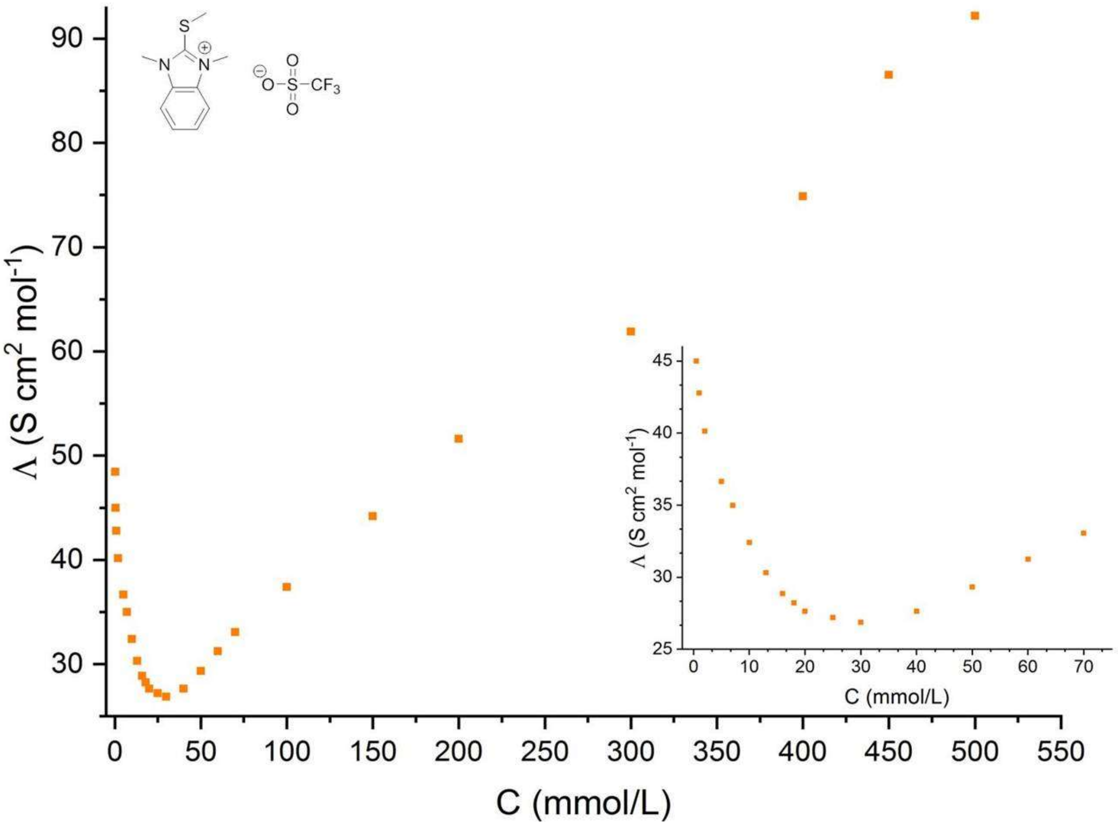

Figure S4: Conductivity of **1c** in DMSO.

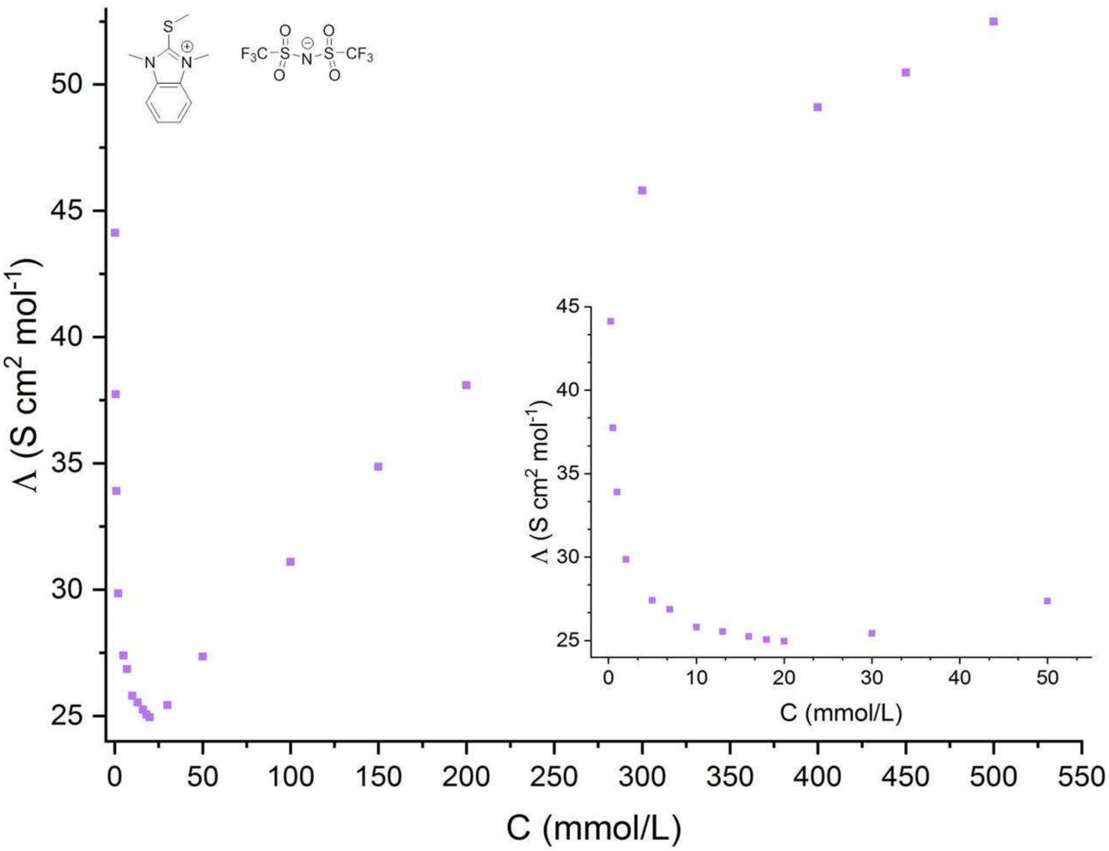

Figure S5: Conductivity of **1e** in DMSO.

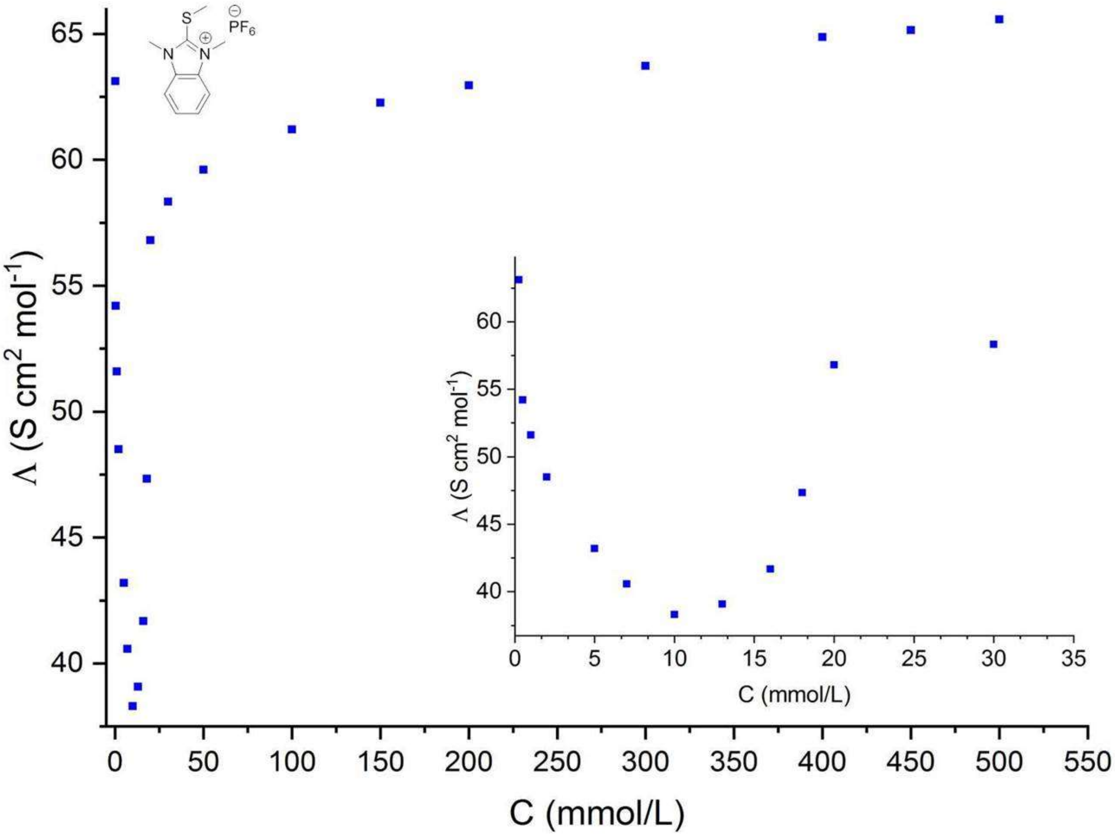

Figure S6: Conductivity of **1b** in DMSO.

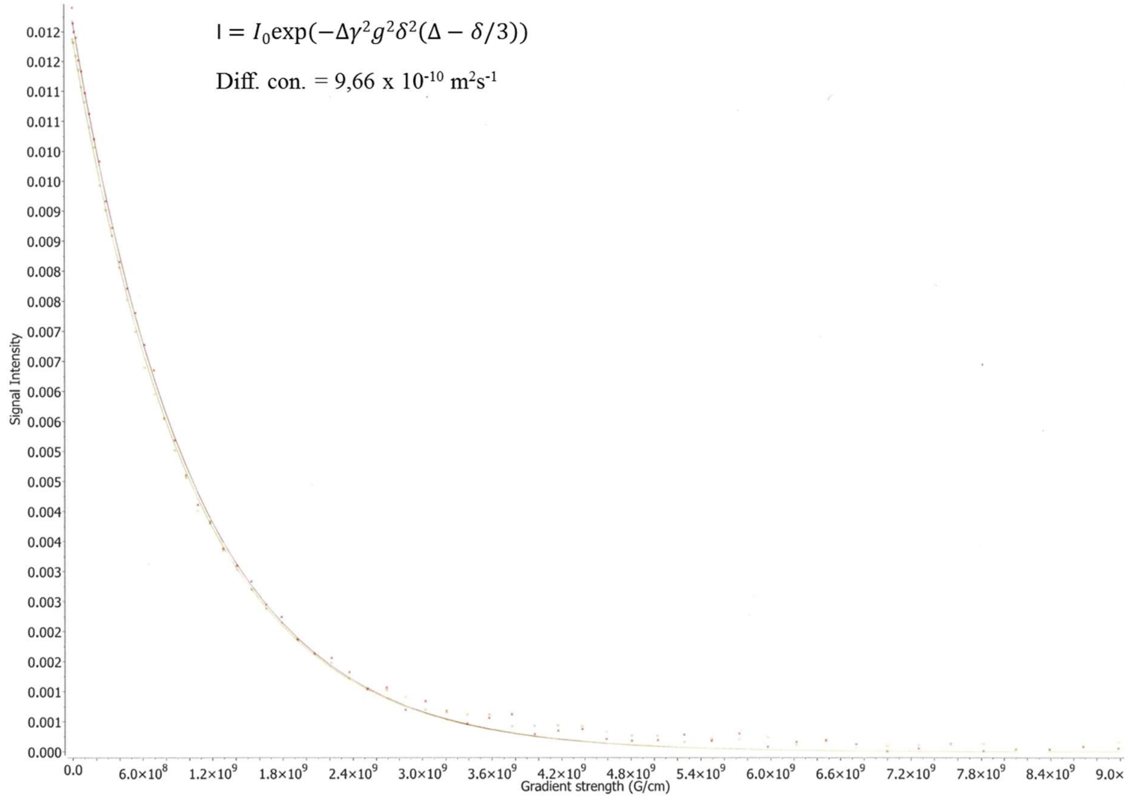

Figure S7: Gaussian fit of **1b** in DMSO-d6 at the concentration of 10 mM.

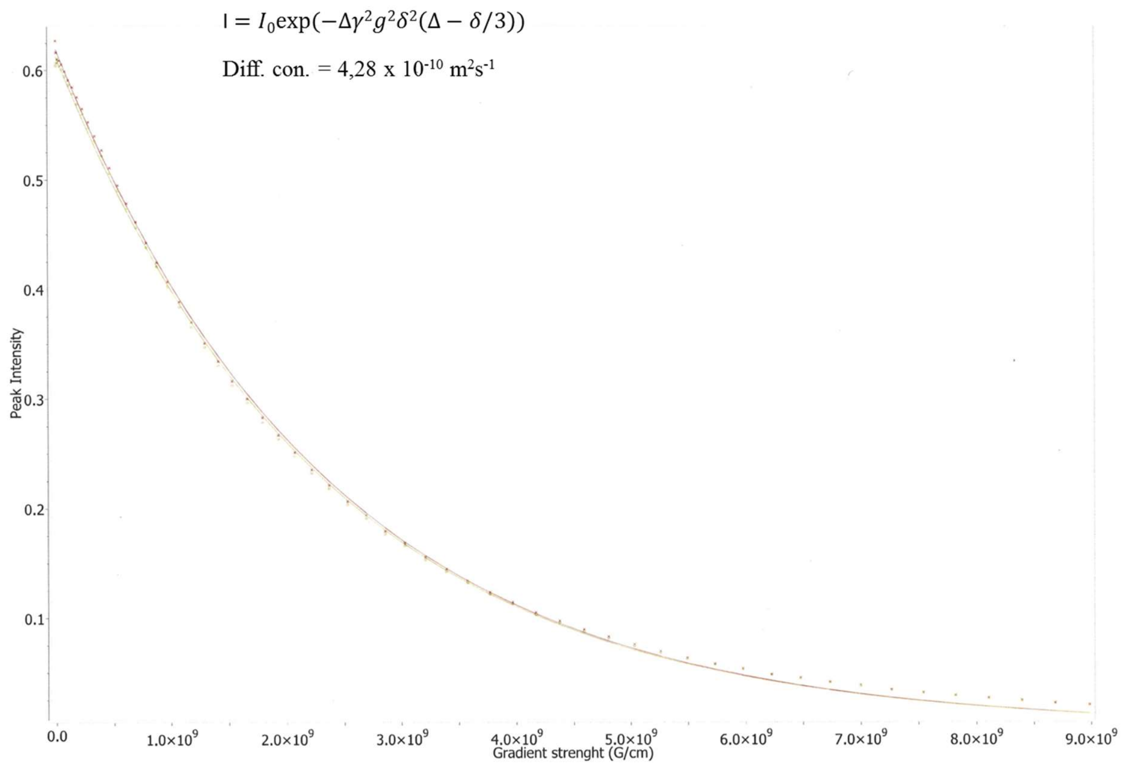

Figure S8: Gaussian fit of **1b** in DMSO-d6 at the concentration of 462 mM.

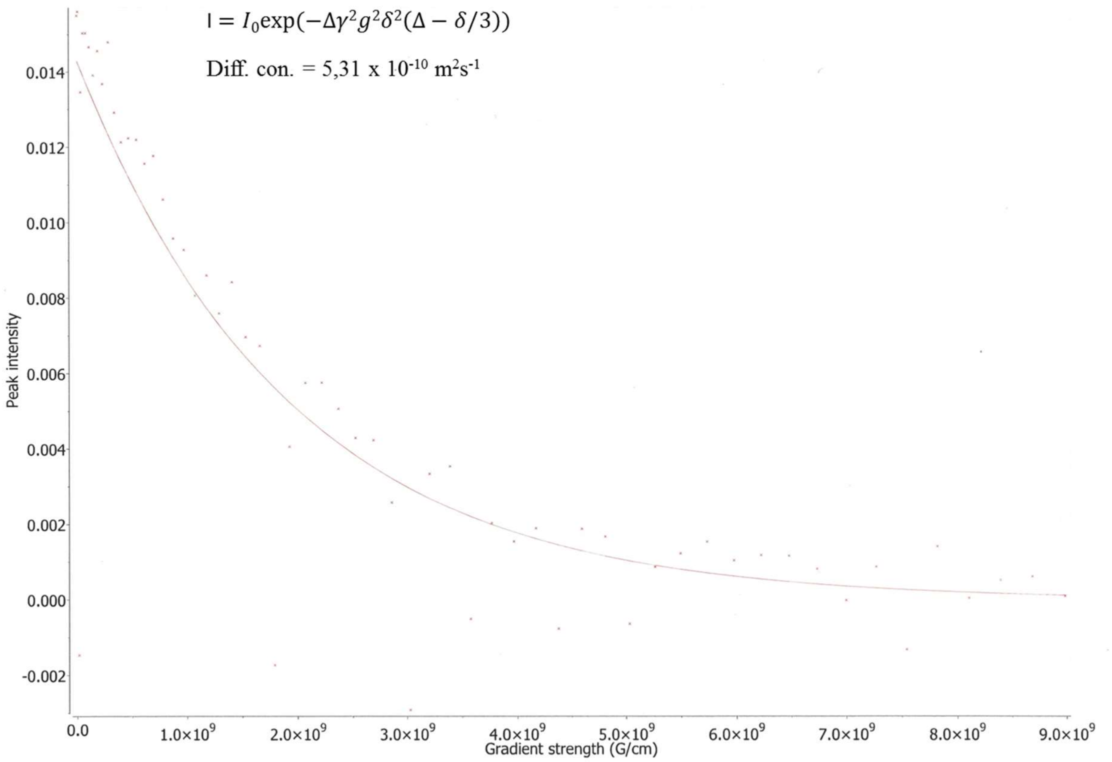

Figure S9: Gaussian fit of **1c** in DMSO-d6 at the concentration of 30 mM.

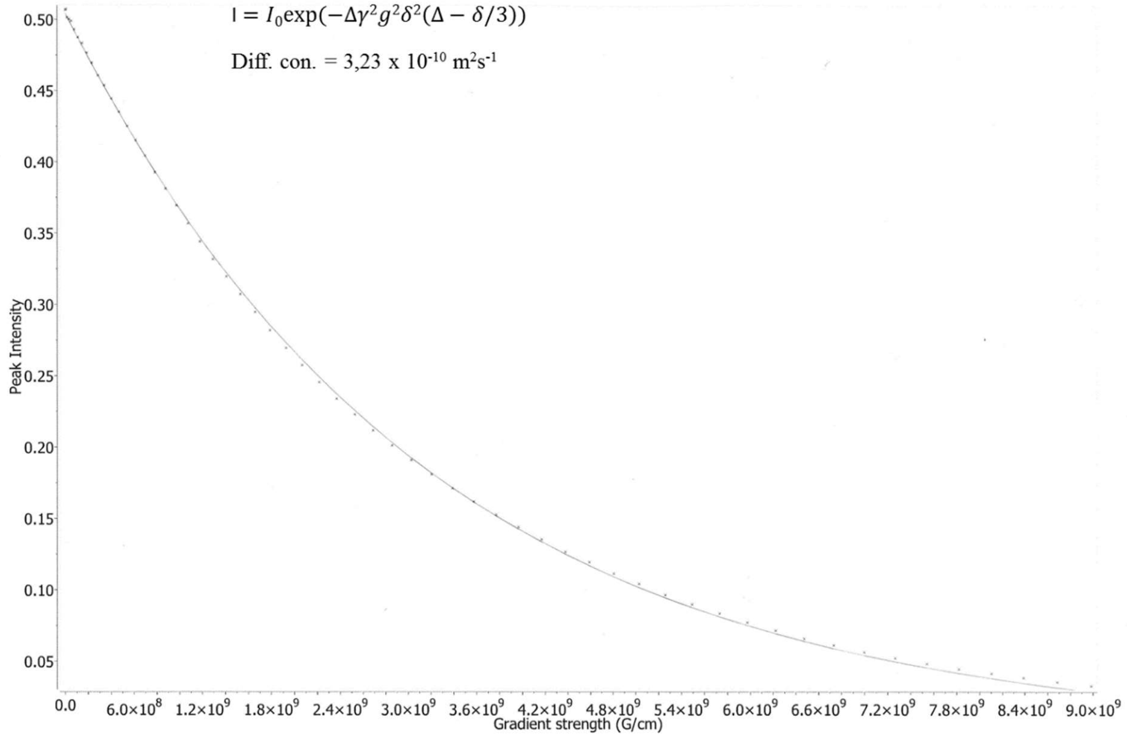

Figure S10: Gaussian fit of **1c** in DMSO-d6 at the concentration of 462 mM.

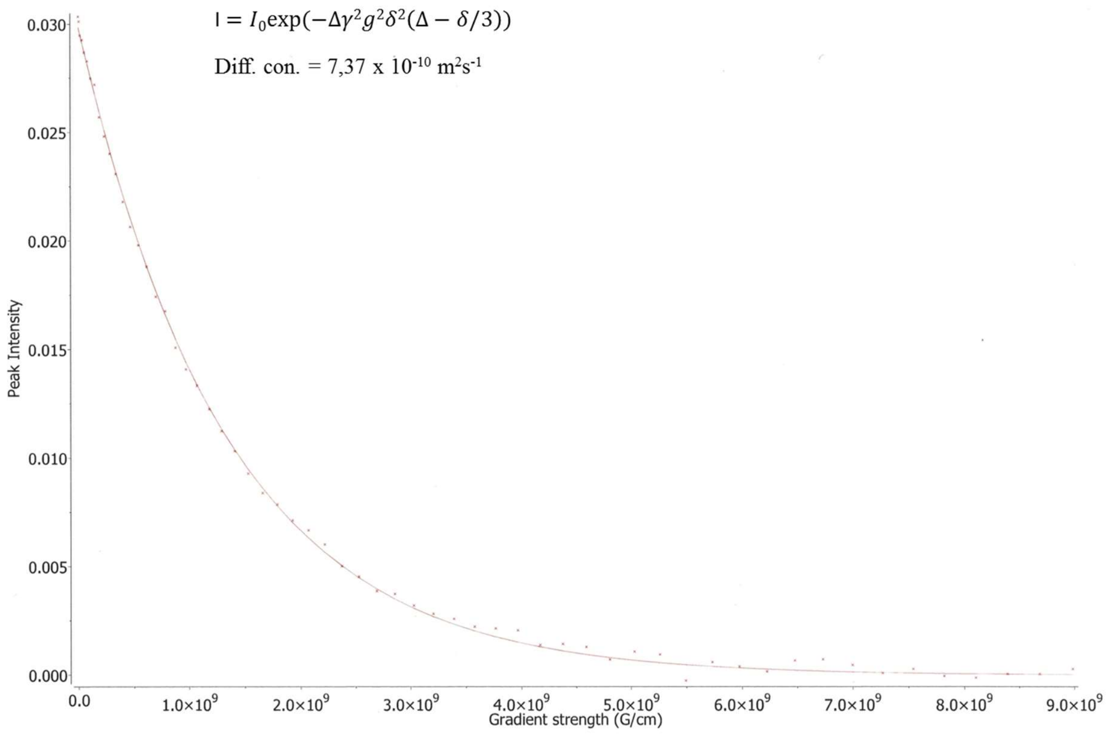

Figure S11: Gaussian fit of **1e** in DMSO-d6 at the concentration of 20 mM.

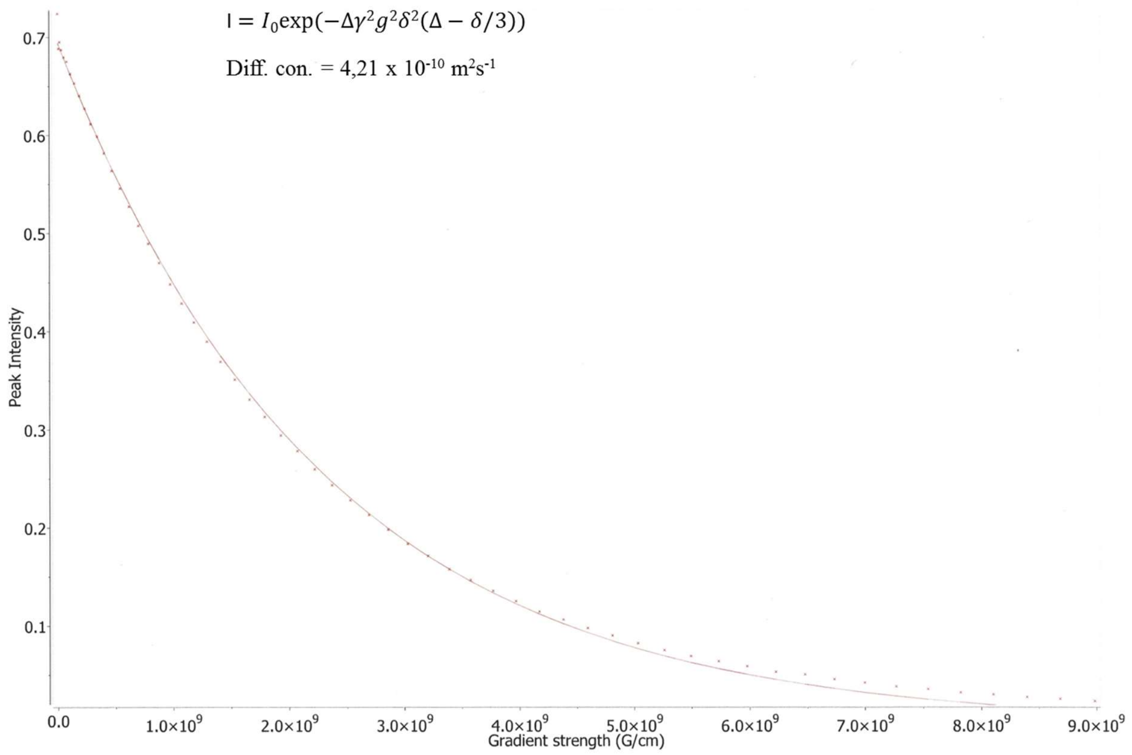

Figure S12: Gaussian fit of **1b** in DMSO-d6 at the concentration of 462 mM.

Table S1: Diffusion coefficients and hydrodynamic radii for compounds 1c, 1e, 1f at the concentration of their minima of molar conductivity.

Compound	Diffusion coeff. Cation (m^2s^{-1})	Diffusion coeff. Anion (m^2s^{-1})	Reference diffusion coeff. Anion (m^2s^{-1})	Rh cation (Å)	Rh anion (Å)
**1c**	2,85 x 10^{-10}	5,31 x 10^{-10}	0,48 x 10^{-7}	3,97	2,08
**1e**	5,23 x 10^{-10}	7,37 x 10^{-10}	1,70 x 10^{-8}	2,11	1,50
**1f**	5,95 x 10^{-10}	9,66 x 10^{-10}	1,15 x 10^{-8}	1,86	1,14

Table S2: Diffusion coefficients and hydrodynamic radii for compounds at 462 mM.

Compound	Diffusion coeff. Cation (m^2s^{-1})	Diffusion coeff. Anion (m^2s^{-1})	Reference diffusion coeff. Anion (m^2s^{-1})	Rh cation (Å)	Rh anion (Å)
**1c**	2,17 x 10^{-10}	3,23 x 10^{-10}	0,48 x 10^{-7}	5,08	3,42
**1e**	2,26 x 10^{-10}	4,21 x 10^{-10}	1,70 x 10^{-8}	4,89	2,62
**1f**	1,95 x 10^{-10}	4,28 x 10^{-10}	1,15 x 10^{-8}	5,65	2,58

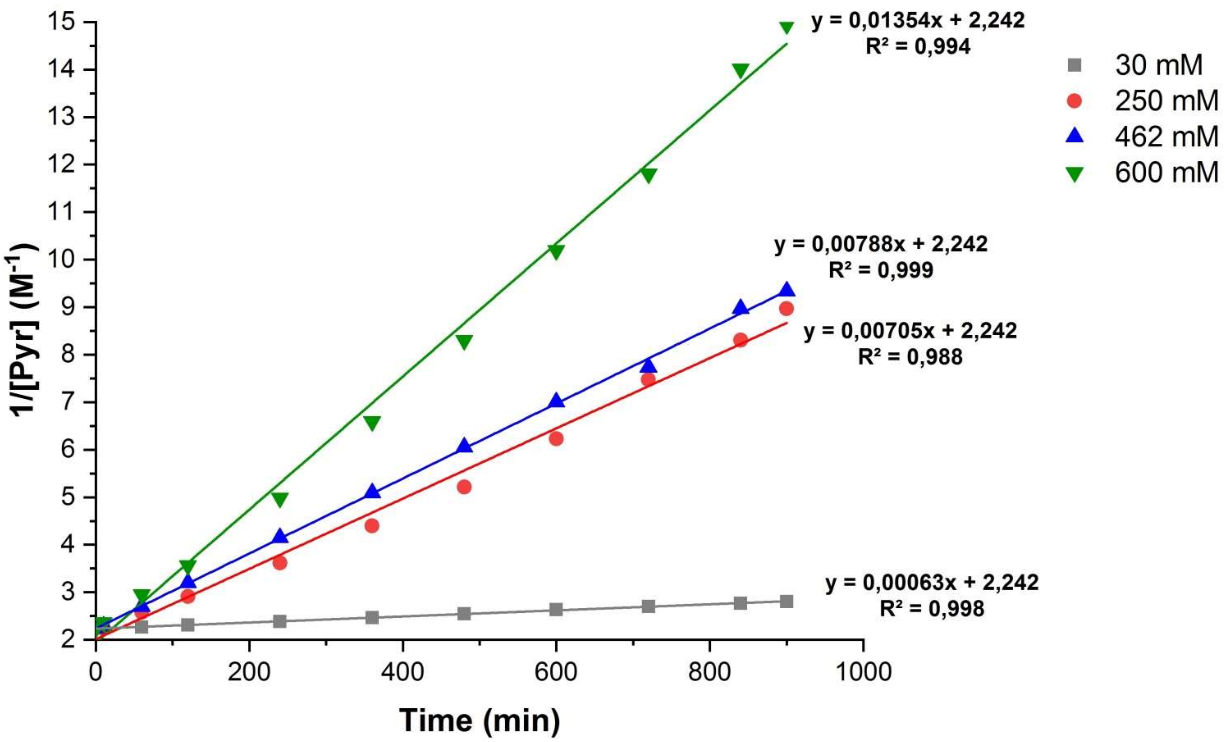

Figure S13: Second-order plots of kinetic data with their R^2 values and linear regression equation for 1c at different concentrations. A 1:1 molar ratio of alkylating agent and pyridine were dissolved in DMSO-d6 (0.4 mL) and added to a standard NMR tube. The sample was then heated to reaction temperature (90 °C) and analyzed in-situ every hour by ^{1}H-NMR spectroscopy over 15 h.

Computational Methods

A synergistic molecular mechanics MM and the advanced quantum mechanical calculations of counterion coordinated complexes were carried out using Desmond and Jaguar programs, as implemented in Schrödinger Material Suite 2019-1[2] as well as Gaussian software. The MM based conformational search was performed on the geometry of the counterion coordinated pre-complexes applying the Mixed Torsional/Low-mode (MT/LMOD) algorithm of MacroModel to obtain insight into the different binding modes of the different counterions, cation and pyridine. The root mean square deviation (RMSD) cutoff to eliminate redundant conformers was adjusted to 0.25 Å and further minimized using the OPLS-2005 force field, with a truncated Newton conjugated gradient (TNCG) method. Top unique conformers were chosen and their relative energies were calculated. Then, the lowest energy conformers were further optimized applying density functional theory (DFT) calculations.

The DFT based geometry optimizations of all the transition states, precomplexes, intermediates, starting materials and products were carried out using the range-separated, dispersion corrected ωB97X-D with the combined 6-311G(d,p)[3,4] basis set for the all atoms and SDD for iodine atom (Gen) and the integrated equation formalism polarized continuum solvation model (IEFPCM)[5]; applying the default solvent parameters for DMSO) using Gaussian 09[6] package on Compute Canada. Themethod choose based on balancing both computational time and accuracy.

The optimized geometries were confirmed by frequency calculations as minima (zero imaginary frequencies) or transition states (one imaginary frequency). The thermochemical quantities were analyzed at 363 K (the experimental temperature), and the IRC was used to obtain the minima of products and the starting materials on either side of each transition state.

The interaction energy were calculated at the second order Moller–Plessett perturbation theory (MP2)/6-311G(3df,2p)[7,8] level as well as the range-separated, dispersion corrected ωB97X-D/6-311G(d,p)/Lanl2dz [9]method on the ωB97X-D /6-311G(d,p) optimized geometries. These levels of theory were reported as one of the reliable theory for analyzing the interaction energetic trends on ILs.

For the iodine and PF_6^-, the single-point calculations were performed at the range-separated, dispersion corrected ωB97X-D/aug-cc-pVTZ [9] method using the aug-cc-pVTZ basis set for the all atoms and SDD for iodine on the ωB97X-D /6-311G(d,p) optimized geometries. The structural visualizations were performed using both GaussView v5.0.8.4 as well as Maestro 2019-1. To analyze in detail the strength and nature of the important inter- interactions and their topological density characteristics, detailed QTAIM analysis was performed. The topological analysis of the electron density was performed with Bader's quantum theory of atoms in molecules (QTAIM) using the AIM2000 software.[5]

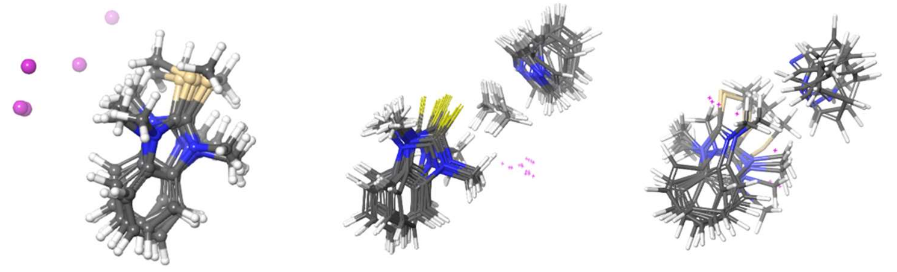

Figure S14. The overlaid conformers within 4-6 kcal/mol of the global minimum on the iodide coordinated pre-complexes and the combination of a single ion pair and pyridine.

Table S3. Comparison of the computed activation energies of counterion mediated one-step versus two-step path.

*The value in the bracket is the calculated values at ωB97X-D/aug-cc-pVTZ [9] method using the aug-cc-pVTZ basis set for the all atoms and SDD for iodine.

	**ωB97X-D/6-311G**<br>**scrf=(iefpcm,dmso)**<br>**ΔG‡ (kcal/mol)**		
I^-*<br>**TS1a**$_{(1Step)}$<br>**TS1a**$_{(2step)}$	30.5 (31.3)<br>23.9 (27.9)	TFSI<br>**TS1e**$_{(1Step)}$<br>**TS1e**$_{(2step)}$	29.9<br>27.4
PF_6^-<br>**TS1b**$_{(1Step)}$<br>**TS1b**$_{(2step)}$	29.2 (32.0)<br>44.7 (46.4)	$CH_3SO_3^-$<br>**TS1f**$_{(1Step)}$<br>**TS1f+**$_{(2step)}$	32.2<br>44.4
$CF_3SO_3^-$<br>**TS1c**$_{(1Step)}$<br>**TS1c**$_{(2step)}$	30.2<br>32.8		

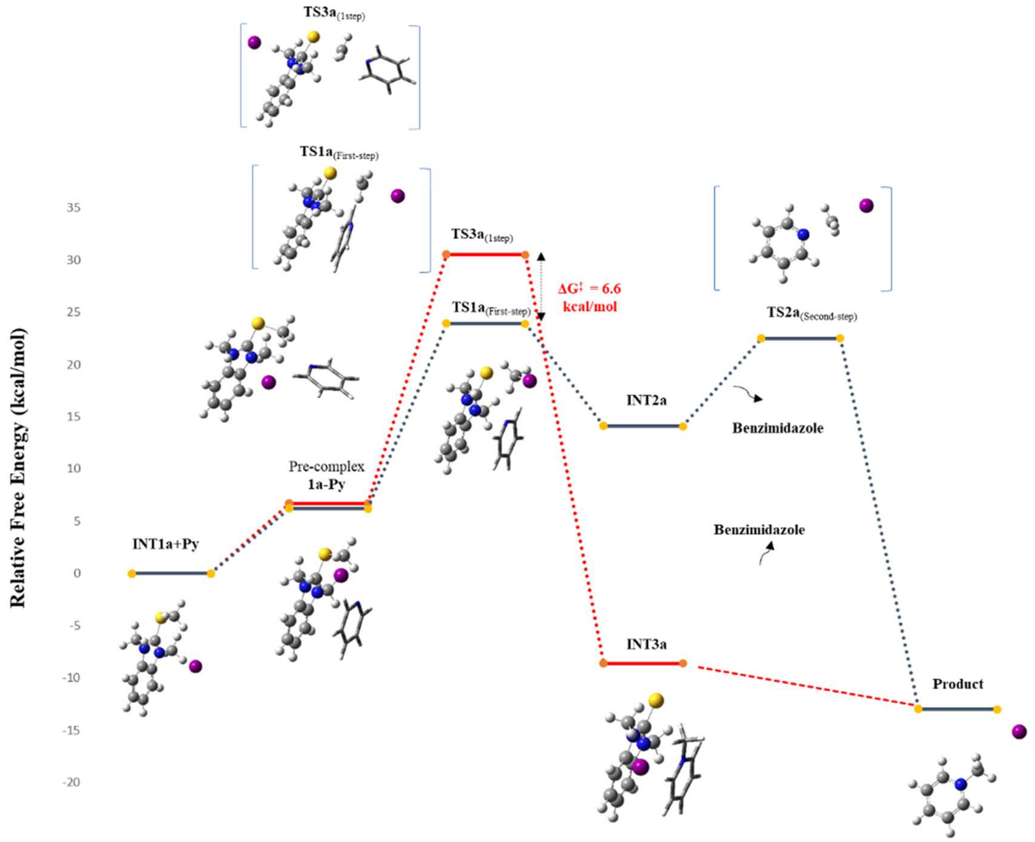

TS3a(1step)
TS1a(First-step)
Relative Free Energy (kcal/mol)
35
30
25
20
15
10
5
0
-5
-10
-15
-20
TS3a(1step)
ΔG‡ = 6.6 kcal/mol
TS1a(First-step)
TS2a(Second-step)
INT2a
Benzimidazole
Pre-complex
1a-Py
INT1a+Py
Benzimidazole
INT3a
Product

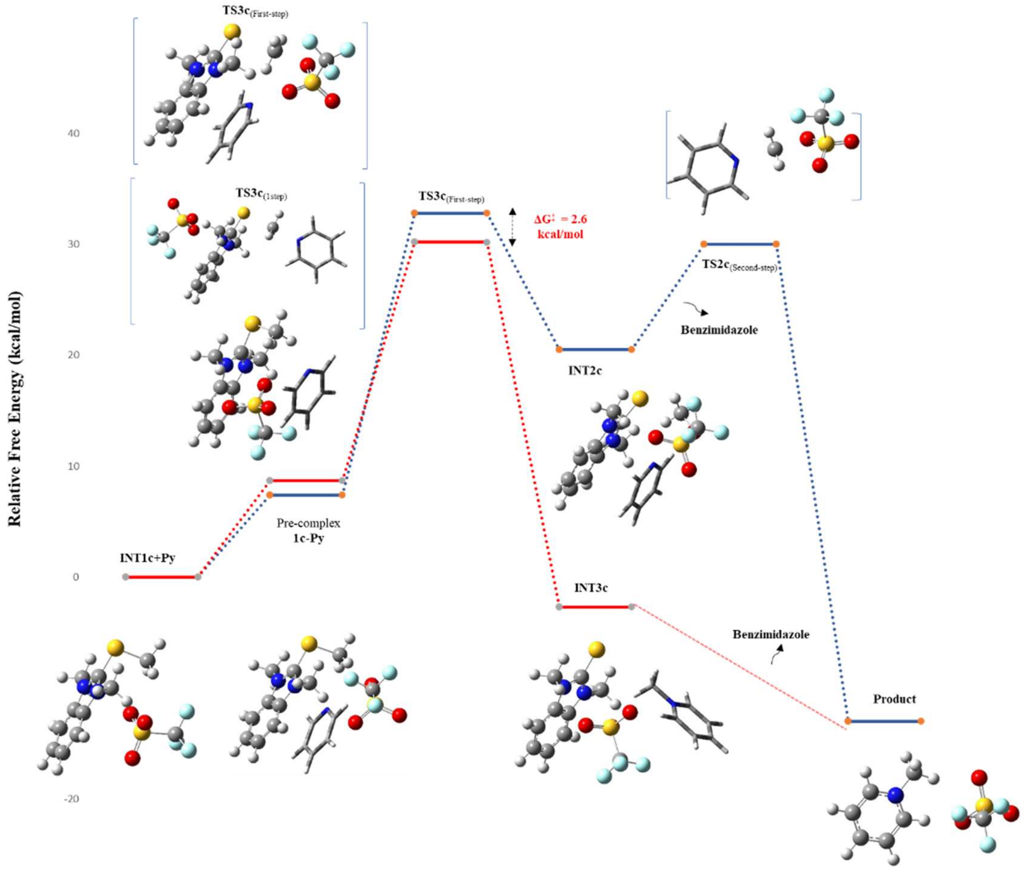

TS3c(First-step)
TS3c(1step)
Relative Free Energy (kcal/mol)
40
30
20
10
0
-20
TS3c(First-step)
ΔG‡ = 2.6 kcal/mol
TS2c(Second-step)
INT2c
Benzimidazole
Pre-complex
1c-Py
INT1c+Py
INT3c
Benzimidazole
Product

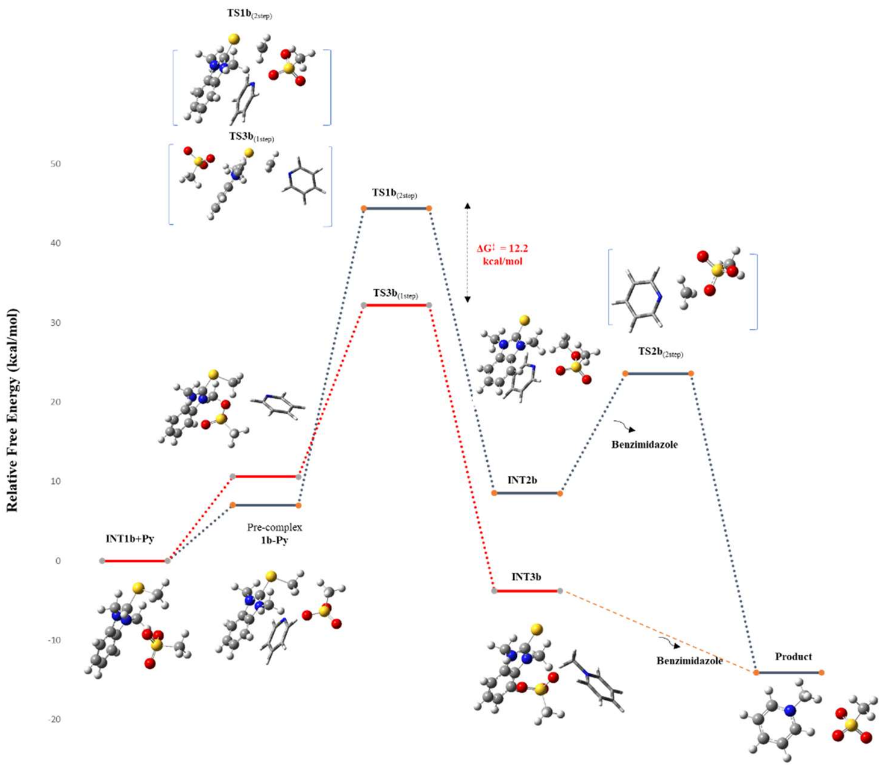

Figure S15. ωB97X-D/6-311G(calculated reaction pathways [competing two steps (red path) and concerted path (blue path)] of the (a) iodide, (b) $CF_3SO_3^-$, and (c) $CH_3SO_3^-$ mediator and/or competing alkylation reaction at 363 K, and IFPCM solvation model (using DMSO).

Table S4. Calculated activation energies for the 1step versus 2step transition states of anion promoting alkylation processes.

	**ωB97X-D/6-311G**<br>$\Delta G^{\ddagger}$ (kcal/mol)**		**ωB97X-D/6-311G**<br>$\Delta G^{\ddagger}$ (kcal/mol)**
Addition Mode Iodide<br>**TS1a**$_{(2Step)}$<br>**TS1a**$_{(1Step)}$	23.9<br>30.5<br>**$\Delta\Delta G^{\ddagger}$ = 6.6**	-	-
Addition Mode $CH_3SO_3^-$<br>**TS1b**$_{(2Step)}$<br>**TS1b**$_{(1Step)}$	44.4<br>32.2<br>**$\Delta\Delta G^{\ddagger}$ = 12.2**	Addition Mode $TFSI^-$<br>**TS1e**$_{(2Step)}$<br>**TS1e**$_{(1Step)in}$	27.4<br>29.9<br>**$\Delta\Delta G^{\ddagger}$ = 2.5**
Addition Mode $CF_3SO_3^-$<br>**TS1c**$_{(2Step)}$<br>**TS1c**$_{(1Step)}$	32.8<br>30.2<br>**$\Delta\Delta G^{\ddagger}$ = 2.6**	Addition Mode PF_6^-<br>**TS1b**$_{(2Step)}$<br>**TS1b**$_{(1Step)}$	44.7<br>29.2<br>$\Delta\Delta G^{\ddagger}$ = 15.5

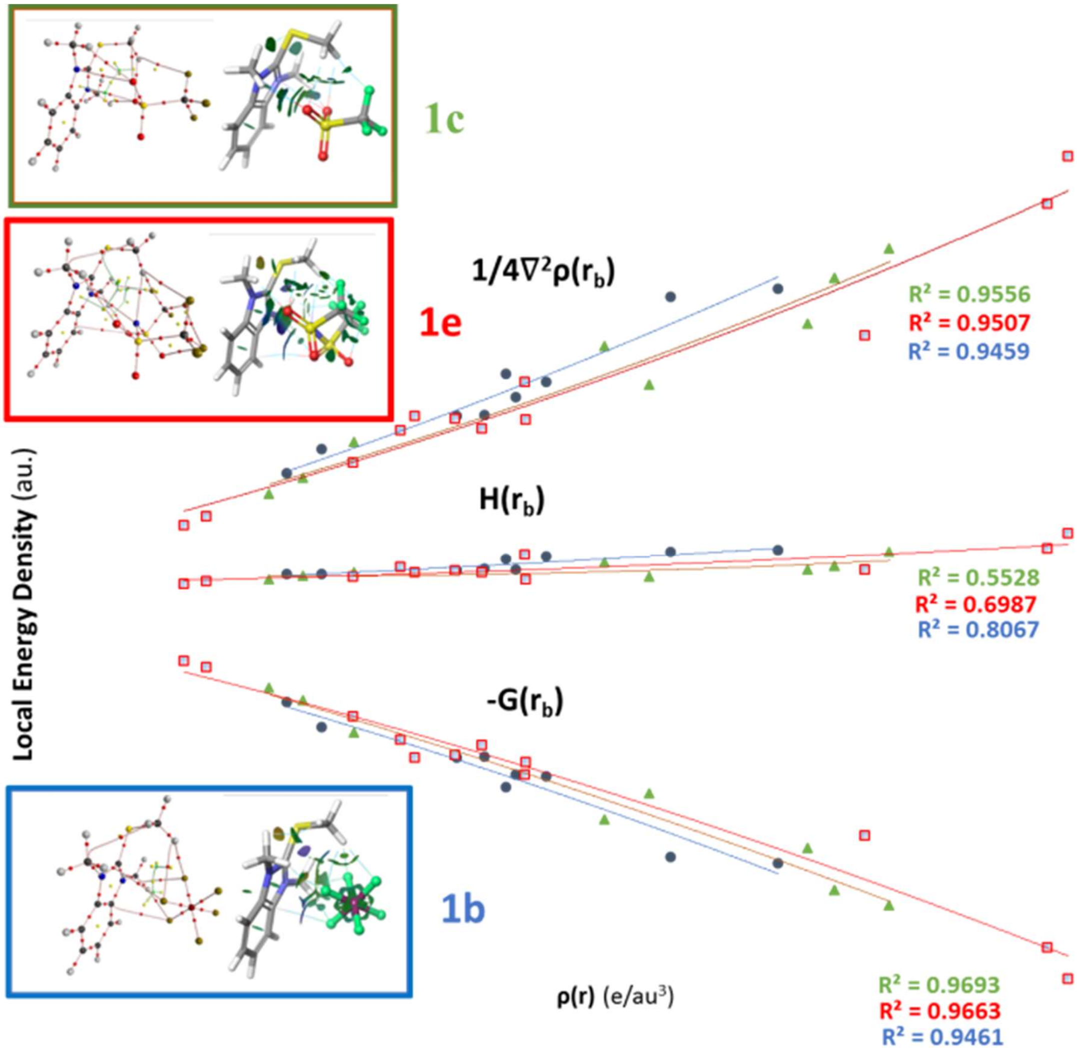

Figure S16. Variation of $1/4\nabla^2\rho(r_b)$,($-G(r_b)$) and the $H(r_b)$ energy densities as a function of bond strength of a) [bmim][CF_3SO_3], b) [bmim][NTf_2], and c) [bmim][PF_6].The correlation coefficients, R, as well as the linear regressions equations are depicted.

Table S5. The predicted ion pair binding energy, E_b, and the electron density characteristics, Ex and $\rho(r_b)$.

	MP2/6-311+G(3df,2p) E_b (kcal/mol)	ωB97X-D/6-311G(d,p) E_b (kcal/mol)	E_{MP2} (kcal/mol)	Ex (kcal/mol)	$\rho_{total}(r_b)$ (e/au)
**TS1c**	-13.1	-12.1	-4.0	-12.7	0.1292
**TS1e**	-17.9	-14.0	-5.6	-14.0	0.07145
**TS1b**	-10.7	-9.7	-3.9	-9.9	0.04898

A

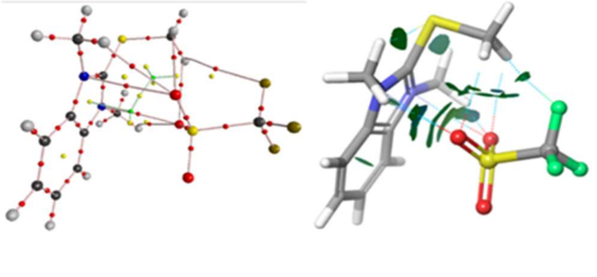

$CF3SO3^-_{(in)}$	$\rho(r)$ (e/au^3)	$\nabla^2\rho(r)$ (e/au^5)	$G(r)$ (e/au^3)	$H(r)$ (e/au^5)	E(x) (kcal/mol)	$-G(r)/V(r)$ (e/au^5)
$O_{(X^-)}...MeN_{(Bz)}$	0.0753	0.0304	0.0064	0.0012	-1.7	1.22
$O_{(X^-)}...N_{(Bz)}$	0.0106	0.0384	0.0085	0.0011	-2.3	1.14
$O_{(X^-)}...MeN_{(Bz)}$	0.0113	0.0419	0.0090	0.0015	-2.3	1.20
$O_{(X^-)}...N_{(Bz)}$	0.0106	0.0384	0.0085	0.0011	-2.3	1.14
$O_{(X^-)}...SMe_{(Bz)}$	0.0081	0.0258	0.0057	0.0008	-1.5	1.15
$O_{(X^-)}...SMe_{(Bz)}$	0.0102	0.0330	0.0073	0.0010	-2.0	1.15
$F_{(X^-)}...SMe_{(Bz)}$	0.00307	0.0129	0.0025	0.0007	-0.6	1.36
**Total**	**0.12917**					
(3,+3)CCP	0.00353	0.0148	0.0029	0.0008	-0.7	1.37
(3,+3)CCP	0.00420	0.0190	0.0039	0.0009	-0.9	1.29

B

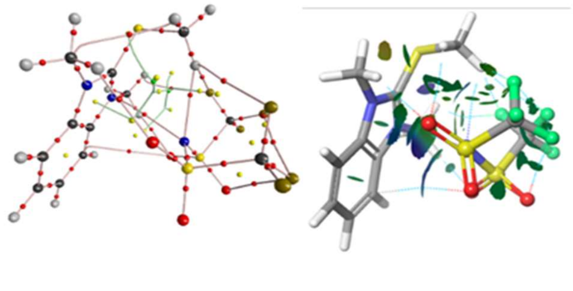

$TFSI_{in}$	$\rho(r)$ (e/au^3)	$\nabla^2\rho(r)$ (e/au^5)	$G(r)$ (e/au^3)	$H(r)$ (e/au^5)	E(x) (kcal/mol)	$-G(r)/V(r)$ (e/au^5)
$O_{(X^-)}...C_{Ar(Bz)}$	0.00589	0.0206	0.00425	0.0010	-1.1	1.27
$O_{(X^-)}...MeN_{(Bz)}$	0.0134	0.0473	0.0102	0.0016	-2.7	1.18
$F_{(X^-)}...MeN_{(Bz)}$	0.00481	0.0204	0.00407	0.0010	-1.0	1.34
$N_{(X^-)}...N_{(Bz)}$	0.00648	0.0216	0.00475	0.0007	-1.3	1.16
$O_{(X^-)}...MeN_{(Bz)}$	0.0137	0.0528	0.0112	0.0020	-2.9	1.22
$F_{(X^-)}...SMe_{(Bz)}$	0.00418	0.0166	0.0034	0.0008	-0.8	1.28
$F_{(X^-)}...SMe_{(Bz)}$	0.00553	0.0218	0.00452	0.0009	-1.1	1.26
$F_{(X^-)}...SMe_{(Bz)}$	0.0110	0.0316	0.00693	0.0010	-1.9	1.16
$MeN_{(Bz)}...SMe_{(Bz)}$	0.00646	0.0261	0.00512	0.0014	-1.2	1.38
**Total**	**0.07145**					
(3,+3)	0.00193	0.0092	0.00176	0.0005	-0.4	1.44
(3,+3)	0.00500	0.0220	0.00462	0.0010	-1.2	1.24
(3,+3)	0.00223	0.0102	0.00194	0.0006	-0.4	1.47

C

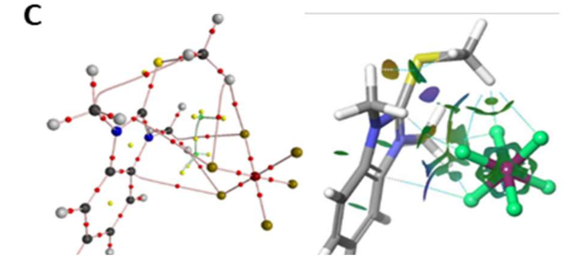

$PF_6^-{}_{in}$	$\rho(r)$ (e/au^3)	$\nabla^2\rho(r)$ (e/au^5)	$G(r)$ (e/au^3)	$H(r)$ (e/au^5)	E(x) (kcal/mol)	$-G(r)/V(r)$ (e/au^5)
$F_{(X^-)}...C_{Ar(Bz)}$	0.00593	0.0222	0.00457	0.0010	-1.1	1.27
$F_{(X^-)}...C_{Ar(Bz)}$	0.00839	0.0362	0.00754	0.0015	-1.9	1.25
$F_{(X^-)}...SMe_{(Bz)}$	0.00555	0.0221	0.00461	0.0010	-1.2	1.25
$F_{(X^-)}...SMe_{(Bz)}$	0.00982	0.0372	0.00775	0.0015	-1.9	1.25
$O_{(X^-)}...MeN_{(Bz)}$	0.00634	0.0244	0.00512	0.0010	-1.3	1.23
$F_{(X^-)}...C_{Ar(Bz)}$	0.00621	0.0270	0.00548	0.0013	-1.3	1.31
$MeN_{(Bz)}...SMe_{(Bz)}$	0.00674	0.0261	0.00517	0.0014	-1.2	1.3
**Total**	**0.04898**					

Figure S17. The distribution of bond, ring and cage critical points in the inner cavity of a) [bmim][CF_3SO_3], b) [bmim][TFSI], c) [bmim][PF_6] using QTAIM and non-covalent isosurfaces by NCI as well as the detailed topological parameters.

Table S6. Variation of $1/4\nabla^2\rho(r_b)$,$(-G(r_b))$ and the $H(r_b)$ energy densities as a function of bond strength of [bmim][CF_3SO_3], [bmim][NTf_2], and [bmim][PF_6]. The correlation coefficients, R, as well as the polynomial regressions equations are depicted.

	**Polynomial Equation of $1/4\nabla^2\rho(r_b)$ (R^2)**	**Polynomial Equation of $H(r_b)$ (R^2)**	**Polynomial Equation of $-G(r_b)$ (R^2)**
**1c**	$y = 19.051x^2 + 0.5272x + 0.0017$ (0.9556)	$y = 7.166x^2 - 0.0464x + 0.0009$ (0.5528)	$y = -11.885x^2 - 0.5736x - 0.0009$ (0.9693)
**1e**	$y = 17.054x^2 + 0.5431x + 0.0016$ (0.9507)	$y = 3.2923x^2 + 0.0402x + 0.0006$ (0.6987)	$y = -13.762x^2 - 0.5029x – 0.0010$ (0.9663)
**1b**	$y = 18.544x^2 + 0.6412x + 0.0016$ (0.9459)	$y = 2.9233x^2 + 0.0878x + 0.0005$ (0.8087)	$y = -15.621x^2 - 0.5534x - 0.0011$ (0.9507)

Selected DFT calculated transition state, complex geometries & thermochemical data

1. benzimidazolium-PF_6

 # opt=calcfc freq=noraman wb97xd/6-311g(d,p) scrf=(iefpcm,solvent=dmso

) geom=connectivity temperature=363

Zero-point correction= 0.238443 (Hartree/Particle)

 Thermal correction to Energy= 0.267654

 Thermal correction to Enthalpy= 0.268804

 Thermal correction to Gibbs Free Energy= 0.170819

 Sum of electronic and zero-point Energies= -1836.955080

 Sum of electronic and thermal Energies= -1836.925869

 Sum of electronic and thermal Enthalpies= -1836.924719

 Sum of electronic and thermal Free Energies= -1837.022704

Title Card Required

0 1

C	1.86824500	0.93523300	-0.56361200
C	2.29508300	2.02981000	-1.31048600
C	1.49437900	2.28836100	1.42246100
C	1.91698900	3.37719600	0.68346100
C	2.31083400	3.24997000	-0.66088000
H	2.63334300	4.13198700	-1.20017100
H	1.18163000	2.38633800	2.45354300
H	1.94376200	4.35427400	1.14961900
H	2.59688100	1.93167100	-2.34501800
C	0.62301400	-0.49364700	2.53579900
H	1.33168500	-0.11135500	3.26953900
H	-0.34809900	-0.01597100	2.65328800

H	0.52171000	-1.56919800	2.64743700
C	1.90753600	-0.94016100	-2.23331200
H	2.97115200	-1.07536500	-2.42986200
H	1.38697700	-1.89198800	-2.30811200
H	1.48141900	-0.24474300	-2.95447800
C	1.26174100	-1.05746200	0.17948200
C	1.48233300	1.06103000	0.76737400
N	1.71014100	-0.39769600	-0.89851100
N	1.12413600	-0.20279300	1.20090500
S	0.88619700	-2.76412700	0.23877000
C	2.57502200	-3.44535700	0.10747500
H	2.44942000	-4.52408100	0.19311900
H	3.02449600	-3.21126500	-0.85558600
H	3.19342300	-3.08364900	0.92601300
P	-2.39390700	0.13757200	-0.18642500
F	-3.06275800	1.43194100	-0.90590200
F	-3.64093000	0.05585100	0.85359400
F	-1.59897300	1.11985100	0.84710100
F	-1.71097200	-1.16144800	0.53877800
F	-1.13236300	0.21191500	-1.22056500
F	-3.17504200	-0.85151200	-1.21374100

2. benzimidazolium-PF_6-in

----

opt=calcfc freq=noraman wb97xd/6-311g(d,p) scrf=(iefpcm,solvent=dmso

) geom=connectivity temperature=363

Zero-point correction= 0.238439 (Hartree/Particle)

Thermal correction to Energy= 0.267701

Thermal correction to Enthalpy= 0.268851

Thermal correction to Gibbs Free Energy= 0.171075

Sum of electronic and zero-point Energies= -1836.957009

Sum of electronic and thermal Energies= -1836.927747

Sum of electronic and thermal Enthalpies= -1836.926598

Sum of electronic and thermal Free Energies= -1837.024373

Title Card Required

0 1

C(PDBName=C1,ResName=UNK,ResNum=900)	-2.19222400	0.29897200	-0.51151300
S(PDBName=S3,ResName=UNK,ResNum=900)	-0.53138700	-3.04915200	0.64376200
C(PDBName=C4,ResName=UNK,ResNum=900)	-1.16233900	-1.43880600	0.39125100
C(PDBName=C5,ResName=UNK,ResNum=900)	0.79827900	-3.09917000	-0.60698600
N(PDBName=N2,ResName=UNK,ResNum=900)	-1.12336800	-0.45009200	1.29445500
N(PDBName=N3,ResName=UNK,ResNum=900)	-1.82197400	-1.02083800	-0.70002700
C(PDBName=C6,ResName=UNK,ResNum=900)	-2.08561100	-1.78878100	-1.90619500
C(PDBName=C7,ResName=UNK,ResNum=900)	-0.48370700	-0.46262900	2.60258800
C(PDBName=C8,ResName=UNK,ResNum=900)	-1.76280400	0.65547200	0.76298600
C(PDBName=C9,ResName=UNK,ResNum=900)	-1.98345900	1.92623400	1.28449700
C(PDBName=C10,ResName=UNK,ResNum=900)	-2.85890600	1.19803000	-1.33919900
C(PDBName=C11,ResName=UNK,ResNum=900)	-3.07917900	2.46152000	-0.82329100
C(PDBName=C12,ResName=UNK,ResNum=900)	-2.64943200	2.81969100	0.46723900
H(PDBName=H1,ResName=UNK,ResNum=900)	0.39343600	-3.04431900	-1.61488800
H(PDBName=H2,ResName=UNK,ResNum=900)	1.50774300	-2.29953900	-0.42067400
H(PDBName=H3,ResName=UNK,ResNum=900)	1.27165800	-4.06920600	-0.45974000
H(PDBName=H4,ResName=UNK,ResNum=900)	-2.05629400	-2.84945500	-1.66616200
H(PDBName=H5,ResName=UNK,ResNum=900)	-3.07968900	-1.53562600	-2.26983400
H(PDBName=H6,ResName=UNK,ResNum=900)	-1.34151200	-1.55165900	-2.66690700
H(PDBName=H7,ResName=UNK,ResNum=900)	-1.24332900	-0.35118800	3.37601800
H(PDBName=H8,ResName=UNK,ResNum=900)	0.04014100	-1.40518000	2.72912100
H(PDBName=H9,ResName=UNK,ResNum=900)	0.23003600	0.35798700	2.63883400
H(PDBName=H10,ResName=UNK,ResNum=900)	-3.59443900	3.19653100	-1.42924100
H(PDBName=H11,ResName=UNK,ResNum=900)	-1.64134900	2.20182100	2.27308700
H(PDBName=H12,ResName=UNK,ResNum=900)	-2.84130700	3.82318700	0.82640200
H(PDBName=H13,ResName=UNK,ResNum=900)	-3.18447000	0.92621300	-2.33476000
P	2.16969200	0.73390100	-0.22472100
F	3.10672000	-0.22615700	-1.14675700
F	2.02468800	-0.47391500	0.87001100

F	0.84613700	0.17259600	-1.00341000
F	1.22095900	1.67604800	0.70742300
F	2.30059100	1.93060800	-1.31538800
F	3.48644700	1.28211400	0.55480100

3. PF_6

----

opt=calcfc freq=noraman wb97xd/6-311g(d,p) scrf=(iefpcm,solvent=dmso
) geom=connectivity temperature=363

Zero-point correction= 0.018873 (Hartree/Particle)
Thermal correction to Energy= 0.027562
Thermal correction to Enthalpy= 0.028712
Thermal correction to Gibbs Free Energy= -0.019673
Sum of electronic and zero-point Energies= -940.748703
Sum of electronic and thermal Energies= -940.740014
Sum of electronic and thermal Enthalpies= -940.738864
Sum of electronic and thermal Free Energies= -940.787249

Title Card Required

-1 1

P	0.00000300	0.00000700	0.00000500
F	0.59464100	0.60073200	-1.39316300
F	1.50775200	-0.40491000	0.46847200
F	-0.17355300	-1.45978700	-0.70335500
F	-0.59462900	-0.60075300	1.39328300
F	-1.50775700	0.40498200	-0.46854800
F	0.17354100	1.45972500	0.70330400

4. TS-bimidazolium-PF_6-py

----

opt=(calcfc,ts,noeigen) freq=noraman wb97xd/6-311g(d,p) scrf=(iefpcm
,solvent=dmso) geom=connectivity temperature=363

Zero-point correction= 0.328302 (Hartree/Particle)

Thermal correction to Energy= 0.365650

Thermal correction to Enthalpy= 0.366800

Thermal correction to Gibbs Free Energy= 0.244929

Sum of electronic and zero-point Energies= -2085.088601

Sum of electronic and thermal Energies= -2085.051253

Sum of electronic and thermal Enthalpies= -2085.050103

Sum of electronic and thermal Free Energies= -2085.171974

Title Card Required

0 1

C	-0.38995500	1.80061900	-0.50764600
C	-0.97953500	1.73680900	0.75136100
N	-0.62054600	0.51791100	1.30023600
N	0.29389900	0.60910600	-0.68611100
C	-1.06240000	0.05341600	2.60276000
H	-2.13665700	-0.12233800	2.56920700
H	-0.54456200	-0.87386200	2.83310200
H	-0.82121300	0.80675000	3.35316400
C	0.93734300	0.21683400	-1.92686200
H	1.91679800	0.68789900	-2.02096400
H	1.04252100	-0.86570000	-1.93898600
H	0.30355000	0.52089500	-2.75938000
C	0.14367400	-0.15846000	0.41779200
S	0.83501100	-1.70748000	0.66372200
C	3.06969800	-1.15673300	0.33344200
H	2.86390700	-0.10861600	0.47126200
H	3.34103300	-1.76081300	1.18244800
H	3.06299200	-1.58962000	-0.65153900
C	5.65885100	-0.88235400	-1.09440900
C	6.98641900	-0.54148300	-1.29888000
C	7.69244800	0.03718700	-0.25220300

C	7.04878900	0.25413100	0.95942200
C	5.71949000	-0.11729600	1.07932600
N	5.04771900	-0.67293000	0.07095900
H	8.73175400	0.31446000	-0.37956500
H	5.06442300	-1.33508700	-1.88075400
H	7.45050600	-0.72620700	-2.25859100
H	7.56204800	0.70268000	1.79967600
H	5.17108400	0.03056000	2.00349500
C	-1.76406500	2.77097700	1.24358000
C	-0.54884200	2.90521500	-1.33465800
C	-1.32878000	3.94216900	-0.84503200
C	-1.92612400	3.87571700	0.42188500
H	-2.23586300	2.71134300	2.21555500
H	-2.53168000	4.70591100	0.76462700
H	-1.48114100	4.82362100	-1.45589400
H	-0.08719300	2.95699100	-2.31240800
P	-3.74922700	-1.08999700	-0.29449000
F	-4.72996900	-0.26405500	-1.29823000
F	-2.45011900	-0.44305600	-1.03863800
F	-3.71997200	-2.33837500	-1.33959600
F	-2.76441700	-1.90950600	0.71452400
F	-5.04670000	-1.73779400	0.44644300
F	-3.77589400	0.15852700	0.75644000

5. TS-b-py-PF_6-(2Step)

opt=(calcfc,ts,noeigen) freq=noraman wb97xd/6-311g(d,p) scrf=(iefpcm
,solvent=dmso) geom=connectivity temperature=363

Zero-point correction= 0.236685 (Hartree/Particle)

Thermal correction to Energy= 0.265769

Thermal correction to Enthalpy= 0.266918

Thermal correction to Gibbs Free Energy= 0.168338

Sum of electronic and zero-point Energies= -1836.878593

Sum of electronic and thermal Energies= -1836.849510

Sum of electronic and thermal Enthalpies= -1836.848360

Sum of electronic and thermal Free Energies= -1836.946941

Title Card Required

0 1

C(PDBName=C,ResName=UNK,ResNum=0)	-3.18259100	-0.30944800	-0.38993400
C(PDBName=C,ResName=UNK,ResNum=0)	-2.34945500	-0.82879200	0.59829700
N(PDBName=N,ResName=UNK,ResNum=0)	-1.57407100	0.22419500	1.05921500
N(PDBName=N,ResName=UNK,ResNum=0)	-2.87509400	1.03734700	-0.50420500
C(PDBName=C,ResName=UNK,ResNum=0)	-0.59421100	0.10735200	2.12502900
C(PDBName=C,ResName=UNK,ResNum=0)	-3.50863400	1.93657100	-1.45083400
C(PDBName=C,ResName=UNK,ResNum=0)	-1.89387100	1.35504800	0.37733700
S(PDBName=S,ResName=UNK,ResNum=0)	-1.16030400	2.88137300	0.56719000
C(PDBName=C,ResName=UNK,ResNum=0)	1.20481400	1.88217900	-0.22304200
C(PDBName=C,ResName=UNK,ResNum=0)	-2.38945300	-2.16990800	0.95536400
C(PDBName=C,ResName=UNK,ResNum=0)	-4.10101300	-1.10388900	-1.06306400
C(PDBName=C,ResName=UNK,ResNum=0)	-4.14600700	-2.44478900	-0.70690700
C(PDBName=C,ResName=UNK,ResNum=0)	-3.30446700	-2.96820400	0.28313100
H(PDBName=H,ResName=UNK,ResNum=0)	-0.37992300	1.10116300	2.51193100
H(PDBName=H,ResName=UNK,ResNum=0)	-1.01307600	-0.50365800	2.92422300
H(PDBName=H,ResName=UNK,ResNum=0)	0.32081600	-0.35372100	1.75161200
H(PDBName=H,ResName=UNK,ResNum=0)	-4.56794400	2.03991300	-1.21217600
H(PDBName=H,ResName=UNK,ResNum=0)	-3.02270600	2.90642700	-1.38376700
H(PDBName=H,ResName=UNK,ResNum=0)	-3.39548700	1.53829300	-2.45986100
H(PDBName=H,ResName=UNK,ResNum=0)	1.39072700	1.83801300	0.83680500
H(PDBName=H,ResName=UNK,ResNum=0)	1.35833300	2.81902800	-0.73552800
H(PDBName=H,ResName=UNK,ResNum=0)	0.55771600	1.14869400	-0.67466000
H(PDBName=H,ResName=UNK,ResNum=0)	-1.73479000	-2.57601900	1.71569500
H(PDBName=H,ResName=UNK,ResNum=0)	-3.36847700	-4.02120700	0.52870800
H(PDBName=H,ResName=UNK,ResNum=0)	-4.84638000	-3.10158000	-1.20820900
H(PDBName=H,ResName=UNK,ResNum=0)	-4.75068500	-0.69899500	-1.82845600
P	3.19885300	-0.50908100	-0.22283200
F	4.66372300	0.11831400	-0.05973600

F	3.65118800	-1.96942800	0.21861000
F	2.84133400	-0.05706800	1.28506500
F	1.65641400	-0.94368200	-0.43074300
F	2.68594300	1.15967600	-0.72457400
F	3.47555900	-0.77110500	-1.77931100

6. TS-b-py-PF_6-competing(2Step)

opt=(calcfc,ts,noeigen) freq=noraman wb97xd/6-311g(d,p) scrf=(iefpcm

,solvent=dmso) geom=connectivity temperature=363

Zero-point correction= 0.327883 (Hartree/Particle)

Thermal correction to Energy= 0.365089

Thermal correction to Enthalpy= 0.366239

Thermal correction to Gibbs Free Energy= 0.249063

Sum of electronic and zero-point Energies= -2085.066685

Sum of electronic and thermal Energies= -2085.029479

Sum of electronic and thermal Enthalpies= -2085.028330

Sum of electronic and thermal Free Energies= -2085.145505

Title Card Required

0 1

C(PDBName=C,ResName=UNK,ResNum=0)	3.21852600	0.38378000	-0.52967800
C(PDBName=C,ResName=UNK,ResNum=0)	2.24205300	0.07358500	-1.47337800
N(PDBName=N,ResName=UNK,ResNum=0)	1.22274600	0.99751800	-1.31024900
N(PDBName=N,ResName=UNK,ResNum=0)	2.75053600	1.47608800	0.18302900
C(PDBName=C,ResName=UNK,ResNum=0)	0.00738000	1.01684000	-2.10176000
C(PDBName=C,ResName=UNK,ResNum=0)	3.47456600	2.10663900	1.26891800
C(PDBName=C,ResName=UNK,ResNum=0)	1.52841200	1.83559900	-0.28713100
S(PDBName=S,ResName=UNK,ResNum=0)	0.55610900	3.09821700	0.30882400
C(PDBName=C,ResName=UNK,ResNum=0)	-1.69887200	1.63353800	0.76517400
C(PDBName=C,ResName=UNK,ResNum=0)	1.25570600	-0.38585700	2.30412400
C(PDBName=C,ResName=UNK,ResNum=0)	2.34846100	-1.16322200	2.66102700
C(PDBName=C,ResName=UNK,ResNum=0)	2.58260000	-2.33770300	1.95725000

C(PDBName=C,ResName=UNK,ResNum=0)	1.71291200	-2.68659600	0.93406800
C(PDBName=C,ResName=UNK,ResNum=0)	0.64514100	-1.84325900	0.65518100
N(PDBName=N,ResName=UNK,ResNum=0)	0.41397300	-0.71054800	1.32094300
C(PDBName=C,ResName=UNK,ResNum=0)	2.38243200	-0.99990400	-2.34169000
C(PDBName=C,ResName=UNK,ResNum=0)	4.38610200	-0.35766800	-0.41746800
C(PDBName=C,ResName=UNK,ResNum=0)	4.53069500	-1.43182400	-1.28458100
C(PDBName=C,ResName=UNK,ResNum=0)	3.54632300	-1.74754600	-2.22890300
H(PDBName=H,ResName=UNK,ResNum=0)	-0.42331000	2.01492300	-2.05863200
H(PDBName=H,ResName=UNK,ResNum=0)	0.25649800	0.78423300	-3.13656300
H(PDBName=H,ResName=UNK,ResNum=0)	-0.70923100	0.28650400	-1.72277700
H(PDBName=H,ResName=UNK,ResNum=0)	2.79895500	2.77609600	1.79572000
H(PDBName=H,ResName=UNK,ResNum=0)	3.83150100	1.33705000	1.95426600
H(PDBName=H,ResName=UNK,ResNum=0)	4.32167600	2.67280700	0.87851000
H(PDBName=H,ResName=UNK,ResNum=0)	-1.98076300	2.04218300	-0.19109500
H(PDBName=H,ResName=UNK,ResNum=0)	-1.81698700	2.26946200	1.63025500
H(PDBName=H,ResName=UNK,ResNum=0)	-0.99236800	0.80788200	0.82494900
H(PDBName=H,ResName=UNK,ResNum=0)	3.42979400	-2.96781500	2.20143200
H(PDBName=H,ResName=UNK,ResNum=0)	1.04886000	0.54722400	2.82124500
H(PDBName=H,ResName=UNK,ResNum=0)	2.99982900	-0.85085800	3.46789600
H(PDBName=H,ResName=UNK,ResNum=0)	1.85803700	-3.58992300	0.35543500
H(PDBName=H,ResName=UNK,ResNum=0)	-0.05757400	-2.07988000	-0.13729200
H(PDBName=H,ResName=UNK,ResNum=0)	1.61776900	-1.25184100	-3.06524600
H(PDBName=H,ResName=UNK,ResNum=0)	3.69471700	-2.59596700	-2.88587000
H(PDBName=H,ResName=UNK,ResNum=0)	5.42532800	-2.03996700	-1.22793100
H(PDBName=H,ResName=UNK,ResNum=0)	5.14470800	-0.11498800	0.31551800
P	-3.84737900	-0.43664900	-0.15791800
F	-5.25676000	0.21809400	0.23613600
F	-4.49107300	-1.45297200	-1.20311000
F	-3.62572100	0.71429200	-1.26564800
F	-2.34533100	-0.96031500	-0.43507000
F	-3.12595100	0.71601400	1.03498500
F	-3.96848800	-1.45607800	1.07324000

7. TS-bimidazolium-TFSI-in

----

opt=(calcfc,ts,noeigen) freq=noraman wb97xd/6-311g(d,p) scrf=(iefpcm
,solvent=dmso) geom=connectivity temperature=363

Zero-point correction= 0.364819 (Hartree/Particle)
Thermal correction to Energy= 0.412306
Thermal correction to Enthalpy= 0.413456
Thermal correction to Gibbs Free Energy= 0.272278
Sum of electronic and zero-point Energies= -2971.614252
Sum of electronic and thermal Energies= -2971.566765
Sum of electronic and thermal Enthalpies= -2971.565615
Sum of electronic and thermal Free Energies= -2971.706793

Title Card Required

0 1

C(PDBName=C,ResName=UNK,ResNum=0)	3.05388700	-0.20802500	-0.60880700
C(PDBName=C,ResName=UNK,ResNum=0)	3.56645400	-1.02982500	0.39184400
N(PDBName=N,ResName=UNK,ResNum=0)	3.03720100	-2.29105400	0.18281900
N(PDBName=N,ResName=UNK,ResNum=0)	2.23586900	-1.00264900	-1.39462300
C(PDBName=C,ResName=UNK,ResNum=0)	3.25699700	-3.41776100	1.07073300
C(PDBName=C,ResName=UNK,ResNum=0)	1.44543900	-0.51632600	-2.51146700
C(PDBName=C,ResName=UNK,ResNum=0)	2.21923800	-2.25528000	-0.89110100
S(PDBName=S,ResName=UNK,ResNum=0)	1.33471300	-3.58490600	-1.51981500
S(PDBName=S,ResName=UNK,ResNum=0)	-0.13899400	0.39965100	1.35121700
O(PDBName=O,ResName=UNK,ResNum=0)	0.54691300	-0.86159000	1.13906700
C(PDBName=C,ResName=UNK,ResNum=0)	-0.82094000	-2.79196600	-1.29429100
C(PDBName=C,ResName=UNK,ResNum=0)	-3.02497000	-0.84633300	-1.32334700
C(PDBName=C,ResName=UNK,ResNum=0)	-4.23409900	-0.25072600	-1.00182800
C(PDBName=C,ResName=UNK,ResNum=0)	-5.20841100	-1.02028600	-0.38095500
C(PDBName=C,ResName=UNK,ResNum=0)	-4.94059200	-2.35571200	-0.10513500
C(PDBName=C,ResName=UNK,ResNum=0)	-3.70089000	-2.86805500	-0.45144300
N(PDBName=N,ResName=UNK,ResNum=0)	-2.76958800	-2.12365700	-1.04657300
C(PDBName=C,ResName=UNK,ResNum=0)	4.42379800	-0.54419200	1.37003000

C(PDBName=C,ResName=UNK,ResNum=0)	3.38216000	1.13841600	-0.68366400
C(PDBName=C,ResName=UNK,ResNum=0)	4.23566800	1.62924800	0.29282500
C(PDBName=C,ResName=UNK,ResNum=0)	4.74645100	0.80306800	1.30306500
O(PDBName=O,ResName=UNK,ResNum=0)	0.48622100	1.40596000	2.18725900
C(PDBName=C,ResName=UNK,ResNum=0)	-1.68776600	-0.11097300	2.27338900
H(PDBName=H,ResName=UNK,ResNum=0)	2.82440000	-3.19868600	2.04806900
H(PDBName=H,ResName=UNK,ResNum=0)	2.77711000	-4.29559400	0.64702100
H(PDBName=H,ResName=UNK,ResNum=0)	4.32716500	-3.59844400	1.17158700
H(PDBName=H,ResName=UNK,ResNum=0)	1.17863200	-1.35869800	-3.14590500
H(PDBName=H,ResName=UNK,ResNum=0)	0.54878200	-0.02316900	-2.13270600
H(PDBName=H,ResName=UNK,ResNum=0)	2.04376000	0.18878200	-3.08674400
H(PDBName=H,ResName=UNK,ResNum=0)	-0.51398300	-2.03315600	-0.59077600
H(PDBName=H,ResName=UNK,ResNum=0)	-0.90624400	-2.56723900	-2.34291600
H(PDBName=H,ResName=UNK,ResNum=0)	-1.12759600	-3.75848200	-0.93041200
H(PDBName=H,ResName=UNK,ResNum=0)	-6.16347800	-0.58531900	-0.11206500
H(PDBName=H,ResName=UNK,ResNum=0)	-2.23018700	-0.27987600	-1.79143300
H(PDBName=H,ResName=UNK,ResNum=0)	-4.38600600	0.79656700	-1.22611800
H(PDBName=H,ResName=UNK,ResNum=0)	-5.67204700	-2.99047400	0.37763200
H(PDBName=H,ResName=UNK,ResNum=0)	-3.43850100	-3.89966800	-0.24213400
H(PDBName=H,ResName=UNK,ResNum=0)	4.81525300	-1.18338000	2.15107600
H(PDBName=H,ResName=UNK,ResNum=0)	5.40845400	1.22575300	2.04897200
H(PDBName=H,ResName=UNK,ResNum=0)	4.50960100	2.67697000	0.27503400
H(PDBName=H,ResName=UNK,ResNum=0)	2.98548300	1.77902000	-1.45866000
F	-2.15766800	-1.24591700	1.77232700
F	-2.63034000	0.81565700	2.19139300
F	-1.37835300	-0.30300400	3.55010800
N	-0.66224100	0.90387800	-0.07120300
S	-1.23101500	2.36515100	-0.39103700
O	-1.66102200	3.14643000	0.75271500
O	-2.10885200	2.25753000	-1.54004600
C	0.25062300	3.28572900	-1.04763900
F	-0.12991100	4.50687200	-1.40282100
F	1.19268600	3.37947500	-0.12137100

F	0.74926600	2.66785900	-2.11447100

8. TS-benzimidazole-TFSI

```
 ----------------------------------------------------------------------
 # opt=(calcfc,ts,noeigen) freq=noraman wb97xd/6-311g(d,p) scrf=(iefpcm
 ,solvent=dmso) geom=connectivity temperature=363
 ----------------------------------------------------------------------
```

Zero-point correction= 0.362626 (Hartree/Particle)
Thermal correction to Energy= 0.410825
Thermal correction to Enthalpy= 0.411975
Thermal correction to Gibbs Free Energy= 0.267035
Sum of electronic and zero-point Energies= -2971.562854
Sum of electronic and thermal Energies= -2971.514655
Sum of electronic and thermal Enthalpies= -2971.513506
Sum of electronic and thermal Free Energies= -2971.658445

Title Card Required

0 1

C(PDBName=C1,ResName=UNK,ResNum=900)	-1.24800100	0.23475000	1.49023900
C(PDBName=C2,ResName=UNK,ResNum=900)	-1.39568500	1.26779100	0.56936800
N(PDBName=N1,ResName=UNK,ResNum=900)	-1.46683600	0.68140100	-0.68110700
N(PDBName=N2,ResName=UNK,ResNum=900)	-1.22584400	-0.94314200	0.76775300
C(PDBName=C3,ResName=UNK,ResNum=900)	-1.59195100	1.42267000	-1.92326200
C(PDBName=C4,ResName=UNK,ResNum=900)	-1.12009900	-2.26000600	1.36529500
C(PDBName=C5,ResName=UNK,ResNum=900)	-1.36435500	-0.66202800	-0.54852800
S(PDBName=S1,ResName=UNK,ResNum=900)	-1.48468300	-1.80237100	-1.81084400
S(PDBName=S2,ResName=UNK,ResNum=900)	2.44861500	0.79618000	-1.31097000
S(PDBName=S3,ResName=UNK,ResNum=900)	2.61278700	-0.78320000	1.03278000
C(PDBName=C6,ResName=UNK,ResNum=900)	2.86114300	-2.48331200	0.30829400
C(PDBName=C7,ResName=UNK,ResNum=900)	2.43377300	2.54500800	-0.66323000
O(PDBName=O1,ResName=UNK,ResNum=900)	1.51214900	0.82211600	-2.41758500
O(PDBName=O2,ResName=UNK,ResNum=900)	3.84460800	0.51987100	-1.59336800
O(PDBName=O3,ResName=UNK,ResNum=900)	1.72768700	-1.00371800	2.15948300

O(PDBName=O4,ResName=UNK,ResNum=900)	3.95158500	-0.28990900	1.29430400
F(PDBName=F1,ResName=UNK,ResNum=900)	1.69049700	-3.06940300	0.08884100
F(PDBName=F2,ResName=UNK,ResNum=900)	3.52508800	-2.41178400	-0.83749100
F(PDBName=F3,ResName=UNK,ResNum=900)	3.55687000	-3.22321300	1.16495900
F(PDBName=F4,ResName=UNK,ResNum=900)	3.01662800	2.60973400	0.52633600
F(PDBName=F5,ResName=UNK,ResNum=900)	3.09347900	3.32733300	-1.51010200
F(PDBName=F6,ResName=UNK,ResNum=900)	1.18677700	2.98866900	-0.55629700
N(PDBName=N3,ResName=UNK,ResNum=900)	1.79252600	-0.03067500	-0.11280500
C(PDBName=C8,ResName=UNK,ResNum=900)	-3.71822700	-3.18381200	-1.40876400
C(PDBName=C9,ResName=UNK,ResNum=900)	-4.58049900	-0.16374300	-1.51408800
C(PDBName=C10,ResName=UNK,ResNum=900)	-4.92564500	1.10932700	-1.08689000
C(PDBName=C11,ResName=UNK,ResNum=900)	-4.97472300	1.36344600	0.27794200
C(PDBName=C12,ResName=UNK,ResNum=900)	-4.70779400	0.33087200	1.16680000
C(PDBName=C13,ResName=UNK,ResNum=900)	-4.39941200	-0.91947700	0.65292500
N(PDBName=N4,ResName=UNK,ResNum=900)	-4.32433100	-1.14724700	-0.65574500
C(PDBName=C14,ResName=UNK,ResNum=900)	-1.48864100	2.59493000	0.96944500
C(PDBName=C15,ResName=UNK,ResNum=900)	-1.19416800	0.47627900	2.85682800
C(PDBName=C16,ResName=UNK,ResNum=900)	-1.28910100	1.79863400	3.26034500
C(PDBName=C17,ResName=UNK,ResNum=900)	-1.43362300	2.84053500	2.33183700
H(PDBName=H1,ResName=UNK,ResNum=900)	-0.63251600	1.86698300	-2.18253800
H(PDBName=H2,ResName=UNK,ResNum=900)	-1.89593600	0.73500600	-2.70873900
H(PDBName=H3,ResName=UNK,ResNum=900)	-2.35201900	2.19367500	-1.79933700
H(PDBName=H4,ResName=UNK,ResNum=900)	-2.03820000	-2.50050700	1.90556400
H(PDBName=H5,ResName=UNK,ResNum=900)	-0.95853400	-2.98900400	0.57616500
H(PDBName=H6,ResName=UNK,ResNum=900)	-0.27155700	-2.26359200	2.04692800
H(PDBName=H7,ResName=UNK,ResNum=900)	-2.84998400	-3.77718000	-1.63706800
H(PDBName=H8,ResName=UNK,ResNum=900)	-4.18591800	-3.40295400	-0.46128100
H(PDBName=H9,ResName=UNK,ResNum=900)	-4.36322000	-2.92558000	-2.23531400
H(PDBName=H10,ResName=UNK,ResNum=900)	-5.21596400	2.35366800	0.64439600
H(PDBName=H11,ResName=UNK,ResNum=900)	-4.48717400	-0.40170700	-2.56855300
H(PDBName=H12,ResName=UNK,ResNum=900)	-5.13419200	1.88377800	-1.81349700
H(PDBName=H13,ResName=UNK,ResNum=900)	-4.73100900	0.48433900	2.23765000
H(PDBName=H14,ResName=UNK,ResNum=900)	-4.20071500	-1.76230300	1.30765900
H(PDBName=H15,ResName=UNK,ResNum=900)	-1.59622400	3.39804900	0.25226800

H(PDBName=H16,ResName=UNK,ResNum=900)	-1.50441500	3.86063400	2.68936200
H(PDBName=H17,ResName=UNK,ResNum=900)	-1.24902600	2.03310800	4.31715700
H(PDBName=H18,ResName=UNK,ResNum=900)	-1.07974100	-0.32860300	3.57084100

9. benzimidazole-TFSI

\# opt=calcfc freq=noraman wb97xd/6-311g(d,p) scrf=(iefpcm,solvent=dmso

) geom=connectivity temperature=363

Zero-point correction= 0.274723 (Hartree/Particle)

Thermal correction to Energy= 0.314395

Thermal correction to Enthalpy= 0.315545

Thermal correction to Gibbs Free Energy= 0.193378

Sum of electronic and zero-point Energies= -2723.473586

Sum of electronic and thermal Energies= -2723.433913

Sum of electronic and thermal Enthalpies= -2723.432764

Sum of electronic and thermal Free Energies= -2723.554931

Title Card Required

0 1

C(PDBName=C1,ResName=UNK,ResNum=900)	-2.71787900	-0.92340400	-0.03505600
S(PDBName=S1,ResName=UNK,ResNum=900)	1.15837700	0.84484700	-1.46891200
S(PDBName=S2,ResName=UNK,ResNum=900)	0.99917700	-1.68339000	-0.19183700
C(PDBName=C2,ResName=UNK,ResNum=900)	2.66265200	-1.73156800	0.65517600
C(PDBName=C3,ResName=UNK,ResNum=900)	1.89279300	2.18234400	-0.40188200
O(PDBName=O1,ResName=UNK,ResNum=900)	2.25633200	0.35460300	-2.27975200
O(PDBName=O2,ResName=UNK,ResNum=900)	0.04601500	1.48994200	-2.14043300
O(PDBName=O3,ResName=UNK,ResNum=900)	1.18934400	-2.37573600	-1.45143600
O(PDBName=O4,ResName=UNK,ResNum=900)	0.11728600	-2.26627300	0.79971300
F(PDBName=F1,ResName=UNK,ResNum=900)	2.60380200	-1.06735000	1.80420300
F(PDBName=F2,ResName=UNK,ResNum=900)	2.97864400	-2.99674100	0.90617200
F(PDBName=F3,ResName=UNK,ResNum=900)	3.60594400	-1.19449800	-0.10451700
F(PDBName=F4,ResName=UNK,ResNum=900)	0.96430500	2.71113200	0.38919800

F(PDBName=F5,ResName=UNK,ResNum=900)	2.86626500	1.68804700	0.35053100
F(PDBName=F6,ResName=UNK,ResNum=900)	2.38643500	3.13546800	-1.18063800
N(PDBName=N1,ResName=UNK,ResNum=900)	0.63739400	-0.13671700	-0.31578800
S(PDBName=S3,ResName=UNK,ResNum=900)	-1.63437600	1.64137400	2.70464600
C(PDBName=C4,ResName=UNK,ResNum=900)	-2.14538200	0.74362600	1.29635800
C(PDBName=C5,ResName=UNK,ResNum=900)	-0.01910900	0.82905000	2.98245800
N(PDBName=N2,ResName=UNK,ResNum=900)	-2.33944700	1.27062700	0.07940200
N(PDBName=N3,ResName=UNK,ResNum=900)	-2.39911800	-0.57174400	1.26460700
C(PDBName=C6,ResName=UNK,ResNum=900)	-2.38296100	-1.48556600	2.39778100
C(PDBName=C7,ResName=UNK,ResNum=900)	-2.23133000	2.67186700	-0.30294400
C(PDBName=C8,ResName=UNK,ResNum=900)	-2.69781600	0.24979800	-0.78373500
C(PDBName=C9,ResName=UNK,ResNum=900)	-2.98697100	0.25701200	-2.14508100
C(PDBName=C10,ResName=UNK,ResNum=900)	-3.01308300	-2.15586700	-0.61087600
C(PDBName=C11,ResName=UNK,ResNum=900)	-3.29429300	-2.15204300	-1.96377400
C(PDBName=C12,ResName=UNK,ResNum=900)	-3.28267000	-0.96464200	-2.71859300
H(PDBName=H1,ResName=UNK,ResNum=900)	-0.14395700	-0.12520800	3.48912300
H(PDBName=H2,ResName=UNK,ResNum=900)	0.48372400	0.69990100	2.02749600
H(PDBName=H3,ResName=UNK,ResNum=900)	0.54432700	1.51347300	3.61458900
H(PDBName=H4,ResName=UNK,ResNum=900)	-2.54838400	-0.91805400	3.31129300
H(PDBName=H5,ResName=UNK,ResNum=900)	-3.19515500	-2.19959300	2.27314700
H(PDBName=H6,ResName=UNK,ResNum=900)	-1.42860600	-2.00701700	2.42964500
H(PDBName=H7,ResName=UNK,ResNum=900)	-3.22872600	3.08910700	-0.44087700
H(PDBName=H8,ResName=UNK,ResNum=900)	-1.71233700	3.21067200	0.48493100
H(PDBName=H9,ResName=UNK,ResNum=900)	-1.65644900	2.72916100	-1.22418600
H(PDBName=H10,ResName=UNK,ResNum=900)	-3.52365400	-3.08793500	-2.45817700
H(PDBName=H11,ResName=UNK,ResNum=900)	-2.96374900	1.16998100	-2.72446800
H(PDBName=H12,ResName=UNK,ResNum=900)	-3.50410600	-1.01297300	-3.77760700
H(PDBName=H13,ResName=UNK,ResNum=900)	-3.00963700	-3.07043400	-0.03308400

10. benzimidazolium-TFSI

----

opt=calcfc freq=noraman wb97xd/6-311g(d,p) scrf=(iefpcm,solvent=dmso

) geom=connectivity temperature=363

Zero-point correction= 0.365842 (Hartree/Particle)

Thermal correction to Energy= 0.414091

Thermal correction to Enthalpy= 0.415241

Thermal correction to Gibbs Free Energy= 0.270012

Sum of electronic and zero-point Energies= -2971.649279

Sum of electronic and thermal Energies= -2971.601029

Sum of electronic and thermal Enthalpies= -2971.599880

Sum of electronic and thermal Free Energies= -2971.745109

Title Card Required

0 1

C(PDBName=C1,ResName=UNK,ResNum=900)	1.69936800	-1.76578000	-1.56996200
C(PDBName=C2,ResName=UNK,ResNum=900)	2.23402200	-2.27519500	-0.39014300
N(PDBName=N1,ResName=UNK,ResNum=900)	1.16477500	-2.55125100	0.44554700
N(PDBName=N2,ResName=UNK,ResNum=900)	0.32603800	-1.73586200	-1.40444200
C(PDBName=C3,ResName=UNK,ResNum=900)	1.28464600	-3.08810300	1.79268700
C(PDBName=C4,ResName=UNK,ResNum=900)	-0.61167100	-1.16552400	-2.35816800
C(PDBName=C5,ResName=UNK,ResNum=900)	0.03247100	-2.21354900	-0.18831000
S(PDBName=S1,ResName=UNK,ResNum=900)	-1.56882400	-2.35635100	0.49234800
S(PDBName=S2,ResName=UNK,ResNum=900)	0.41643500	1.07733700	1.62858400
S(PDBName=S3,ResName=UNK,ResNum=900)	0.82708300	2.17919700	-0.95846200
C(PDBName=C6,ResName=UNK,ResNum=900)	-0.86081100	2.84780700	-1.38787400
C(PDBName=C7,ResName=UNK,ResNum=900)	2.17765200	0.99533300	2.24663100
O(PDBName=O1,ResName=UNK,ResNum=900)	-0.15443900	-0.16877000	2.10625300
O(PDBName=O2,ResName=UNK,ResNum=900)	-0.10291700	2.32898400	2.14567600
O(PDBName=O3,ResName=UNK,ResNum=900)	1.29461100	1.61832300	-2.21148600
O(PDBName=O4,ResName=UNK,ResNum=900)	1.53391300	3.30926300	-0.38734100
F(PDBName=F1,ResName=UNK,ResNum=900)	-1.46962700	3.33419900	-0.31509800
F(PDBName=F2,ResName=UNK,ResNum=900)	-0.71912600	3.82043400	-2.27997000
F(PDBName=F3,ResName=UNK,ResNum=900)	-1.61707500	1.89101700	-1.91563700
F(PDBName=F4,ResName=UNK,ResNum=900)	2.81121100	-0.03255200	1.69199300
F(PDBName=F5,ResName=UNK,ResNum=900)	2.84078200	2.10694300	1.97509400
F(PDBName=F6,ResName=UNK,ResNum=900)	2.15058500	0.81910100	3.56355000

N(PDBName=N3,ResName=UNK,ResNum=900)	0.53137600	0.96743900	0.03921300
C(PDBName=C8,ResName=UNK,ResNum=900)	-2.28045900	-3.59604200	-0.64083900
C(PDBName=C9,ResName=UNK,ResNum=900)	-4.71093500	-1.07987000	-0.75076900
C(PDBName=C10,ResName=UNK,ResNum=900)	-4.14700900	0.18684500	-0.82425500
C(PDBName=C11,ResName=UNK,ResNum=900)	-3.81920300	0.83616800	0.35901300
C(PDBName=C12,ResName=UNK,ResNum=900)	-4.07774500	0.19468900	1.56158200
C(PDBName=C13,ResName=UNK,ResNum=900)	-4.64518000	-1.07348400	1.52778800
N(PDBName=N4,ResName=UNK,ResNum=900)	-4.95879400	-1.71356700	0.39879500
C(PDBName=C14,ResName=UNK,ResNum=900)	3.60560900	-2.43456600	-0.21443500
C(PDBName=C15,ResName=UNK,ResNum=900)	2.50772500	-1.39031800	-2.63855300
C(PDBName=C16,ResName=UNK,ResNum=900)	3.86989900	-1.54767500	-2.46691100
C(PDBName=C17,ResName=UNK,ResNum=900)	4.41010900	-2.06258600	-1.27494200
H(PDBName=H1,ResName=UNK,ResNum=900)	1.56449500	-2.29061500	2.47932400
H(PDBName=H2,ResName=UNK,ResNum=900)	0.32731000	-3.50732500	2.09067500
H(PDBName=H3,ResName=UNK,ResNum=900)	2.04221500	-3.86996400	1.78970300
H(PDBName=H4,ResName=UNK,ResNum=900)	-0.85248000	-1.89423600	-3.13256500
H(PDBName=H5,ResName=UNK,ResNum=900)	-1.51149300	-0.86489300	-1.82458400
H(PDBName=H6,ResName=UNK,ResNum=900)	-0.14965600	-0.28394300	-2.79767200
H(PDBName=H7,ResName=UNK,ResNum=900)	-3.28233000	-3.76997000	-0.24882200
H(PDBName=H8,ResName=UNK,ResNum=900)	-2.36219200	-3.20803600	-1.65464100
H(PDBName=H9,ResName=UNK,ResNum=900)	-1.70180600	-4.51676800	-0.61675400
H(PDBName=H10,ResName=UNK,ResNum=900)	-3.36902300	1.82074900	0.34369700
H(PDBName=H11,ResName=UNK,ResNum=900)	-4.97129200	-1.61528700	-1.65947200
H(PDBName=H12,ResName=UNK,ResNum=900)	-3.96756500	0.64850900	-1.78688300
H(PDBName=H13,ResName=UNK,ResNum=900)	-3.83995400	0.65994700	2.50998800
H(PDBName=H14,ResName=UNK,ResNum=900)	-4.85362600	-1.60302600	2.45290000
H(PDBName=H15,ResName=UNK,ResNum=900)	4.02164200	-2.82211200	0.70607100
H(PDBName=H16,ResName=UNK,ResNum=900)	5.48412700	-2.16807000	-1.18475900
H(PDBName=H17,ResName=UNK,ResNum=900)	4.54050400	-1.26434300	-3.26871700
H(PDBName=H18,ResName=UNK,ResNum=900)	2.09120900	-0.98442500	-3.55026000

11. benimidazolium-TFSI

----

opt=calcfc freq=noraman wb97xd/6-311g(d,p) scrf=(iefpcm,solvent=dmso

) geom=connectivity temperature=363

Zero-point correction= 0.274339 (Hartree/Particle)

Thermal correction to Energy= 0.314234

Thermal correction to Enthalpy= 0.315384

Thermal correction to Gibbs Free Energy= 0.191276

Sum of electronic and zero-point Energies= -2723.470655

Sum of electronic and thermal Energies= -2723.430760

Sum of electronic and thermal Enthalpies= -2723.429610

Sum of electronic and thermal Free Energies= -2723.553718

Title Card Required

0 1

C	1.17189200	1.96743100	-0.10854700
S	-2.51495700	0.20255800	0.50748900
S	-0.71513800	-1.84353600	-0.30012000
C	-0.03057400	-2.45703900	1.31958800
C	-3.22780900	0.81604700	-1.10024400
O	-2.32955400	1.39664100	1.30373400
O	-3.44944000	-0.80457100	0.97182500
O	0.43299100	-1.84205800	-1.18816200
O	-1.81754200	-2.73373900	-0.60752200
F	1.01431800	-1.72104400	1.68484600
F	-0.95464400	-2.38945600	2.26859700
F	0.36065900	-3.71743900	1.18251700
F	-3.30047700	-0.17539500	-1.97972800
F	-4.44655200	1.29631200	-0.88754800
F	-2.46937900	1.78255500	-1.60849300
N	-1.08312200	-0.33002500	0.03486500
S	3.26261900	-0.96941900	-1.62647100
C	2.47726500	0.27806300	-0.68331300
C	4.89657200	-0.17737000	-1.82589800
H	5.46710800	-0.85993800	-2.45475300

H	4.79301800	0.78195400	-2.32843800
H	5.40299200	-0.06349600	-0.86911700
N	2.75094100	0.56567300	0.59920600
N	1.52770700	1.10787300	-1.13257600
C	0.88482300	1.08561600	-2.43692700
H	0.85507900	2.10013100	-2.83206600
H	1.45908300	0.44656100	-3.10142800
H	-0.12286100	0.69060400	-2.32008200
C	3.71489000	-0.10515400	1.45841800
H	3.26105900	-0.25795600	2.43588600
H	3.96027200	-1.07130700	1.02398200
H	4.61373100	0.50345800	1.55792200
C	1.95718600	1.63103300	0.99029100
C	1.86613900	2.31879300	2.19681300
C	0.25263200	3.00952700	-0.06019400
C	0.16364900	3.69779200	1.13405200
H	-0.54369500	4.51292000	1.22143100
C	0.95414400	3.35698200	2.24523000
H	2.47784200	2.05782000	3.05045200
H	0.84486500	3.92087400	3.16332600
H	-0.36938000	3.25977300	-0.90829400

12. TS-benzimidazolium-TFSI-py

----

opt=(calcfc,ts,noeigen) freq=noraman wb97xd/6-311g(d,p) scrf=(iefpcm ,solvent=dmso) geom=connectivity temperature=363

Zero-point correction= 0.364663 (Hartree/Particle)

Thermal correction to Energy= 0.412393

Thermal correction to Enthalpy= 0.413542

Thermal correction to Gibbs Free Energy= 0.268775

Sum of electronic and zero-point Energies= -2971.605932

Sum of electronic and thermal Energies= -2971.558202

Sum of electronic and thermal Enthalpies= -2971.557053

Sum of electronic and thermal Free Energies= -2971.701820

Title Card Required

0 1

C	-0.40322300	1.45769900	1.36069400
C	0.02433400	2.34428300	0.37691700
N	-0.34652600	1.79750000	-0.84071300
N	-1.00923100	0.39882800	0.70772900
C	-0.11046100	2.42505600	-2.12952900
H	0.91473200	2.24124200	-2.44709600
H	-0.79818800	1.99655400	-2.85481500
H	-0.29804800	3.49426400	-2.03571200
C	-1.56583300	-0.76230600	1.37867600
H	-2.53835600	-0.52153200	1.81084800
H	-1.66710400	-1.56506200	0.65295800
H	-0.87000600	-1.07415200	2.15520000
C	-0.95958800	0.61370100	-0.62526400
S	-1.60866700	-0.41394800	-1.83555000
S	2.97414200	-0.23516200	-1.32709300
S	2.36137700	-1.44789700	1.15625900
C	1.72195400	-3.10870100	0.59732200
C	3.90383300	1.30773700	-0.84062200
O	2.15948200	0.18558100	-2.45026500
O	4.00707600	-1.22853500	-1.55455700
O	1.49738300	-1.09274400	2.26492500
O	3.77056000	-1.66616700	1.42223200
F	2.35270600	-3.51294700	-0.49617900
F	1.92500600	-3.99540600	1.56602800
F	0.42044300	-3.04587800	0.34198500
F	3.08564100	2.35354300	-0.82477800
F	4.45161000	1.17196000	0.35900300
F	4.86372200	1.53191200	-1.73100200
N	2.01396700	-0.49248100	-0.07661800

C	-3.83206200	-0.35038800	-1.15285700
H	-4.14522400	-0.35977200	-2.18354600
H	-3.73642600	-1.27470000	-0.61229200
H	-3.67124100	0.59367900	-0.65972300
C	-6.74813500	-0.86348400	-1.27545900
C	-8.07566900	-0.85989500	-0.87751200
C	-8.41303500	-0.21443300	0.30520200
C	-7.41426600	0.40229100	1.04780400
C	-6.11204600	0.35143500	0.57765300
N	-5.79535500	-0.26757300	-0.55932000
H	-9.44147200	-0.19271600	0.64457600
H	-6.43154500	-1.35432700	-2.18939400
H	-8.82298000	-1.35343500	-1.48468600
H	-7.63416200	0.91412200	1.97534800
H	-5.29554200	0.81616100	1.12035600
C	0.67496400	3.52994200	0.69738600
C	-0.20228300	1.71131700	2.71124100
C	0.44850300	2.89095900	3.03440700
C	0.87897800	3.78612600	2.04372200
H	1.01626000	4.21674400	-0.06589100
H	1.38499700	4.69772900	2.33746200
H	0.63081800	3.12611900	4.07596800
H	-0.53007500	1.01550200	3.47214800

13. TS-benzimidazolium-py-TFSI

----

opt=(calcfc,ts,noeigen) freq=noraman wb97xd/6-311g(d,p) scrf=(iefpcm
,solvent=dmso) geom=connectivity temperature=363

Zero-point correction= 0.184826 (Hartree/Particle)
Thermal correction to Energy= 0.215039
Thermal correction to Enthalpy= 0.216189
Thermal correction to Gibbs Free Energy= 0.112499

Sum of electronic and zero-point Energies= -2115.094027

Sum of electronic and thermal Energies= -2115.063815

Sum of electronic and thermal Enthalpies= -2115.062665

Sum of electronic and thermal Free Energies= -2115.166354

Title Card Required

0 1

S(PDBName=S2,ResName=UNK,ResNum=900)	1.46913300	-1.20553300	-0.79862200
S(PDBName=S3,ResName=UNK,ResNum=900)	1.39605100	1.20925300	0.79380400
C(PDBName=C6,ResName=UNK,ResNum=900)	1.59819200	2.52631200	-0.51475100
C(PDBName=C7,ResName=UNK,ResNum=900)	1.60666300	-2.50887200	0.53173900
O(PDBName=O1,ResName=UNK,ResNum=900)	2.81771700	-0.85987100	-1.17261900
O(PDBName=O2,ResName=UNK,ResNum=900)	0.55463300	-1.76486400	-1.76747600
O(PDBName=O3,ResName=UNK,ResNum=900)	2.72481400	0.88451800	1.24886500
O(PDBName=O4,ResName=UNK,ResNum=900)	0.40955100	1.73813900	1.70583800
F(PDBName=F1,ResName=UNK,ResNum=900)	2.38796600	2.09926300	-1.48323900
F(PDBName=F2,ResName=UNK,ResNum=900)	0.41038100	2.83314300	-1.01766200
F(PDBName=F3,ResName=UNK,ResNum=900)	2.12771900	3.60086400	0.04778100
F(PDBName=F4,ResName=UNK,ResNum=900)	0.39527100	-2.80560100	0.97941000
F(PDBName=F5,ResName=UNK,ResNum=900)	2.15930400	-3.59039600	0.00583600
F(PDBName=F6,ResName=UNK,ResNum=900)	2.35107500	-2.07434000	1.53247500
N(PDBName=N3,ResName=UNK,ResNum=900)	0.67370100	0.00093600	-0.03700900
C(PDBName=C8,ResName=UNK,ResNum=900)	-1.24611500	-0.02401800	-0.03300600
C(PDBName=C9,ResName=UNK,ResNum=900)	-3.98844900	0.79270500	0.79299700
C(PDBName=C10,ResName=UNK,ResNum=900)	-5.37165300	0.85138100	0.84940500
C(PDBName=C11,ResName=UNK,ResNum=900)	-6.10612200	0.01361200	0.01981200
C(PDBName=C12,ResName=UNK,ResNum=900)	-5.43224100	-0.85042100	-0.83348000
C(PDBName=C13,ResName=UNK,ResNum=900)	-4.04613600	-0.84381900	-0.82513200
N(PDBName=N4,ResName=UNK,ResNum=900)	-3.34512700	-0.03826500	-0.02760900
H(PDBName=H7,ResName=UNK,ResNum=900)	-1.28402400	-0.78190900	-0.79790800
H(PDBName=H8,ResName=UNK,ResNum=900)	-1.32442400	1.01033400	-0.31877200
H(PDBName=H9,ResName=UNK,ResNum=900)	-1.30158700	-0.29891700	1.00540500
H(PDBName=H10,ResName=UNK,ResNum=900)	-7.18902900	0.03427300	0.03815100

H(PDBName=H11,ResName=UNK,ResNum=900)	-3.36907000	1.42610500	1.41949500
H(PDBName=H12,ResName=UNK,ResNum=900)	-5.85672300	1.53958300	1.52902100
H(PDBName=H13,ResName=UNK,ResNum=900)	-5.96636100	-1.51889800	-1.49576500
H(PDBName=H14,ResName=UNK,ResNum=900)	-3.47573100	-1.49985100	-1.47399100

TS-benzimidazolium-py-TFSI(2Step)--

opt=(calcfc,ts,noeigen) freq=noraman wb97xd/6-311g(d,p) scrf=(iefpcm
,solvent=dmso) geom=connectivity temperature=298

Zero-point correction= 0.363655 (Hartree/Particle)
Thermal correction to Energy= 0.398157
Thermal correction to Enthalpy= 0.399100
Thermal correction to Gibbs Free Energy= 0.292017
Sum of electronic and zero-point Energies= -2971.597242
Sum of electronic and thermal Energies= -2971.562741
Sum of electronic and thermal Enthalpies= -2971.561797
Sum of electronic and thermal Free Energies= -2971.668881

Title Card Required

0 1

C(PDBName=C1,ResName=UNK,ResNum=900)	-3.63747900	0.42948400	-0.44103200
C(PDBName=C2,ResName=UNK,ResNum=900)	-4.06691100	-0.41413700	0.57987400
N(PDBName=N1,ResName=UNK,ResNum=900)	-3.34407500	-0.07096200	1.71155900
N(PDBName=N2,ResName=UNK,ResNum=900)	-2.67587800	1.26204900	0.10859500
C(PDBName=C3,ResName=UNK,ResNum=900)	-3.46540600	-0.74951700	2.98955900
C(PDBName=C4,ResName=UNK,ResNum=900)	-1.96932700	2.28959400	-0.63812800
C(PDBName=C5,ResName=UNK,ResNum=900)	-2.49987200	0.94430600	1.41454500
S(PDBName=S1,ResName=UNK,ResNum=900)	-1.33473900	1.63542700	2.46051700
S(PDBName=S2,ResName=UNK,ResNum=900)	0.90284300	-1.35956500	-1.23628300
S(PDBName=S3,ResName=UNK,ResNum=900)	2.91576100	-1.42439900	0.84014700
C(PDBName=C6,ResName=UNK,ResNum=900)	4.20560500	-0.24186200	0.18907000
C(PDBName=C7,ResName=UNK,ResNum=900)	-0.27984000	-2.68721900	-0.65842400
O(PDBName=O1,ResName=UNK,ResNum=900)	1.84252000	-2.00316700	-2.12085200

O(PDBName=O2,ResName=UNK,ResNum=900)	0.04262900	-0.30393500	-1.72528600
O(PDBName=O3,ResName=UNK,ResNum=900)	3.29349800	-2.73244600	0.36229600
O(PDBName=O4,ResName=UNK,ResNum=900)	2.83399900	-1.15556000	2.25625400
F(PDBName=F1,ResName=UNK,ResNum=900)	4.15789200	-0.19607100	-1.13372900
F(PDBName=F2,ResName=UNK,ResNum=900)	3.98499300	0.96761900	0.67756900
F(PDBName=F3,ResName=UNK,ResNum=900)	5.40140400	-0.66071800	0.57161200
F(PDBName=F4,ResName=UNK,ResNum=900)	-1.16510600	-2.15981400	0.17917800
F(PDBName=F5,ResName=UNK,ResNum=900)	-0.91345200	-3.17789400	-1.71052800
F(PDBName=F6,ResName=UNK,ResNum=900)	0.37792600	-3.65490300	-0.04462100
N(PDBName=N3,ResName=UNK,ResNum=900)	1.55624600	-0.83327400	0.15983500
C(PDBName=C8,ResName=UNK,ResNum=900)	0.37114700	0.32729600	1.28430800
C(PDBName=C9,ResName=UNK,ResNum=900)	1.61699400	2.67782000	-0.97772800
C(PDBName=C10,ResName=UNK,ResNum=900)	1.10333600	3.38663000	-2.05599800
C(PDBName=C11,ResName=UNK,ResNum=900)	0.51904000	4.62289700	-1.81668300
C(PDBName=C12,ResName=UNK,ResNum=900)	0.47495000	5.09585100	-0.51108600
C(PDBName=C13,ResName=UNK,ResNum=900)	1.01795400	4.31012000	0.49785900
N(PDBName=N4,ResName=UNK,ResNum=900)	1.58178400	3.11961300	0.28039700
C(PDBName=C14,ResName=UNK,ResNum=900)	-5.03424900	-1.38749600	0.36523900
C(PDBName=C15,ResName=UNK,ResNum=900)	-4.15201400	0.34164500	-1.72810800
C(PDBName=C16,ResName=UNK,ResNum=900)	-5.11712400	-0.63010900	-1.94679200
C(PDBName=C17,ResName=UNK,ResNum=900)	-5.55028300	-1.47938800	-0.91878500
H(PDBName=H1,ResName=UNK,ResNum=900)	-3.29458400	-1.81693300	2.84669800
H(PDBName=H2,ResName=UNK,ResNum=900)	-2.71689600	-0.34647200	3.66686300
H(PDBName=H3,ResName=UNK,ResNum=900)	-4.46130900	-0.58598000	3.40239000
H(PDBName=H4,ResName=UNK,ResNum=900)	-1.36165000	1.82287600	-1.41403600
H(PDBName=H5,ResName=UNK,ResNum=900)	-2.69071900	2.97323000	-1.08608600
H(PDBName=H6,ResName=UNK,ResNum=900)	-1.32728400	2.83764100	0.04745100
H(PDBName=H7,ResName=UNK,ResNum=900)	0.24128400	-0.44407500	2.02276300
H(PDBName=H8,ResName=UNK,ResNum=900)	1.10490700	1.11111500	1.40841700
H(PDBName=H9,ResName=UNK,ResNum=900)	-0.29365700	0.39526000	0.44289800
H(PDBName=H10,ResName=UNK,ResNum=900)	0.10307900	5.20532800	-2.63040600
H(PDBName=H11,ResName=UNK,ResNum=900)	2.06803500	1.70317700	-1.13020000
H(PDBName=H12,ResName=UNK,ResNum=900)	1.15847800	2.97067700	-3.05416600
H(PDBName=H13,ResName=UNK,ResNum=900)	0.02626200	6.05245600	-0.27429100

H(PDBName=H14,ResName=UNK,ResNum=900) 0.99463700 4.64871500 1.52930700

H(PDBName=H15,ResName=UNK,ResNum=900) -5.36698400 -2.04472400 1.15830300

H(PDBName=H16,ResName=UNK,ResNum=900) -6.30517400 -2.22591200 -1.13287200

H(PDBName=H17,ResName=UNK,ResNum=900) -5.54625300 -0.73656300 -2.93552500

H(PDBName=H18,ResName=UNK,ResNum=900) -3.81378800 0.99621300 -2.52107700

TS-benzimidazolium-py-TFSI(2Step)-v2---

opt=(calcfc,ts,noeigen) freq=noraman wb97xd/6-311g(d,p) scrf=(iefpcm

,solvent=dmso) geom=connectivity temperature=298

Zero-point correction= 0.364368 (Hartree/Particle)

Thermal correction to Energy= 0.398294

Thermal correction to Enthalpy= 0.399238

Thermal correction to Gibbs Free Energy= 0.294357

Sum of electronic and zero-point Energies= -2971.605692

Sum of electronic and thermal Energies= -2971.571767

Sum of electronic and thermal Enthalpies= -2971.570823

Sum of electronic and thermal Free Energies= -2971.675704

Title Card Required

0 1

C(PDBName=C1,ResName=UNK,ResNum=900) -3.69504600 -0.75609200 1.24235600

C(PDBName=C2,ResName=UNK,ResNum=900) -4.30734900 -0.97181300 0.00992800

N(PDBName=N1,ResName=UNK,ResNum=900) -3.46012600 -1.79036100 -0.71884200

N(PDBName=N2,ResName=UNK,ResNum=900) -2.50489000 -1.46564400 1.22410900

C(PDBName=C3,ResName=UNK,ResNum=900) -3.72456000 -2.23179800 -2.07513300

C(PDBName=C4,ResName=UNK,ResNum=900) -1.54332400 -1.49708500 2.31193600

C(PDBName=C5,ResName=UNK,ResNum=900) -2.36385700 -2.07450400 0.02384900

S(PDBName=S1,ResName=UNK,ResNum=900) -1.04067700 -3.03646700 -0.46898100

S(PDBName=S2,ResName=UNK,ResNum=900) 2.06784400 1.14939000 1.02694900

S(PDBName=S3,ResName=UNK,ResNum=900) 3.64412500 -0.37273500 -0.81445300

C(PDBName=C6,ResName=UNK,ResNum=900) 4.66991800 -1.46902100 0.29604300

C(PDBName=C7,ResName=UNK,ResNum=900) 1.72529300 2.58894800 -0.15370400

O(PDBName=O1,ResName=UNK,ResNum=900)	3.25123300	1.52436300	1.76567600
O(PDBName=O2,ResName=UNK,ResNum=900)	0.82663100	0.91590300	1.72987100
O(PDBName=O3,ResName=UNK,ResNum=900)	4.40615600	0.83944400	-1.01321200
O(PDBName=O4,ResName=UNK,ResNum=900)	3.30794400	-1.21932900	-1.93695200
F(PDBName=F1,ResName=UNK,ResNum=900)	5.05360000	-0.81532000	1.37726900
F(PDBName=F2,ResName=UNK,ResNum=900)	3.95314700	-2.52640800	0.65052500
F(PDBName=F3,ResName=UNK,ResNum=900)	5.73781000	-1.86929500	-0.37833300
F(PDBName=F4,ResName=UNK,ResNum=900)	1.38802500	2.14475200	-1.35191200
F(PDBName=F5,ResName=UNK,ResNum=900)	0.73547800	3.31278000	0.34406700
F(PDBName=F6,ResName=UNK,ResNum=900)	2.80439400	3.34455600	-0.25629100
N(PDBName=N3,ResName=UNK,ResNum=900)	2.28819600	-0.13154800	0.04388600
C(PDBName=C8,ResName=UNK,ResNum=900)	0.77953800	-1.43511200	-0.15381200
C(PDBName=C9,ResName=UNK,ResNum=900)	-1.90382200	0.74701700	-1.98291300
C(PDBName=C10,ResName=UNK,ResNum=900)	-3.00790000	1.46833800	-2.41439000
C(PDBName=C11,ResName=UNK,ResNum=900)	-3.60979600	2.35126700	-1.52589900
C(PDBName=C12,ResName=UNK,ResNum=900)	-3.07984000	2.47866400	-0.25062300
C(PDBName=C13,ResName=UNK,ResNum=900)	-1.97260100	1.71083500	0.08648000
N(PDBName=N4,ResName=UNK,ResNum=900)	-1.38962500	0.85823000	-0.75641500
C(PDBName=C14,ResName=UNK,ResNum=900)	-5.52725000	-0.39199200	-0.31182400
C(PDBName=C15,ResName=UNK,ResNum=900)	-4.27059200	0.05927200	2.20816700
C(PDBName=C16,ResName=UNK,ResNum=900)	-5.48592700	0.64508000	1.88827100
C(PDBName=C17,ResName=UNK,ResNum=900)	-6.10368300	0.42356100	0.65003500
H(PDBName=H1,ResName=UNK,ResNum=900)	-4.50530700	-2.99350600	-2.07765900
H(PDBName=H2,ResName=UNK,ResNum=900)	-4.04235700	-1.37478100	-2.66975400
H(PDBName=H3,ResName=UNK,ResNum=900)	-2.80914700	-2.64420400	-2.49241800
H(PDBName=H4,ResName=UNK,ResNum=900)	-0.83689900	-0.67021700	2.22070600
H(PDBName=H5,ResName=UNK,ResNum=900)	-1.00987400	-2.44555500	2.28075600
H(PDBName=H6,ResName=UNK,ResNum=900)	-2.08146200	-1.42432900	3.25561300
H(PDBName=H7,ResName=UNK,ResNum=900)	0.99502100	-1.87841100	0.80258400
H(PDBName=H8,ResName=UNK,ResNum=900)	1.22428800	-1.85836300	-1.04022200
H(PDBName=H9,ResName=UNK,ResNum=900)	0.05782400	-0.63070900	-0.25120000
H(PDBName=H10,ResName=UNK,ResNum=900)	-4.47849400	2.92674200	-1.82353500
H(PDBName=H11,ResName=UNK,ResNum=900)	-1.41038200	0.04138500	-2.64560500
H(PDBName=H12,ResName=UNK,ResNum=900)	-3.38680500	1.33609400	-3.42026700

H(PDBName=H13,ResName=UNK,ResNum=900)	-3.51844700	3.14845200	0.47797600
H(PDBName=H14,ResName=UNK,ResNum=900)	-1.52593400	1.77850300	1.07317500
H(PDBName=H15,ResName=UNK,ResNum=900)	-6.00129000	-0.56031800	-1.27026800
H(PDBName=H16,ResName=UNK,ResNum=900)	-7.05234600	0.90178600	0.43900400
H(PDBName=H17,ResName=UNK,ResNum=900)	-5.96684800	1.29234800	2.61146000
H(PDBName=H18,ResName=UNK,ResNum=900)	-3.79011900	0.24131000	3.16064600

TS-benzimidazolium-py-TFSI(2Step) -v3--

opt=(calcfc,ts,noeigen) freq=noraman wb97xd/6-311g(d,p) scrf=(iefpcm

,solvent=dmso) geom=connectivity temperature=363

Zero-point correction= 0.365478 (Hartree/Particle)

Thermal correction to Energy= 0.412565

Thermal correction to Enthalpy= 0.413715

Thermal correction to Gibbs Free Energy= 0.274224

Sum of electronic and zero-point Energies= -2971.615234

Sum of electronic and thermal Energies= -2971.568147

Sum of electronic and thermal Enthalpies= -2971.566997

Sum of electronic and thermal Free Energies= -2971.706488

Title Card Required

0 1

C(PDBName=C1,ResName=UNK,ResNum=900)	-2.83645200	-0.94147800	1.05829900
C(PDBName=C2,ResName=UNK,ResNum=900)	-3.16494600	-1.15597500	-0.27846800
N(PDBName=N1,ResName=UNK,ResNum=900)	-2.28772400	-2.11330100	-0.75736500
N(PDBName=N2,ResName=UNK,ResNum=900)	-1.78744000	-1.79720600	1.35119300
C(PDBName=C3,ResName=UNK,ResNum=900)	-2.31163400	-2.64393000	-2.10806600
C(PDBName=C4,ResName=UNK,ResNum=900)	-1.19095100	-1.95127300	2.66662400
C(PDBName=C5,ResName=UNK,ResNum=900)	-1.46055600	-2.49217500	0.23957000
S(PDBName=S1,ResName=UNK,ResNum=900)	-0.22733300	-3.67726700	0.11715000
S(PDBName=S2,ResName=UNK,ResNum=900)	0.29251000	0.70007200	-1.89421500
S(PDBName=S3,ResName=UNK,ResNum=900)	0.10609400	1.60643000	0.76046200
C(PDBName=C6,ResName=UNK,ResNum=900)	-1.41419500	2.68820000	0.42967500

C(PDBName=C7,ResName=UNK,ResNum=900)	2.09376100	0.28196400	-2.15313400
O(PDBName=O1,ResName=UNK,ResNum=900)	0.16978600	2.09140400	-2.29473900
O(PDBName=O2,ResName=UNK,ResNum=900)	-0.40526900	-0.31571900	-2.65480000
O(PDBName=O3,ResName=UNK,ResNum=900)	1.23226000	2.51443000	0.65143100
O(PDBName=O4,ResName=UNK,ResNum=900)	-0.14929100	0.98181000	2.04561200
F(PDBName=F1,ResName=UNK,ResNum=900)	-1.03876500	3.85015100	-0.08130700
F(PDBName=F2,ResName=UNK,ResNum=900)	-2.24744100	2.09616300	-0.41071500
F(PDBName=F3,ResName=UNK,ResNum=900)	-2.03859300	2.91002200	1.57843100
F(PDBName=F4,ResName=UNK,ResNum=900)	2.86810800	0.97398700	-1.33242700
F(PDBName=F5,ResName=UNK,ResNum=900)	2.29321000	-1.01582800	-1.94676700
F(PDBName=F6,ResName=UNK,ResNum=900)	2.42435600	0.56943500	-3.40672900
N(PDBName=N3,ResName=UNK,ResNum=900)	0.04981200	0.43547000	-0.33564100
C(PDBName=C8,ResName=UNK,ResNum=900)	1.57439200	-2.36667300	0.73751100
C(PDBName=C9,ResName=UNK,ResNum=900)	3.03521700	-0.17518700	2.05616800
C(PDBName=C10,ResName=UNK,ResNum=900)	4.05420200	0.71777700	2.34580500
C(PDBName=C11,ResName=UNK,ResNum=900)	5.27522700	0.56489300	1.70323300
C(PDBName=C12,ResName=UNK,ResNum=900)	5.43487400	-0.47376700	0.79487700
C(PDBName=C13,ResName=UNK,ResNum=900)	4.36362200	-1.32073400	0.56202500
N(PDBName=N4,ResName=UNK,ResNum=900)	3.19477500	-1.16916400	1.18352100
C(PDBName=C14,ResName=UNK,ResNum=900)	-4.21913300	-0.48821700	-0.88839700
C(PDBName=C15,ResName=UNK,ResNum=900)	-3.53763600	-0.03628000	1.84454500
C(PDBName=C16,ResName=UNK,ResNum=900)	-4.59033600	0.63177400	1.23879900
C(PDBName=C17,ResName=UNK,ResNum=900)	-4.92672000	0.40838000	-0.10329000
H(PDBName=H1,ResName=UNK,ResNum=900)	-1.36072200	-3.13135700	-2.30565800
H(PDBName=H2,ResName=UNK,ResNum=900)	-3.12632200	-3.36249000	-2.21183600
H(PDBName=H3,ResName=UNK,ResNum=900)	-2.43820000	-1.81894400	-2.80555300
H(PDBName=H4,ResName=UNK,ResNum=900)	-0.46006200	-1.16200700	2.83650200
H(PDBName=H5,ResName=UNK,ResNum=900)	-0.72138800	-2.93101900	2.72491300
H(PDBName=H6,ResName=UNK,ResNum=900)	-1.97968800	-1.89002800	3.41595700
H(PDBName=H7,ResName=UNK,ResNum=900)	2.17164900	-2.86248800	-0.00889900
H(PDBName=H8,ResName=UNK,ResNum=900)	1.03315500	-1.46923300	0.47816300
H(PDBName=H9,ResName=UNK,ResNum=900)	1.58045900	-2.73376200	1.74943800
H(PDBName=H10,ResName=UNK,ResNum=900)	6.09108100	1.24822200	1.90530000
H(PDBName=H11,ResName=UNK,ResNum=900)	2.05983900	-0.09136100	2.52283100

H(PDBName=H12,ResName=UNK,ResNum=900)	3.88596800	1.51579100	3.05675500
H(PDBName=H13,ResName=UNK,ResNum=900)	6.36945100	-0.62695400	0.27153600
H(PDBName=H14,ResName=UNK,ResNum=900)	4.43521200	-2.14045300	-0.14441800
H(PDBName=H15,ResName=UNK,ResNum=900)	-4.47680900	-0.66093100	-1.92535900
H(PDBName=H16,ResName=UNK,ResNum=900)	-5.75566600	0.95273900	-0.53884200
H(PDBName=H17,ResName=UNK,ResNum=900)	-5.16193300	1.34829900	1.81571300
H(PDBName=H18,ResName=UNK,ResNum=900)	-3.27248200	0.14305500	2.87807200

TS-benzimidazolium-py-TFSI(2Step)-v4--

 # opt=(calcfc,ts,noeigen) freq=noraman wb97xd/6-311g(d,p) scrf=(iefpcm

 ,solvent=dmso) geom=connectivity temperature=363

Zero-point correction= 0.273109 (Hartree/Particle)

Thermal correction to Energy= 0.312711

Thermal correction to Enthalpy= 0.313860

Thermal correction to Gibbs Free Energy= 0.188379

Sum of electronic and zero-point Energies= -2723.417363

Sum of electronic and thermal Energies= -2723.377761

Sum of electronic and thermal Enthalpies= -2723.376612

Sum of electronic and thermal Free Energies= -2723.502093

Title Card Required

0 1

C(PDBName=C1,ResName=UNK,ResNum=900)	-3.72839200	0.09765700	0.56702200
C(PDBName=C2,ResName=UNK,ResNum=900)	-3.98810500	-0.53889000	-0.64390200
N(PDBName=N1,ResName=UNK,ResNum=900)	-3.22231700	-1.69395500	-0.66038400
N(PDBName=N2,ResName=UNK,ResNum=900)	-2.82240900	-0.69949900	1.24863800
C(PDBName=C3,ResName=UNK,ResNum=900)	-3.20122100	-2.63799500	-1.76408100
C(PDBName=C4,ResName=UNK,ResNum=900)	-2.29756200	-0.39100800	2.56859300
C(PDBName=C5,ResName=UNK,ResNum=900)	-2.51599300	-1.77973200	0.49080300
S(PDBName=S1,ResName=UNK,ResNum=900)	-1.37360000	-2.99282400	0.88681500
S(PDBName=S2,ResName=UNK,ResNum=900)	1.49626400	1.46984000	0.76576500
S(PDBName=S3,ResName=UNK,ResNum=900)	2.96181000	-0.30427200	-0.98761700

C(PDBName=C6,ResName=UNK,ResNum=900)	4.33549000	-0.90623600	0.12240600
C(PDBName=C7,ResName=UNK,ResNum=900)	0.25517100	2.21724100	-0.41260100
O(PDBName=O1,ResName=UNK,ResNum=900)	2.63668100	2.35196900	0.77450900
O(PDBName=O2,ResName=UNK,ResNum=900)	0.74350700	1.23295700	1.97793400
O(PDBName=O3,ResName=UNK,ResNum=900)	3.46931100	0.86223400	-1.66800400
O(PDBName=O4,ResName=UNK,ResNum=900)	2.54586600	-1.46882300	-1.73373000
F(PDBName=F1,ResName=UNK,ResNum=900)	4.69303600	0.04528600	0.96779600
F(PDBName=F2,ResName=UNK,ResNum=900)	3.92105100	-1.96599700	0.80129700
F(PDBName=F3,ResName=UNK,ResNum=900)	5.37276600	-1.24314900	-0.62814200
F(PDBName=F4,ResName=UNK,ResNum=900)	-0.81642400	1.43570200	-0.47862400
F(PDBName=F5,ResName=UNK,ResNum=900)	-0.10062200	3.40717400	0.04277000
F(PDBName=F6,ResName=UNK,ResNum=900)	0.77953400	2.34211100	-1.61878500
N(PDBName=N3,ResName=UNK,ResNum=900)	1.78459900	0.01639400	0.09268600
C(PDBName=C8,ResName=UNK,ResNum=900)	0.43084600	-1.40723800	0.44729500
C(PDBName=C14,ResName=UNK,ResNum=900)	-4.86587600	-0.00407000	-1.57834200
C(PDBName=C15,ResName=UNK,ResNum=900)	-4.32782800	1.30580900	0.90010100
C(PDBName=C16,ResName=UNK,ResNum=900)	-5.20366300	1.84379200	-0.03075800
C(PDBName=C17,ResName=UNK,ResNum=900)	-5.46845200	1.20025100	-1.24811100
H(PDBName=H1,ResName=UNK,ResNum=900)	-2.44673500	-3.39297100	-1.56009400
H(PDBName=H2,ResName=UNK,ResNum=900)	-4.17855300	-3.11176500	-1.86243900
H(PDBName=H3,ResName=UNK,ResNum=900)	-2.95235700	-2.11026100	-2.68505600
H(PDBName=H4,ResName=UNK,ResNum=900)	-1.56747700	0.41731700	2.50330700
H(PDBName=H5,ResName=UNK,ResNum=900)	-1.82492600	-1.28372800	2.97179700
H(PDBName=H6,ResName=UNK,ResNum=900)	-3.12169900	-0.09621000	3.21769400
H(PDBName=H7,ResName=UNK,ResNum=900)	0.18968800	-0.94282600	1.38643400
H(PDBName=H8,ResName=UNK,ResNum=900)	1.15130100	-2.20858200	0.42786300
H(PDBName=H9,ResName=UNK,ResNum=900)	-0.09422800	-1.15459100	-0.45614800
H(PDBName=H15,ResName=UNK,ResNum=900)	-5.06743100	-0.50034900	-2.51891700
H(PDBName=H16,ResName=UNK,ResNum=900)	-6.15900700	1.65493100	-1.94770400
H(PDBName=H17,ResName=UNK,ResNum=900)	-5.69296000	2.78529400	0.18657200
H(PDBName=H18,ResName=UNK,ResNum=900)	-4.11980200	1.80490700	1.83765100

14. TS-benzimidazolium-py-TFSI-back

\# opt=(calcfc,ts,noeigen) freq=noraman wb97xd/6-311g(d,p) scrf=(iefpcm
,solvent=dmso) geom=connectivity temperature=363

Zero-point correction= 0.364497 (Hartree/Particle)

Thermal correction to Energy= 0.412243

Thermal correction to Enthalpy= 0.413392

Thermal correction to Gibbs Free Energy= 0.270452

Sum of electronic and zero-point Energies= -2971.609434

Sum of electronic and thermal Energies= -2971.561689

Sum of electronic and thermal Enthalpies= -2971.560539

Sum of electronic and thermal Free Energies= -2971.703479

Title Card Required

0 1

C(PDBName=C1,ResName=UNK,ResNum=900)	-1.36536300	3.07593600	0.97612800
C(PDBName=C2,ResName=UNK,ResNum=900)	-2.12749000	2.25734700	0.14575500
N(PDBName=N1,ResName=UNK,ResNum=900)	-1.46156900	2.20229400	-1.06964600
N(PDBName=N2,ResName=UNK,ResNum=900)	-0.27353000	3.49396700	0.23725700
C(PDBName=C3,ResName=UNK,ResNum=900)	-1.85025900	1.40822700	-2.22454400
C(PDBName=C4,ResName=UNK,ResNum=900)	0.79005500	4.33209300	0.75865400
C(PDBName=C5,ResName=UNK,ResNum=900)	-0.33819400	2.94658800	-0.99473200
S(PDBName=S1,ResName=UNK,ResNum=900)	0.80232600	3.15447500	-2.26006100
S(PDBName=S2,ResName=UNK,ResNum=900)	0.23211400	-0.71348500	0.31477400
C(PDBName=C6,ResName=UNK,ResNum=900)	1.34764300	-1.84548300	1.33929000
O(PDBName=O2,ResName=UNK,ResNum=900)	0.47691800	0.60596900	0.86480000
F(PDBName=F1,ResName=UNK,ResNum=900)	1.80136100	-2.84655900	0.60390000
F(PDBName=F2,ResName=UNK,ResNum=900)	2.38016000	-1.13479500	1.77649000
F(PDBName=F3,ResName=UNK,ResNum=900)	0.69182000	-2.33110300	2.38444000
C(PDBName=C7,ResName=UNK,ResNum=900)	2.40084000	1.70107100	-1.44368600
C(PDBName=C8,ResName=UNK,ResNum=900)	3.87451300	-0.87722800	-1.17047700
C(PDBName=C9,ResName=UNK,ResNum=900)	4.78473200	-1.79793400	-0.67596600
C(PDBName=C10,ResName=UNK,ResNum=900)	5.65073300	-1.40015200	0.33333800
C(PDBName=C11,ResName=UNK,ResNum=900)	5.57709200	-0.09873800	0.81341900

C(PDBName=C12,ResName=UNK,ResNum=900)	4.63741200	0.75759100	0.26319800
N(PDBName=N3,ResName=UNK,ResNum=900)	3.81070900	0.37000500	-0.70729800
C(PDBName=C13,ResName=UNK,ResNum=900)	-3.29739200	1.65919700	0.59760700
C(PDBName=C14,ResName=UNK,ResNum=900)	-1.73036600	3.33580300	2.29107900
C(PDBName=C15,ResName=UNK,ResNum=900)	-2.89791400	2.74197200	2.74155300
C(PDBName=C16,ResName=UNK,ResNum=900)	-3.66773500	1.91828900	1.90703200
O(PDBName=O3,ResName=UNK,ResNum=900)	0.60966600	-0.96445400	-1.06763600
H(PDBName=H1,ResName=UNK,ResNum=900)	-2.84674100	1.01163200	-2.05129600
H(PDBName=H2,ResName=UNK,ResNum=900)	-1.86146300	2.03206700	-3.11646400
H(PDBName=H3,ResName=UNK,ResNum=900)	-1.14920100	0.58382100	-2.35752900
H(PDBName=H4,ResName=UNK,ResNum=900)	1.35211600	3.78386300	1.51642300
H(PDBName=H5,ResName=UNK,ResNum=900)	1.44870400	4.60972000	-0.06075800
H(PDBName=H6,ResName=UNK,ResNum=900)	0.35839700	5.23065000	1.19947600
H(PDBName=H7,ResName=UNK,ResNum=900)	1.91917800	0.86847000	-1.92588200
H(PDBName=H8,ResName=UNK,ResNum=900)	2.14592300	1.92325700	-0.42139000
H(PDBName=H9,ResName=UNK,ResNum=900)	6.37266600	-2.09644300	0.74249900
H(PDBName=H10,ResName=UNK,ResNum=900)	3.16345100	-1.14202400	-1.94447000
H(PDBName=H11,ResName=UNK,ResNum=900)	4.80449000	-2.80392100	-1.07361800
H(PDBName=H12,ResName=UNK,ResNum=900)	6.23226200	0.24980100	1.60087000
H(PDBName=H13,ResName=UNK,ResNum=900)	4.53388600	1.78214100	0.60455500
H(PDBName=H14,ResName=UNK,ResNum=900)	-3.88808600	1.00778600	-0.03096100
H(PDBName=H15,ResName=UNK,ResNum=900)	-4.57065400	1.46591000	2.29858700
H(PDBName=H16,ResName=UNK,ResNum=900)	-3.22117900	2.91347600	3.76105600
H(PDBName=H17,ResName=UNK,ResNum=900)	-1.12800700	3.96586400	2.93286000
H(PDBName=H18,ResName=UNK,ResNum=900)	3.18791900	2.24558000	-1.93824300
N	-1.27032200	-1.10838300	0.68607700
S	-1.95133000	-2.50635300	0.33005600
O	-1.01689600	-3.58668100	0.06223900
O	-3.04298800	-2.73068100	1.25488400
C	-2.80958400	-2.22374300	-1.30214900
F	-3.44350500	-3.33730900	-1.64864200
F	-1.94020900	-1.91143300	-2.25163300
F	-3.70144700	-1.24205300	-1.19389400

15. bimidazolium-I

----

opt=calcfc freq=noraman wb97xd/gen scrf=(iefpcm,solvent=dmso) geom=c
onnectivity pseudo=read temperature=363

Zero-point correction= 0.218518 (Hartree/Particle)
Thermal correction to Energy= 0.239890
Thermal correction to Enthalpy= 0.241040
Thermal correction to Gibbs Free Energy= 0.157491
Sum of electronic and zero-point Energies= -907.781325
Sum of electronic and thermal Energies= -907.759952
Sum of electronic and thermal Enthalpies= -907.758803
Sum of electronic and thermal Free Energies= -907.842352

Title Card Required

0 1

C	-1.81561200	0.08084000	0.64048700
C	-1.71084600	0.23646000	-0.73959400
N	-0.75858900	-0.66902700	-1.17141000
N	-0.91033200	-0.90054600	1.00014200
C	-0.27346700	-0.75709400	-2.54004500
H	0.38619400	0.08928500	-2.73657800
H	0.27401200	-1.68731200	-2.66304900
H	-1.12561700	-0.74284200	-3.21730900
C	-0.63841300	-1.29929500	2.37242900
H	-1.52315000	-1.77077300	2.79899700
H	0.19444600	-1.99739000	2.37761200
H	-0.36978100	-0.41288400	2.94692500
C	-0.27601500	-1.31952200	-0.10422000
C	-2.47270100	1.17481700	-1.43082200
C	-2.69417500	0.84605500	1.40257300
C	-3.45377800	1.77542700	0.71692900
C	-3.34365200	1.93795900	-0.67574200

H	-2.38261100	1.30667300	-2.50090600
H	-3.95607600	2.68297700	-1.16832500
H	-4.14900000	2.39781800	1.26648200
H	-2.77637300	0.72134700	2.47428600
I	2.11661700	1.35696700	0.12405400
S	0.99321400	-2.52453400	-0.14876500
C	-0.04095200	-4.02943500	-0.05275000
H	0.66197500	-4.85977900	-0.10822400
H	-0.57755500	-4.07087800	0.89351800
H	-0.72844800	-4.07318000	-0.89466200

16. bimidazolium-I-in

----

opt=calcfc freq=noraman wb97xd/gen scrf=(iefpcm,solvent=dmso) geom=c
onnectivity pseudo=read temperature=363

Zero-point correction= 0.218719 (Hartree/Particle)
Thermal correction to Energy= 0.239873
Thermal correction to Enthalpy= 0.241023
Thermal correction to Gibbs Free Energy= 0.158875
Sum of electronic and zero-point Energies= -907.781582
Sum of electronic and thermal Energies= -907.760427
Sum of electronic and thermal Enthalpies= -907.759278
Sum of electronic and thermal Free Energies= -907.841425

Title Card Required

0 1

C	-1.65998200	-0.12878600	0.76903000
C	-1.79101500	-0.52306800	-0.55943200
N	-0.82224600	-1.48676900	-0.78417500
N	-0.60499200	-0.84957100	1.29961400
C	-0.63119800	-2.19418700	-2.04131500
H	-0.00898800	-1.60112400	-2.71182400

H	-0.16265900	-3.15561700	-1.84170500
H	-1.60533700	-2.36430500	-2.49540300
C	-0.06387500	-0.62579000	2.63183400
H	-0.81244700	-0.89002800	3.37774400
H	0.82052500	-1.24246200	2.76298600
H	0.20939000	0.42627000	2.71724400
C	-0.10951600	-1.64491900	0.34201000
C	-2.75219000	0.03336200	-1.39777100
C	-2.48535000	0.83926600	1.33304600
C	-3.44350300	1.39107200	0.50311400
C	-3.57494300	0.99420100	-0.83925500
H	-2.84988900	-0.26338300	-2.43364900
H	-4.33847700	1.45646800	-1.45248600
H	-4.10913000	2.15040600	0.89436300
H	-2.37780100	1.14817000	2.36452000
I	1.55865800	1.80162200	-0.23315800
S	1.19333300	-2.78606100	0.57864500
C	2.42540800	-2.15373200	-0.61196700
H	3.35461200	-2.65260500	-0.33926500
H	2.15921500	-2.41528800	-1.63374700
H	2.52645600	-1.07564300	-0.49352400

17. TS-bimidazolium-I-py

----

opt=(calcfc,ts,noeigen) freq=noraman wb97xd/gen scrf=(iefpcm,solvent
=dmso) geom=connectivity pseudo=read temperature=363

Zero-point correction= 0.308571 (Hartree/Particle)

Thermal correction to Energy= 0.337884

Thermal correction to Enthalpy= 0.339034

Thermal correction to Gibbs Free Energy= 0.234511

Sum of electronic and zero-point Energies= -1155.915774

Sum of electronic and thermal Energies= -1155.886461

Sum of electronic and thermal Enthalpies= -1155.885311

Sum of electronic and thermal Free Energies= -1155.989834

Title Card Required

0 1

C	-0.92566600	1.50335800	-0.69038800
C	-1.18522100	1.60808000	0.67349200
N	-0.69698300	0.45404900	1.26132200
N	-0.30000300	0.28383400	-0.88367100
C	-0.85788400	0.12751800	2.66626400
H	-1.90189000	-0.12389100	2.86199200
H	-0.22515900	-0.72391500	2.90268100
H	-0.55973700	0.98440200	3.26975200
C	0.04799000	-0.24966300	-2.18788900
H	0.80651000	0.37821700	-2.65636300
H	0.42953900	-1.25968700	-2.06023800
H	-0.84630700	-0.27881100	-2.81179300
C	-0.18345900	-0.34837600	0.30496400
S	0.54405600	-1.88200800	0.56237300
C	2.73036800	-1.13730000	0.31811700
H	3.09595600	-2.10184600	0.62899100
H	2.58627900	-0.93147000	-0.72794400
H	2.57086100	-0.36688100	1.05343700
C	5.11177200	0.61576500	0.69596500
C	6.41406800	1.06486900	0.54238700
C	7.28334800	0.32452500	-0.24825000
C	6.82187300	-0.83578700	-0.85678600
C	5.50483000	-1.21260900	-0.65134600
N	4.67571300	-0.49828200	0.10958000
H	8.30763600	0.64759300	-0.38881000
H	4.39528100	1.15915200	1.30268300
H	6.73346800	1.97431600	1.03386800
H	7.46603800	-1.44125100	-1.48053100

H	5.09352600	-2.10830400	-1.10433800
C	-1.83411900	2.71259100	1.21060000
C	-1.29884300	2.49826900	-1.58498700
C	-1.94385700	3.60386600	-1.05203300
C	-2.20676400	3.70902600	0.32151700
H	-2.04480900	2.79042700	2.26940500
H	-2.71570300	4.58844500	0.69671900
H	-2.25566900	4.40320900	-1.71310500
H	-1.10170900	2.41207000	-2.64582900
I	-3.87095700	-1.23531400	-0.18130000

---

18. TS-bimidazolium-I-v2

----

opt=(calcfc,ts,noeigen) freq=noraman wb97xd/gen scrf=(iefpcm,solvent

=dmso) geom=connectivity pseudo=read temperature=363

Zero-point correction= 0.309069 (Hartree/Particle)

Thermal correction to Energy= 0.337902

Thermal correction to Enthalpy= 0.339051

Thermal correction to Gibbs Free Energy= 0.237766

Sum of electronic and zero-point Energies= -1155.920688

Sum of electronic and thermal Energies= -1155.891856

Sum of electronic and thermal Enthalpies= -1155.890706

Sum of electronic and thermal Free Energies= -1155.991992

Title Card Required

0 1

C(PDBName=C,ResName=UNK,ResNum=0)	2.50505700	0.82432000	1.12488300
C(PDBName=C,ResName=UNK,ResNum=0)	3.10695300	0.46232700	-0.07736700
N(PDBName=N,ResName=UNK,ResNum=0)	2.33991100	1.01101600	-1.09088000
N(PDBName=N,ResName=UNK,ResNum=0)	1.39665700	1.58918100	0.79789500
C(PDBName=C,ResName=UNK,ResNum=0)	2.55149100	0.73573800	-2.50002900

C(PDBName=C,ResName=UNK,ResNum=0)	0.49322800	2.18683700	1.76430800
C(PDBName=C,ResName=UNK,ResNum=0)	1.29616100	1.67315800	-0.54792500
S(PDBName=S,ResName=UNK,ResNum=0)	0.08061800	2.51165800	-1.42077900
C(PDBName=C,ResName=UNK,ResNum=0)	-1.83564400	1.49841800	-0.61727200
C(PDBName=C,ResName=UNK,ResNum=0)	-4.66151000	1.38008800	0.27101900
C(PDBName=C,ResName=UNK,ResNum=0)	-5.86850600	0.85002000	0.69706600
C(PDBName=C,ResName=UNK,ResNum=0)	-5.95945700	-0.52317600	0.88839600
C(PDBName=C,ResName=UNK,ResNum=0)	-4.84338700	-1.31350800	0.64749300
C(PDBName=C,ResName=UNK,ResNum=0)	-3.67262700	-0.70289500	0.22389300
N(PDBName=N,ResName=UNK,ResNum=0)	-3.59339100	0.61563900	0.04286200
C(PDBName=C,ResName=UNK,ResNum=0)	4.24888800	-0.32721900	-0.11425800
C(PDBName=C,ResName=UNK,ResNum=0)	3.01539200	0.41216800	2.34924700
C(PDBName=C,ResName=UNK,ResNum=0)	4.15743600	-0.37379100	2.31679100
C(PDBName=C,ResName=UNK,ResNum=0)	4.76345000	-0.73761600	1.10610900
H(PDBName=H,ResName=UNK,ResNum=0)	3.59242900	0.93567700	-2.75291600
H(PDBName=H,ResName=UNK,ResNum=0)	2.30894300	-0.31030400	-2.69681600
H(PDBName=H,ResName=UNK,ResNum=0)	1.90458400	1.38010300	-3.08860900
H(PDBName=H,ResName=UNK,ResNum=0)	0.02378400	3.05999100	1.31568900
H(PDBName=H,ResName=UNK,ResNum=0)	-0.26773500	1.46710500	2.06982200
H(PDBName=H,ResName=UNK,ResNum=0)	1.06737400	2.50191500	2.63426500
H(PDBName=H,ResName=UNK,ResNum=0)	-1.31508400	0.56738600	-0.43820100
H(PDBName=H,ResName=UNK,ResNum=0)	-1.95443500	2.22663100	0.16564300
H(PDBName=H,ResName=UNK,ResNum=0)	-2.31019900	1.66279300	-1.57037300
H(PDBName=H,ResName=UNK,ResNum=0)	-6.88802800	-0.97071600	1.22154900
H(PDBName=H,ResName=UNK,ResNum=0)	-4.53708400	2.44509100	0.10537100
H(PDBName=H,ResName=UNK,ResNum=0)	-6.71370200	1.50232000	0.87355700
H(PDBName=H,ResName=UNK,ResNum=0)	-4.87271000	-2.38652200	0.78519800
H(PDBName=H,ResName=UNK,ResNum=0)	-2.76813200	-1.27128500	0.02424700
H(PDBName=H,ResName=UNK,ResNum=0)	4.71024500	-0.61680000	-1.04955500
H(PDBName=H,ResName=UNK,ResNum=0)	5.65142600	-1.35770200	1.12414700
H(PDBName=H,ResName=UNK,ResNum=0)	4.58783800	-0.71975300	3.24859700
H(PDBName=H,ResName=UNK,ResNum=0)	2.54186400	0.68081300	3.28469200
I	0.13518800	-2.07100700	-0.48994500

19. TS-benzimidazolium-py-I

----

opt=(calcfc,ts,noeigen) freq=noraman wb97xd/gen scrf=(iefpcm,solvent

=dmso) geom=connectivity pseudo=read temperature=363

Zero-point correction= 0.127534 (Hartree/Particle)

Thermal correction to Energy= 0.139251

Thermal correction to Enthalpy= 0.140400

Thermal correction to Gibbs Free Energy= 0.080039

Sum of electronic and zero-point Energies= -299.414339

Sum of electronic and thermal Energies= -299.402622

Sum of electronic and thermal Enthalpies= -299.401472

Sum of electronic and thermal Free Energies= -299.461833

Title Card Required

0 1

C(PDBName=C8,ResName=UNK,ResNum=900)	0.10384400	0.00461700	-0.03539300
C(PDBName=C9,ResName=UNK,ResNum=900)	-2.69366500	-1.14429900	-0.02746200
C(PDBName=C10,ResName=UNK,ResNum=900)	-4.07789800	-1.20406800	0.01830400
C(PDBName=C11,ResName=UNK,ResNum=900)	-4.79336300	-0.01370700	0.04232100
C(PDBName=C12,ResName=UNK,ResNum=900)	-4.10105900	1.18996400	0.01917800
C(PDBName=C13,ResName=UNK,ResNum=900)	-2.71564500	1.15718800	-0.02678100
N(PDBName=N4,ResName=UNK,ResNum=900)	-2.03327100	0.01304100	-0.05006000
H(PDBName=H7,ResName=UNK,ResNum=900)	0.07872300	1.03680100	-0.33846900
H(PDBName=H8,ResName=UNK,ResNum=900)	0.04772000	-0.24936200	1.00814300
H(PDBName=H9,ResName=UNK,ResNum=900)	0.07392100	-0.77061200	-0.78122400
H(PDBName=H10,ResName=UNK,ResNum=900)	-5.87596900	-0.02422900	0.08044200
H(PDBName=H11,ResName=UNK,ResNum=900)	-2.09035100	-2.04588600	-0.04481300
H(PDBName=H12,ResName=UNK,ResNum=900)	-4.57775200	-2.16364200	0.03604400
H(PDBName=H13,ResName=UNK,ResNum=900)	-4.61888100	2.13997800	0.03773700
H(PDBName=H14,ResName=UNK,ResNum=900)	-2.13214500	2.07167300	-0.04357000
I	2.69800700	-0.00045600	0.00858700

20. TS-b-py-I-conformr2(2Step)

----

opt=(calcfc,ts,noeigen) freq=noraman wb97xd/gen scrf=(iefpcm,solvent
=dmso) geom=connectivity pseudo=read temperature=363

Zero-point correction= 0.307616 (Hartree/Particle)
Thermal correction to Energy= 0.336703
Thermal correction to Enthalpy= 0.337853
Thermal correction to Gibbs Free Energy= 0.235744
Sum of electronic and zero-point Energies= -1155.924359
Sum of electronic and thermal Energies= -1155.895271
Sum of electronic and thermal Enthalpies= -1155.894122
Sum of electronic and thermal Free Energies= -1155.996230

Title Card Required

0 1

C(PDBName=C,ResName=UNK,ResNum=0)	-2.97055900	0.66448800	-0.06386100
C(PDBName=C,ResName=UNK,ResNum=0)	-2.35577000	0.64345300	1.18587200
N(PDBName=N,ResName=UNK,ResNum=0)	-1.17481500	1.35688000	1.05946800
N(PDBName=N,ResName=UNK,ResNum=0)	-2.13315700	1.37382900	-0.90907000
C(PDBName=C,ResName=UNK,ResNum=0)	-0.20132900	1.53490600	2.11991400
C(PDBName=C,ResName=UNK,ResNum=0)	-2.40392800	1.60874100	-2.31484100
C(PDBName=C,ResName=UNK,ResNum=0)	-1.04140500	1.78177700	-0.21908100
S(PDBName=S,ResName=UNK,ResNum=0)	0.26707300	2.67551700	-0.85656100
C(PDBName=C,ResName=UNK,ResNum=0)	2.12176900	1.19051600	-0.31382900
C(PDBName=C,ResName=UNK,ResNum=0)	-0.37165300	-1.23623700	-1.60242400
C(PDBName=C,ResName=UNK,ResNum=0)	-1.41511900	-1.97344600	-2.14507000
C(PDBName=C,ResName=UNK,ResNum=0)	-2.15099800	-2.79727400	-1.30287100
C(PDBName=C,ResName=UNK,ResNum=0)	-1.80793900	-2.85316000	0.04064200
C(PDBName=C,ResName=UNK,ResNum=0)	-0.74540900	-2.07757700	0.48559600
N(PDBName=N,ResName=UNK,ResNum=0)	-0.03473800	-1.27818500	-0.31179400
C(PDBName=C,ResName=UNK,ResNum=0)	-2.92162800	-0.02424500	2.26426700
C(PDBName=C,ResName=UNK,ResNum=0)	-4.18418700	0.03023900	-0.29280700

C(PDBName=C,ResName=UNK,ResNum=0)	-4.75216900	-0.63643800	0.78207500
C(PDBName=C,ResName=UNK,ResNum=0)	-4.13127000	-0.66349600	2.03802800
H(PDBName=H,ResName=UNK,ResNum=0)	0.37532700	2.43560500	1.92014700
H(PDBName=H,ResName=UNK,ResNum=0)	-0.72721600	1.65011100	3.06627200
H(PDBName=H,ResName=UNK,ResNum=0)	0.46491100	0.67140900	2.17046000
H(PDBName=H,ResName=UNK,ResNum=0)	-1.49024500	1.95466000	-2.79216900
H(PDBName=H,ResName=UNK,ResNum=0)	-2.72411200	0.67304500	-2.77394100
H(PDBName=H,ResName=UNK,ResNum=0)	-3.18487800	2.36204700	-2.42745300
H(PDBName=H,ResName=UNK,ResNum=0)	2.41627000	1.83126800	0.49883300
H(PDBName=H,ResName=UNK,ResNum=0)	2.51807700	1.37798300	-1.29752500
H(PDBName=H,ResName=UNK,ResNum=0)	1.43895500	0.36551200	-0.15340500
H(PDBName=H,ResName=UNK,ResNum=0)	-2.97799500	-3.38273300	-1.68702600
H(PDBName=H,ResName=UNK,ResNum=0)	0.21906200	-0.57284600	-2.22901400
H(PDBName=H,ResName=UNK,ResNum=0)	-1.64249600	-1.89774700	-3.20115400
H(PDBName=H,ResName=UNK,ResNum=0)	-2.35616800	-3.47503600	0.73671800
H(PDBName=H,ResName=UNK,ResNum=0)	-0.45588000	-2.09190400	1.53197800
H(PDBName=H,ResName=UNK,ResNum=0)	-2.43862500	-0.05655700	3.23232200
H(PDBName=H,ResName=UNK,ResNum=0)	-4.60576500	-1.19781000	2.85204700
H(PDBName=H,ResName=UNK,ResNum=0)	-5.69689100	-1.14875500	0.64721200
H(PDBName=H,ResName=UNK,ResNum=0)	-4.66129900	0.04894200	-1.26414800
I	4.16310100	-0.44672900	0.22707400

21. **TS-benzimidazolium-py-I (2Step)** --

\# opt=(calcfc,ts,noeigen) freq=noraman wb97xd/gen scrf=(iefpcm,solvent
=dmso) geom=connectivity pseudo=read temperature=363

Zero-point correction= 0.216475 (Hartree/Particle)
Thermal correction to Energy= 0.237373
Thermal correction to Enthalpy= 0.238523
Thermal correction to Gibbs Free Energy= 0.156639
Sum of electronic and zero-point Energies= -907.737671
Sum of electronic and thermal Energies= -907.716773
Sum of electronic and thermal Enthalpies= -907.715623
Sum of electronic and thermal Free Energies= -907.797507

Title Card Required

0 1

C(PDBName=C,ResName=UNK,ResNum=0)	-3.13996800	0.11978200	-0.47480800
C(PDBName=C,ResName=UNK,ResNum=0)	-2.61656700	-0.74968500	0.47968900
N(PDBName=N,ResName=UNK,ResNum=0)	-1.54913100	-0.09182900	1.07218700
N(PDBName=N,ResName=UNK,ResNum=0)	-2.36373400	1.26702100	-0.44066600
C(PDBName=C,ResName=UNK,ResNum=0)	-0.74138400	-0.63780700	2.14902300
C(PDBName=C,ResName=UNK,ResNum=0)	-2.57971400	2.41902600	-1.29785400
C(PDBName=C,ResName=UNK,ResNum=0)	-1.40218400	1.12933500	0.50256900
S(PDBName=S,ResName=UNK,ResNum=0)	-0.20397600	2.28305100	0.89378800
C(PDBName=C,ResName=UNK,ResNum=0)	1.73172600	0.93141000	0.29579300
C(PDBName=C,ResName=UNK,ResNum=0)	-3.16275400	-2.00869800	0.69571100
C(PDBName=C,ResName=UNK,ResNum=0)	-4.23842800	-0.22308100	-1.25275400
C(PDBName=C,ResName=UNK,ResNum=0)	-4.78788700	-1.47802600	-1.03745000
C(PDBName=C,ResName=UNK,ResNum=0)	-4.25919900	-2.35514000	-0.08035900
H(PDBName=H,ResName=UNK,ResNum=0)	-0.25873800	0.18354600	2.67415300
H(PDBName=H,ResName=UNK,ResNum=0)	-1.39206800	-1.17256700	2.83970000
H(PDBName=H,ResName=UNK,ResNum=0)	0.01234300	-1.32095400	1.75407800
H(PDBName=H,ResName=UNK,ResNum=0)	-1.75379700	3.11272200	-1.16426100
H(PDBName=H,ResName=UNK,ResNum=0)	-2.61802000	2.09118700	-2.33693700
H(PDBName=H,ResName=UNK,ResNum=0)	-3.51691900	2.90897800	-1.03101900
H(PDBName=H,ResName=UNK,ResNum=0)	1.89626700	0.68768600	1.32952800
H(PDBName=H,ResName=UNK,ResNum=0)	2.18700800	1.80866400	-0.12973900
H(PDBName=H,ResName=UNK,ResNum=0)	1.10980600	0.30543600	-0.32035300
H(PDBName=H,ResName=UNK,ResNum=0)	-2.75372200	-2.68958000	1.43114900
H(PDBName=H,ResName=UNK,ResNum=0)	-4.71676700	-3.32721600	0.05652300
H(PDBName=H,ResName=UNK,ResNum=0)	-5.64524400	-1.78749600	-1.62249600
H(PDBName=H,ResName=UNK,ResNum=0)	-4.64598700	0.45604000	-1.99058300
I	3.84769200	-0.54517600	-0.37902600

22. TS-benzimidazoilum-py-I-V3

----

opt=(calcfc,ts,noeigen) freq=noraman wb97xd/gen scrf=(iefpcm,solvent
=dmso) geom=connectivity pseudo=read temperature=363

Zero-point correction= 0.307657 (Hartree/Particle)
Thermal correction to Energy= 0.336600
Thermal correction to Enthalpy= 0.337749
Thermal correction to Gibbs Free Energy= 0.238139
Sum of electronic and zero-point Energies= -1155.874441
Sum of electronic and thermal Energies= -1155.845498
Sum of electronic and thermal Enthalpies= -1155.844349
Sum of electronic and thermal Free Energies= -1155.943959

Title Card Required

0 1

C(PDBName=C1,ResName=UNK,ResNum=900)	2.71712500	0.89592400	-0.42389300
C(PDBName=C2,ResName=UNK,ResNum=900)	1.53554000	1.45083700	-0.91130000
N(PDBName=N1,ResName=UNK,ResNum=900)	0.84174400	0.42758800	-1.53506200
N(PDBName=N2,ResName=UNK,ResNum=900)	2.68844000	-0.45036200	-0.74885300
C(PDBName=C3,ResName=UNK,ResNum=900)	-0.45484400	0.58164200	-2.16945700
C(PDBName=C4,ResName=UNK,ResNum=900)	3.76240100	-1.38724300	-0.47108200
C(PDBName=C5,ResName=UNK,ResNum=900)	1.53882700	-0.72643000	-1.41317100
S(PDBName=S1,ResName=UNK,ResNum=900)	1.02242900	-2.26493100	-1.92870100
C(PDBName=C7,ResName=UNK,ResNum=900)	-1.28150600	-2.56507100	-0.55769200
C(PDBName=C8,ResName=UNK,ResNum=900)	-0.07507700	-0.38968700	1.40157400
C(PDBName=C9,ResName=UNK,ResNum=900)	0.76636200	0.23472700	2.31110600
C(PDBName=C10,ResName=UNK,ResNum=900)	1.81433200	-0.49394300	2.85588700
C(PDBName=C11,ResName=UNK,ResNum=900)	1.98311200	-1.81997200	2.47534700
C(PDBName=C12,ResName=UNK,ResNum=900)	1.11187800	-2.35831000	1.54104200
N(PDBName=N3,ResName=UNK,ResNum=900)	0.11107700	-1.65198700	1.01993400
C(PDBName=C13,ResName=UNK,ResNum=900)	1.21545500	2.78666500	-0.70447500
C(PDBName=C14,ResName=UNK,ResNum=900)	3.64502800	1.65618000	0.27666400
C(PDBName=C15,ResName=UNK,ResNum=900)	3.33048200	2.99083000	0.48192700
C(PDBName=C16,ResName=UNK,ResNum=900)	2.13612800	3.54616600	0.00101200

H(PDBName=H1,ResName=UNK,ResNum=900) -1.25307500 0.59810300 -1.42229100
H(PDBName=H2,ResName=UNK,ResNum=900) -0.46145500 1.51659200 -2.72884000
H(PDBName=H3,ResName=UNK,ResNum=900) -0.61130000 -0.24931800 -2.85347000
H(PDBName=H4,ResName=UNK,ResNum=900) 3.36644300 -2.39821900 -0.53340200
H(PDBName=H5,ResName=UNK,ResNum=900) 4.56616200 -1.26171400 -1.19802900
H(PDBName=H6,ResName=UNK,ResNum=900) 4.14042300 -1.20727600 0.53446000
H(PDBName=H7,ResName=UNK,ResNum=900) -1.22560600 -3.37051300 0.15851200
H(PDBName=H8,ResName=UNK,ResNum=900) -1.31490900 -2.84128600 -1.59735100
H(PDBName=H9,ResName=UNK,ResNum=900) 2.49195000 -0.03636500 3.56637000
H(PDBName=H10,ResName=UNK,ResNum=900) -0.92409000 0.13030800 0.96609800
H(PDBName=H11,ResName=UNK,ResNum=900) 0.59966500 1.27148700 2.57313300
H(PDBName=H12,ResName=UNK,ResNum=900) 2.78346800 -2.42618200 2.87951000
H(PDBName=H13,ResName=UNK,ResNum=900) 1.22463600 -3.37609100 1.18258300
H(PDBName=H14,ResName=UNK,ResNum=900) 0.28963600 3.21302900 -1.06873800
H(PDBName=H15,ResName=UNK,ResNum=900) 1.92690500 4.59240200 0.18701800
H(PDBName=H16,ResName=UNK,ResNum=900) 4.02173600 3.61845600 1.03083100
H(PDBName=H17,ResName=UNK,ResNum=900) 4.56446600 1.22765800 0.65407100
H(PDBName=H18,ResName=UNK,ResNum=900) -1.83549100 -1.67505200 -0.28336800
I -3.89941000 0.45702500 0.17922700

---

23. benzimidazolium-CH_3SO_3-in

\# opt=calcfc freq=noraman wb97xd/6-311g(d,p) scrf=(iefpcm,solvent=dmso
) geom=connectivity temperature=363

Zero-point correction= 0.270742 (Hartree/Particle)
Thermal correction to Energy= 0.297992
Thermal correction to Enthalpy= 0.299142
Thermal correction to Gibbs Free Energy= 0.206026
Sum of electronic and zero-point Energies= -1560.042475
Sum of electronic and thermal Energies= -1560.015225
Sum of electronic and thermal Enthalpies= -1560.014076
Sum of electronic and thermal Free Energies= -1560.107191

Title Card Required

0 1

C(PDBName=C1,ResName=UNK,ResNum=900)	1.45610000	-0.65923000	0.76346600
S(PDBName=S2,ResName=UNK,ResNum=900)	-0.85467700	2.08174500	-0.10723600
O(PDBName=O3,ResName=UNK,ResNum=900)	0.37458600	2.88601800	-0.24730800
O(PDBName=O4,ResName=UNK,ResNum=900)	-1.01732300	1.50081700	1.24188300
S(PDBName=S3,ResName=UNK,ResNum=900)	-1.90835200	-2.46866500	-0.05711200
C(PDBName=C4,ResName=UNK,ResNum=900)	-0.35116400	-1.67281600	0.00112800
C(PDBName=C5,ResName=UNK,ResNum=900)	-3.00024400	-1.02946800	0.22617200
N(PDBName=N2,ResName=UNK,ResNum=900)	0.43697400	-1.44057500	-1.05647100
N(PDBName=N3,ResName=UNK,ResNum=900)	0.25664900	-1.24742900	1.11625600
C(PDBName=C6,ResName=UNK,ResNum=900)	-0.28437400	-1.26322400	2.46571600
C(PDBName=C7,ResName=UNK,ResNum=900)	0.09397700	-1.60527400	-2.45963700
C(PDBName=C8,ResName=UNK,ResNum=900)	1.57439600	-0.78992500	-0.61762200
C(PDBName=C9,ResName=UNK,ResNum=900)	2.67015900	-0.28366100	-1.31124000
C(PDBName=C10,ResName=UNK,ResNum=900)	2.42746200	-0.01673200	1.52547400
C(PDBName=C11,ResName=UNK,ResNum=900)	3.51572000	0.48759500	0.83877900
C(PDBName=C12,ResName=UNK,ResNum=900)	3.63533000	0.35637600	-0.55663600
H(PDBName=H1,ResName=UNK,ResNum=900)	-2.79780800	-0.57936200	1.19538600
H(PDBName=H2,ResName=UNK,ResNum=900)	-2.84134000	-0.29970300	-0.56334600
H(PDBName=H3,ResName=UNK,ResNum=900)	-4.00993500	-1.43755700	0.19789500
H(PDBName=H4,ResName=UNK,ResNum=900)	-1.07278300	-2.01035600	2.52097500
H(PDBName=H5,ResName=UNK,ResNum=900)	0.51114100	-1.52940500	3.15982500
H(PDBName=H6,ResName=UNK,ResNum=900)	-0.67714000	-0.27005800	2.68252200
H(PDBName=H7,ResName=UNK,ResNum=900)	0.91387600	-2.10741200	-2.97127800
H(PDBName=H8,ResName=UNK,ResNum=900)	-0.80744700	-2.20623300	-2.53620600
H(PDBName=H9,ResName=UNK,ResNum=900)	-0.08236600	-0.61693300	-2.88350800
H(PDBName=H10,ResName=UNK,ResNum=900)	4.29527300	1.00220400	1.38688200
H(PDBName=H11,ResName=UNK,ResNum=900)	2.75766100	-0.37820100	-2.38560000
H(PDBName=H12,ResName=UNK,ResNum=900)	4.50396300	0.77316200	-1.05124600
H(PDBName=H13,ResName=UNK,ResNum=900)	2.32686100	0.09771400	2.59662400
O	-1.01775700	1.08573600	-1.18935900
C	-2.22238200	3.22025200	-0.30219700

H	-2.15899700	3.68031800	-1.28740700
H	-3.15366600	2.66294600	-0.20677100
H	-2.16012900	3.98169600	0.47417200

24. benzimidazolium-CH_3SO_3

----

opt=calcfc freq=noraman wb97xd/6-311g(d,p) scrf=(iefpcm,solvent=dmso
) geom=connectivity temperature=363

Zero-point correction= 0.270389 (Hartree/Particle)
Thermal correction to Energy= 0.297800
Thermal correction to Enthalpy= 0.298949
Thermal correction to Gibbs Free Energy= 0.205084
Sum of electronic and zero-point Energies= -1560.042580
Sum of electronic and thermal Energies= -1560.015169
Sum of electronic and thermal Enthalpies= -1560.014020
Sum of electronic and thermal Free Energies= -1560.107885

Title Card Required

0 1

C(PDBName=C1,ResName=UNK,ResNum=900)	0.04389800	1.29452600	0.84565100
S(PDBName=S1,ResName=UNK,ResNum=900)	1.89638000	-1.72776400	-0.24493900
O(PDBName=O1,ResName=UNK,ResNum=900)	0.51288800	-1.67342100	-0.76875900
O(PDBName=O2,ResName=UNK,ResNum=900)	2.60080400	-2.96839100	-0.61950800
C(PDBName=C3,ResName=UNK,ResNum=900)	1.04181200	1.94098300	1.56927300
C(PDBName=C4,ResName=UNK,ResNum=900)	0.41417600	2.68019700	-1.12115900
C(PDBName=C5,ResName=UNK,ResNum=900)	1.40498000	3.32415600	-0.40306500
C(PDBName=C6,ResName=UNK,ResNum=900)	1.71495600	2.95871000	0.91973600
C(PDBName=C7,ResName=UNK,ResNum=900)	-1.78978100	0.76519000	-2.24327100
C(PDBName=C8,ResName=UNK,ResNum=900)	-0.74553100	-0.46086200	2.44895600
C(PDBName=C9,ResName=UNK,ResNum=900)	-1.53896900	-0.05225700	0.10820900
C(PDBName=C10,ResName=UNK,ResNum=900)	-0.25642500	1.65027800	-0.46678700
N(PDBName=N1,ResName=UNK,ResNum=900)	-0.79704400	0.24941700	1.17876100

N(PDBName=N2,ResName=UNK,ResNum=900)	-1.25214400	0.79190000	-0.89297800
S(PDBName=S2,ResName=UNK,ResNum=900)	-2.67771700	-1.37632700	0.01270000
C(PDBName=C11,ResName=UNK,ResNum=900)	-4.23371500	-0.43479200	0.18256000
O(PDBName=O3,ResName=UNK,ResNum=900)	1.97525600	-1.43329800	1.20177800
H(PDBName=H1,ResName=UNK,ResNum=900)	2.50525000	3.48596700	1.43964500
H(PDBName=H2,ResName=UNK,ResNum=900)	0.18046300	2.95456600	-2.14149600
H(PDBName=H3,ResName=UNK,ResNum=900)	1.95989400	4.12768400	-0.87122600
H(PDBName=H4,ResName=UNK,ResNum=900)	1.28694900	1.65020400	2.58183300
H(PDBName=H5,ResName=UNK,ResNum=900)	-2.41064000	1.64488900	-2.41214100
H(PDBName=H6,ResName=UNK,ResNum=900)	-0.96137400	0.75217200	-2.95066300
H(PDBName=H7,ResName=UNK,ResNum=900)	-2.37984800	-0.13893100	-2.36848800
H(PDBName=H8,ResName=UNK,ResNum=900)	-0.82839400	0.26371000	3.25826700
H(PDBName=H9,ResName=UNK,ResNum=900)	-1.57690400	-1.15865000	2.49497300
H(PDBName=H10,ResName=UNK,ResNum=900)	0.20494600	-0.99388400	2.49061300
H(PDBName=H11,ResName=UNK,ResNum=900)	-5.02595400	-1.17957600	0.11839100
H(PDBName=H12,ResName=UNK,ResNum=900)	-4.27348000	0.06153100	1.14972800
H(PDBName=H13,ResName=UNK,ResNum=900)	-4.34465900	0.28225100	-0.62910200
C	2.77210300	-0.39493100	-1.05750900
H	3.80916500	-0.40493700	-0.72453700
H	2.72057800	-0.54753300	-2.13477900
H	2.30309900	0.55047400	-0.78933700

25. TS-benzimidazolium-CH_3SO_3-py

----

opt=(calcfc,ts,noeigen) freq=noraman wb97xd/6-311g(d,p) scrf=(iefpcm
,solvent=dmso) geom=connectivity temperature=363

Zero-point correction= 0.360807 (Hartree/Particle)

Thermal correction to Energy= 0.396066

Thermal correction to Enthalpy= 0.397216

Thermal correction to Gibbs Free Energy= 0.281587

Sum of electronic and zero-point Energies= -1808.175659

Sum of electronic and thermal Energies= -1808.140400

Sum of electronic and thermal Enthalpies= -1808.139250

Sum of electronic and thermal Free Energies= -1808.254878

Title Card Required

0 1

C	0.99282500	1.28687300	0.74800900
C	1.21290500	1.15148100	-0.62015700
N	0.64880000	-0.05282100	-0.99802300
N	0.31030200	0.15487500	1.15369700
C	0.74959400	-0.60324300	-2.33873200
H	0.35371500	0.11806600	-3.05442700
H	1.80213200	-0.81117600	-2.53615100
H	0.16644300	-1.51942800	-2.38163000
C	-0.01101400	-0.14289900	2.53647700
H	-0.67694900	0.62316000	2.93482300
H	-0.49916600	-1.11314600	2.57954700
H	0.91049700	-0.17464000	3.11920400
C	0.12820100	-0.65697000	0.08884600
S	-0.68822000	-2.16878400	0.10857000
S	4.11909600	-1.04712700	-0.07733100
O	3.82069600	-0.78518100	-1.50184900
O	5.21822500	-2.01657300	0.10568100
C	-2.81792300	-1.23816300	-0.00226100
H	-2.55696600	-0.71163800	-0.90438100
H	-3.24903500	-2.22340400	-0.06878700
H	-2.69885500	-0.76938500	0.95881100
C	-5.73935000	-1.04730500	0.42732900
C	-7.01345100	-0.50269700	0.41342100
C	-7.19944700	0.73855700	-0.18124200
C	-6.10780100	1.39071500	-0.73982500
C	-4.86765000	0.77553700	-0.68220300
N	-4.69622100	-0.41656600	-0.11150200
H	-8.18245400	1.19251600	-0.20917600
H	-5.54193900	-2.01249500	0.88112800

H	-7.83742700	-1.04300300	0.86041200
H	-6.20984000	2.35860100	-1.21246900
H	-3.98385600	1.24426800	-1.10192100
C	1.88452200	2.12332500	-1.35087100
C	1.43090900	2.40141900	1.45323800
C	2.09763100	3.37588700	0.72593100
C	2.32079600	3.23855400	-0.65258100
H	2.06992300	2.00629800	-2.41027700
H	2.85190100	4.02045300	-1.18162500
H	2.45959500	4.26119400	1.23428700
H	1.26324000	2.50452600	2.51768900
O	2.91915700	-1.38454600	0.71547400
C	4.71613800	0.50433100	0.58535600
H	4.94423100	0.36716500	1.64174700
H	5.61367200	0.79691100	0.04166100
H	3.94100900	1.25930400	0.46363900

26. TS-benzimidazolium-CH_3SO_3—py(2Step)

----

opt=(calcfc,ts,noeigen) freq=noraman wb97xd/6-311g(d,p) scrf=(iefpcm
,solvent=dmso) geom=connectivity temperature=363

Zero-point correction= 0.361669 (Hartree/Particle)
Thermal correction to Energy= 0.396238
Thermal correction to Enthalpy= 0.397388
Thermal correction to Gibbs Free Energy= 0.287593
Sum of electronic and zero-point Energies= -1808.182362
Sum of electronic and thermal Energies= -1808.147793
Sum of electronic and thermal Enthalpies= -1808.146644
Sum of electronic and thermal Free Energies= -1808.256438

Title Card Required

0 1

C(PDBName=C,ResName=UNK,ResNum=0)	-2.55667200	0.94097400	-0.99483200
C(PDBName=C,ResName=UNK,ResNum=0)	-3.04484300	0.13504500	0.02983200
N(PDBName=N,ResName=UNK,ResNum=0)	-2.33613100	0.46542700	1.17117300
N(PDBName=N,ResName=UNK,ResNum=0)	-1.57898700	1.74484000	-0.43431400
C(PDBName=C,ResName=UNK,ResNum=0)	-2.49736300	-0.21318100	2.44471600
C(PDBName=C,ResName=UNK,ResNum=0)	-0.78328200	2.70859200	-1.16961800
C(PDBName=C,ResName=UNK,ResNum=0)	-1.44330200	1.43209200	0.87320500
S(PDBName=S,ResName=UNK,ResNum=0)	-0.34112900	2.15769600	1.97037100
S(PDBName=S,ResName=UNK,ResNum=0)	0.16384200	-2.04638300	0.35448400
O(PDBName=O,ResName=UNK,ResNum=0)	0.97093300	-1.82943100	1.57602000
O(PDBName=O,ResName=UNK,ResNum=0)	0.11332500	-0.84692200	-0.51081600
C(PDBName=C,ResName=UNK,ResNum=0)	1.62542100	1.51012800	0.93719700
C(PDBName=C,ResName=UNK,ResNum=0)	4.16944300	1.57870600	-0.70383200
C(PDBName=C,ResName=UNK,ResNum=0)	5.29943900	1.06597300	-1.32258900
C(PDBName=C,ResName=UNK,ResNum=0)	5.59974400	-0.27865000	-1.14729000
C(PDBName=C,ResName=UNK,ResNum=0)	4.76293900	-1.06074700	-0.36149100
C(PDBName=C,ResName=UNK,ResNum=0)	3.65194400	-0.47031600	0.22120800
N(PDBName=N,ResName=UNK,ResNum=0)	3.37165100	0.82259700	0.04824500
C(PDBName=C,ResName=UNK,ResNum=0)	-4.05276500	-0.79452000	-0.18782500
C(PDBName=C,ResName=UNK,ResNum=0)	-3.04863200	0.84992000	-2.29105500
C(PDBName=C,ResName=UNK,ResNum=0)	-4.05510800	-0.07808900	-2.51266900
C(PDBName=C,ResName=UNK,ResNum=0)	-4.54932300	-0.88642000	-1.47876400
O(PDBName=O,ResName=UNK,ResNum=0)	-1.16465400	-2.61902700	0.64074200
C(PDBName=C,ResName=UNK,ResNum=0)	1.04234500	-3.28094900	-0.59902800
H(PDBName=H,ResName=UNK,ResNum=0)	-1.74077000	0.15574800	3.13257800
H(PDBName=H,ResName=UNK,ResNum=0)	-3.49033400	-0.01236600	2.84902900
H(PDBName=H,ResName=UNK,ResNum=0)	-2.35449600	-1.28112900	2.27797700
H(PDBName=H,ResName=UNK,ResNum=0)	-0.39597200	3.44896100	-0.47272000
H(PDBName=H,ResName=UNK,ResNum=0)	0.04299100	2.20695400	-1.67595900
H(PDBName=H,ResName=UNK,ResNum=0)	-1.41754700	3.20577400	-1.90223400
H(PDBName=H,ResName=UNK,ResNum=0)	1.11221500	0.71220700	0.41696500
H(PDBName=H,ResName=UNK,ResNum=0)	1.77298000	2.47293800	0.47658900
H(PDBName=H,ResName=UNK,ResNum=0)	2.07127900	1.31845400	1.89892200
H(PDBName=H,ResName=UNK,ResNum=0)	6.47491600	-0.71103100	-1.61718000

H(PDBName=H,ResName=UNK,ResNum=0)	3.89188800	2.62204000	-0.81113300
H(PDBName=H,ResName=UNK,ResNum=0)	5.92466000	1.70945600	-1.92763500
H(PDBName=H,ResName=UNK,ResNum=0)	4.96148900	-2.11253700	-0.20052100
H(PDBName=H,ResName=UNK,ResNum=0)	2.95232500	-1.03071600	0.83692000
H(PDBName=H,ResName=UNK,ResNum=0)	-4.42219500	-1.42591000	0.60962000
H(PDBName=H,ResName=UNK,ResNum=0)	-5.33259900	-1.60257100	-1.69565700
H(PDBName=H,ResName=UNK,ResNum=0)	-4.46815500	-0.18299100	-3.50862100
H(PDBName=H,ResName=UNK,ResNum=0)	-2.66383700	1.46753300	-3.09239600
H(PDBName=H,ResName=UNK,ResNum=0)	0.49276200	-3.46737900	-1.52069600
H(PDBName=H,ResName=UNK,ResNum=0)	2.03687400	-2.89841100	-0.82729700
H(PDBName=H,ResName=UNK,ResNum=0)	1.11563400	-4.19438400	-0.01050700

27. TS-benzimidazolium-py-CH_3SO_3-conformr2(2Step)

 # opt=(calcfc,ts,noeigen) freq=noraman wb97xd/6-311g(d,p) scrf=(iefpcm
 ,solvent=dmso) geom=connectivity temperature=363

Zero-point correction= 0.360761 (Hartree/Particle)
 Thermal correction to Energy= 0.395588
 Thermal correction to Enthalpy= 0.396737
 Thermal correction to Gibbs Free Energy= 0.285588
 Sum of electronic and zero-point Energies= -1808.180733
 Sum of electronic and thermal Energies= -1808.145906
 Sum of electronic and thermal Enthalpies= -1808.144757
 Sum of electronic and thermal Free Energies= -1808.255906

Title Card Required

0 1

C(PDBName=C,ResName=UNK,ResNum=0)	-2.80672800	0.53692700	0.29598700
C(PDBName=C,ResName=UNK,ResNum=0)	-1.96343700	0.23084300	1.36149500
N(PDBName=N,ResName=UNK,ResNum=0)	-0.86136800	1.06430200	1.24780000
N(PDBName=N,ResName=UNK,ResNum=0)	-2.17881300	1.52927400	-0.43831100
C(PDBName=C,ResName=UNK,ResNum=0)	0.26220800	1.08119700	2.16996900

C(PDBName=C,ResName=UNK,ResNum=0)	-2.72130800	2.11399900	-1.65005600
C(PDBName=C,ResName=UNK,ResNum=0)	-0.99702800	1.83845600	0.14636900
S(PDBName=S,ResName=UNK,ResNum=0)	0.11277700	3.01386200	-0.41014500
S(PDBName=S,ResName=UNK,ResNum=0)	3.91160400	-0.62835500	0.10507800
O(PDBName=O,ResName=UNK,ResNum=0)	2.83529000	-0.73794800	1.09972800
O(PDBName=O,ResName=UNK,ResNum=0)	3.68244300	0.53328000	-0.83959200
C(PDBName=C,ResName=UNK,ResNum=0)	2.08489600	1.63322400	-0.59525600
C(PDBName=C,ResName=UNK,ResNum=0)	-0.83228300	-0.78318700	-2.28739300
C(PDBName=C,ResName=UNK,ResNum=0)	-1.90591500	-1.66055000	-2.33377300
C(PDBName=C,ResName=UNK,ResNum=0)	-2.00896400	-2.63355500	-1.34706100
C(PDBName=C,ResName=UNK,ResNum=0)	-1.03411800	-2.68851100	-0.36209300
C(PDBName=C,ResName=UNK,ResNum=0)	0.00333400	-1.76415900	-0.39992100
N(PDBName=N,ResName=UNK,ResNum=0)	0.10894300	-0.82433500	-1.34142900
C(PDBName=C,ResName=UNK,ResNum=0)	-2.28706900	-0.75544300	2.28472700
C(PDBName=C,ResName=UNK,ResNum=0)	-4.01702800	-0.11713600	0.10939500
C(PDBName=C,ResName=UNK,ResNum=0)	-4.34344900	-1.10044100	1.03014900
C(PDBName=C,ResName=UNK,ResNum=0)	-3.49258300	-1.41446400	2.09855700
O(PDBName=O,ResName=UNK,ResNum=0)	4.20025700	-1.85849200	-0.63393400
C(PDBName=C,ResName=UNK,ResNum=0)	5.38564600	-0.17645500	1.00055800
H(PDBName=H,ResName=UNK,ResNum=0)	0.67085300	2.09014200	2.20355600
H(PDBName=H,ResName=UNK,ResNum=0)	-0.10154500	0.81628000	3.16150400
H(PDBName=H,ResName=UNK,ResNum=0)	1.03454700	0.37787800	1.85082400
H(PDBName=H,ResName=UNK,ResNum=0)	-1.91959200	2.62121200	-2.18161300
H(PDBName=H,ResName=UNK,ResNum=0)	-3.12566900	1.31771500	-2.27537200
H(PDBName=H,ResName=UNK,ResNum=0)	-3.50937400	2.82781400	-1.40606900
H(PDBName=H,ResName=UNK,ResNum=0)	2.34507400	1.85902800	0.42314000
H(PDBName=H,ResName=UNK,ResNum=0)	2.43680000	2.28554100	-1.37808800
H(PDBName=H,ResName=UNK,ResNum=0)	1.44154900	0.79693200	-0.83823400
H(PDBName=H,ResName=UNK,ResNum=0)	-2.83704700	-3.33249300	-1.34441900
H(PDBName=H,ResName=UNK,ResNum=0)	-0.72256500	-0.00646300	-3.03895300
H(PDBName=H,ResName=UNK,ResNum=0)	-2.64476400	-1.57733500	-3.12121200
H(PDBName=H,ResName=UNK,ResNum=0)	-1.07645000	-3.42486500	0.43041800
H(PDBName=H,ResName=UNK,ResNum=0)	0.78664700	-1.76150000	0.35308100
H(PDBName=H,ResName=UNK,ResNum=0)	-1.62623300	-1.00872200	3.10357300

H(PDBName=H,ResName=UNK,ResNum=0)	-3.78203400	-2.19166300	2.79529900
H(PDBName=H,ResName=UNK,ResNum=0)	-5.27733500	-1.63810900	0.92056300
H(PDBName=H,ResName=UNK,ResNum=0)	-4.67227800	0.12482400	-0.71752200
H(PDBName=H,ResName=UNK,ResNum=0)	6.20139600	-0.05332700	0.29016900
H(PDBName=H,ResName=UNK,ResNum=0)	5.19325300	0.75744300	1.52611600
H(PDBName=H,ResName=UNK,ResNum=0)	5.61235400	-0.97231600	1.70835300

28. **TS-benzimidazolium-benzimidazolium-CH_3SO_3-py-conformr3-** --

opt=(calcfc,ts,noeigen) freq=noraman wb97xd/6-311g(d,p) scrf=(iefpcm ,solvent=dmso) geom=connectivity temperature=363

Zero-point correction= 0.269894 (Hartree/Particle)
Thermal correction to Energy= 0.296401
Thermal correction to Enthalpy= 0.297551
Thermal correction to Gibbs Free Energy= 0.206099
Sum of electronic and zero-point Energies= -1559.993030
Sum of electronic and thermal Energies= -1559.966522
Sum of electronic and thermal Enthalpies= -1559.965372
Sum of electronic and thermal Free Energies= -1560.056824

Title Card Required

0 1

C(PDBName=C,ResName=UNK,ResNum=0)	-2.91118700	-0.08181100	-0.33897200
C(PDBName=C,ResName=UNK,ResNum=0)	-2.08622700	-0.82148600	0.50535900
N(PDBName=N,ResName=UNK,ResNum=0)	-1.10115700	0.04373500	0.95746900
N(PDBName=N,ResName=UNK,ResNum=0)	-2.38742200	1.20035500	-0.38705200
C(PDBName=C,ResName=UNK,ResNum=0)	-0.05769300	-0.32424000	1.90223700
C(PDBName=C,ResName=UNK,ResNum=0)	-2.94780300	2.28207200	-1.17754700
C(PDBName=C,ResName=UNK,ResNum=0)	-1.29152400	1.26549100	0.40441000
S(PDBName=S,ResName=UNK,ResNum=0)	-0.31009400	2.64612800	0.64548400
S(PDBName=S,ResName=UNK,ResNum=0)	3.55999100	-0.66522600	-0.50205100
O(PDBName=O,ResName=UNK,ResNum=0)	2.25029900	-1.32685500	-0.42532500
O(PDBName=O,ResName=UNK,ResNum=0)	3.40597900	0.82927500	-0.71557200

C(PDBName=C,ResName=UNK,ResNum=0)	1.75024300	1.64302000	-0.08235500
C(PDBName=C,ResName=UNK,ResNum=0)	-2.32293300	-2.16598600	0.76220800
C(PDBName=C,ResName=UNK,ResNum=0)	-4.01881600	-0.64737300	-0.95792300
C(PDBName=C,ResName=UNK,ResNum=0)	-4.26012200	-1.98857000	-0.70073600
C(PDBName=C,ResName=UNK,ResNum=0)	-3.42651100	-2.73486500	0.14377800
O(PDBName=O,ResName=UNK,ResNum=0)	4.49119500	-1.22401000	-1.47967200
C(PDBName=C,ResName=UNK,ResNum=0)	4.30893700	-0.82119400	1.10732200
H(PDBName=H,ResName=UNK,ResNum=0)	0.31825800	0.58162300	2.37342500
H(PDBName=H,ResName=UNK,ResNum=0)	-0.49018500	-0.96984200	2.66577400
H(PDBName=H,ResName=UNK,ResNum=0)	0.75231400	-0.84208800	1.38468800
H(PDBName=H,ResName=UNK,ResNum=0)	-2.21952000	3.08751300	-1.22823200
H(PDBName=H,ResName=UNK,ResNum=0)	-3.15687000	1.91515600	-2.18225400
H(PDBName=H,ResName=UNK,ResNum=0)	-3.86728000	2.64596100	-0.71732200
H(PDBName=H,ResName=UNK,ResNum=0)	2.10815600	1.66325500	0.93167500
H(PDBName=H,ResName=UNK,ResNum=0)	1.93116200	2.48617000	-0.72653500
H(PDBName=H,ResName=UNK,ResNum=0)	1.18849600	0.80003000	-0.44652200
H(PDBName=H,ResName=UNK,ResNum=0)	-1.67684200	-2.74509800	1.40931000
H(PDBName=H,ResName=UNK,ResNum=0)	-3.64686700	-3.78113700	0.31645900
H(PDBName=H,ResName=UNK,ResNum=0)	-5.11284500	-2.47016000	-1.16339100
H(PDBName=H,ResName=UNK,ResNum=0)	-4.66309100	-0.06938300	-1.60796200
H(PDBName=H,ResName=UNK,ResNum=0)	5.26493800	-0.30065400	1.10010300
H(PDBName=H,ResName=UNK,ResNum=0)	3.64088100	-0.38146600	1.84689200
H(PDBName=H,ResName=UNK,ResNum=0)	4.45302700	-1.88056200	1.31391300

29. TS-benzimidazolium-py-CH_3SO_3-v3

----

opt=(calcfc,ts,noeigen) freq=noraman wb97xd/6-311g(d,p) scrf=(iefpcm

,solvent=dmso) geom=connectivity temperature=363

Zero-point correction= 0.180775 (Hartree/Particle)

Thermal correction to Energy= 0.198400

Thermal correction to Enthalpy= 0.199550

Thermal correction to Gibbs Free Energy= 0.126069

Sum of electronic and zero-point Energies= -951.671836

Sum of electronic and thermal Energies= -951.654211

Sum of electronic and thermal Enthalpies= -951.653061

Sum of electronic and thermal Free Energies= -951.726542

Title Card Required

0 1

S(PDBName=S,ResName=UNK,ResNum=0)	2.87685500	0.02531700	0.14296400
O(PDBName=O,ResName=UNK,ResNum=0)	2.36639000	1.08355000	1.01792100
O(PDBName=O,ResName=UNK,ResNum=0)	1.84135500	-1.07686600	-0.04168300
C(PDBName=C,ResName=UNK,ResNum=0)	0.02682000	-0.63963100	-0.01421500
C(PDBName=C,ResName=UNK,ResNum=0)	-2.89527300	-1.20974600	-0.08059900
C(PDBName=C,ResName=UNK,ResNum=0)	-4.25790400	-0.95767500	-0.09605600
C(PDBName=C,ResName=UNK,ResNum=0)	-4.69096300	0.35929200	-0.01061400
C(PDBName=C,ResName=UNK,ResNum=0)	-3.74841300	1.37455400	0.08705900
C(PDBName=C,ResName=UNK,ResNum=0)	-2.40543700	1.03332300	0.09627700
N(PDBName=N,ResName=UNK,ResNum=0)	-1.99409900	-0.23210800	0.01463000
O(PDBName=O,ResName=UNK,ResNum=0)	4.15556000	-0.56464800	0.52739800
C(PDBName=C,ResName=UNK,ResNum=0)	3.07200900	0.73454700	-1.47952100
H(PDBName=H,ResName=UNK,ResNum=0)	-0.00222600	-0.46702400	-1.07620700
H(PDBName=H,ResName=UNK,ResNum=0)	-0.18932000	-1.62132000	0.37097600
H(PDBName=H,ResName=UNK,ResNum=0)	0.16471000	0.18358200	0.66796100
H(PDBName=H,ResName=UNK,ResNum=0)	-5.74920700	0.59047500	-0.02024600
H(PDBName=H,ResName=UNK,ResNum=0)	-2.50647500	-2.22032900	-0.14741400
H(PDBName=H,ResName=UNK,ResNum=0)	-4.95964000	-1.77771900	-0.17271600
H(PDBName=H,ResName=UNK,ResNum=0)	-4.04238500	2.41361600	0.15610000
H(PDBName=H,ResName=UNK,ResNum=0)	-1.63102000	1.78961700	0.16940400
H(PDBName=H,ResName=UNK,ResNum=0)	3.40243100	-0.04539600	-2.16320600
H(PDBName=H,ResName=UNK,ResNum=0)	2.11436900	1.14295700	-1.80016200
H(PDBName=H,ResName=UNK,ResNum=0)	3.81629000	1.52694800	-1.41740400

30. benzimidazolium-CF_3SO_3-in

----

opt=calcfc freq=noraman wb97xd/6-311g(d,p) scrf=(iefpcm,solvent=dmso

) geom=connectivity temperature=363

Zero-point correction= 0.247088 (Hartree/Particle)

Thermal correction to Energy= 0.277399

Thermal correction to Enthalpy= 0.278549

Thermal correction to Gibbs Free Energy= 0.175031

Sum of electronic and zero-point Energies= -1857.787996

Sum of electronic and thermal Energies= -1857.757684

Sum of electronic and thermal Enthalpies= -1857.756535

Sum of electronic and thermal Free Energies= -1857.860052

Title Card Required

0 1

C(PDBName=C1,ResName=UNK,ResNum=900)	-2.09042700	-0.59471500	0.71851300
S(PDBName=S2,ResName=UNK,ResNum=900)	1.48003500	-0.74057100	0.01429500
C(PDBName=C2,ResName=UNK,ResNum=900)	3.29667700	-0.37535800	-0.08214300
O(PDBName=O3,ResName=UNK,ResNum=900)	1.40457700	-2.19711400	-0.08667900
O(PDBName=O4,ResName=UNK,ResNum=900)	1.10876200	-0.19119500	1.32067300
F(PDBName=F1,ResName=UNK,ResNum=900)	3.51712700	0.93835900	0.00515600
F(PDBName=F2,ResName=UNK,ResNum=900)	3.95500500	-0.97278600	0.91250800
F(PDBName=F3,ResName=UNK,ResNum=900)	3.80772900	-0.80551700	-1.23670200
S(PDBName=S3,ResName=UNK,ResNum=900)	-1.16927700	3.12693600	-0.03961500
C(PDBName=C4,ResName=UNK,ResNum=900)	-1.62120500	1.43625900	-0.01288300
C(PDBName=C5,ResName=UNK,ResNum=900)	0.64044600	2.94580000	0.16084700
N(PDBName=N2,ResName=UNK,ResNum=900)	-1.90440900	0.69202400	-1.08994200
N(PDBName=N3,ResName=UNK,ResNum=900)	-1.75989600	0.69379200	1.09391400
C(PDBName=C6,ResName=UNK,ResNum=900)	-1.50102000	1.10462700	2.46483800
C(PDBName=C7,ResName=UNK,ResNum=900)	-1.75913000	1.07318400	-2.48577800
C(PDBName=C8,ResName=UNK,ResNum=900)	-2.18803200	-0.59392200	-0.67046500
C(PDBName=C9,ResName=UNK,ResNum=900)	-2.48567200	-1.75080600	-1.38579500
C(PDBName=C10,ResName=UNK,ResNum=900)	-2.28581000	-1.75186300	1.46773200
C(PDBName=C11,ResName=UNK,ResNum=900)	-2.58110200	-2.90101300	0.75959300
C(PDBName=C12,ResName=UNK,ResNum=900)	-2.67954600	-2.90049500	-0.64401900

H(PDBName=H1,ResName=UNK,ResNum=900) 0.86722200 2.39084500 1.06885700
H(PDBName=H2,ResName=UNK,ResNum=900) 1.05598100 2.43370700 -0.70242500
H(PDBName=H3,ResName=UNK,ResNum=900) 1.01925900 3.96461900 0.23223500
H(PDBName=H4,ResName=UNK,ResNum=900) -1.43125900 2.18890000 2.50085700
H(PDBName=H5,ResName=UNK,ResNum=900) -2.32589900 0.77389600 3.09427200
H(PDBName=H6,ResName=UNK,ResNum=900) -0.56256300 0.65408700 2.78550700
H(PDBName=H7,ResName=UNK,ResNum=900) -2.66858400 0.80933700 -3.02392000
H(PDBName=H8,ResName=UNK,ResNum=900) -1.59844000 2.14586000 -2.54542300
H(PDBName=H9,ResName=UNK,ResNum=900) -0.89913800 0.54602400 -2.89770000
H(PDBName=H10,ResName=UNK,ResNum=900) -2.73516500 -3.82894300 1.29622800
H(PDBName=H11,ResName=UNK,ResNum=900) -2.55160300 -1.75231000 -2.46593000
H(PDBName=H12,ResName=UNK,ResNum=900) -2.90651400 -3.82792800 -1.15503900
H(PDBName=H13,ResName=UNK,ResNum=900) -2.19600400 -1.75375100 2.54604900
O 0.93927300 -0.01388900 -1.13743600

---

31. benzimidazoilum-CF_3SO_3

\# opt=calcfc freq=noraman wb97xd/6-311g(d,p) scrf=(iefpcm,solvent=dmso
) geom=connectivity temperature=363

Zero-point correction= 0.247020 (Hartree/Particle)
Thermal correction to Energy= 0.277425
Thermal correction to Enthalpy= 0.278574
Thermal correction to Gibbs Free Energy= 0.175826
Sum of electronic and zero-point Energies= -1857.785960
Sum of electronic and thermal Energies= -1857.755555
Sum of electronic and thermal Enthalpies= -1857.754406
Sum of electronic and thermal Free Energies= -1857.857154

Title Card Required

0 1
C(PDBName=C1,ResName=UNK,ResNum=900) -1.07147900 1.36241200 0.85513600
S(PDBName=S1,ResName=UNK,ResNum=900) 1.92646300 -1.25666900 0.53025000

C(PDBName=C2,ResName=UNK,ResNum=900)	2.62105700	-0.06325400	-0.70832900
O(PDBName=O1,ResName=UNK,ResNum=900)	0.71429800	-1.74755200	-0.13214000
O(PDBName=O2,ResName=UNK,ResNum=900)	2.98558500	-2.25217600	0.68350300
F(PDBName=F1,ResName=UNK,ResNum=900)	1.75387600	0.91955000	-0.96028100
F(PDBName=F2,ResName=UNK,ResNum=900)	3.74897700	0.48660100	-0.25627700
F(PDBName=F3,ResName=UNK,ResNum=900)	2.89085700	-0.68025000	-1.86008200
C(PDBName=C3,ResName=UNK,ResNum=900)	-0.61746300	2.37915000	1.69000800
C(PDBName=C4,ResName=UNK,ResNum=900)	-0.89024100	2.75520400	-1.13200900
C(PDBName=C5,ResName=UNK,ResNum=900)	-0.44437600	3.76798100	-0.30394900
C(PDBName=C6,ResName=UNK,ResNum=900)	-0.30720900	3.58183400	1.08344500
C(PDBName=C7,ResName=UNK,ResNum=900)	-1.80941300	0.07785500	-2.46044300
C(PDBName=C8,ResName=UNK,ResNum=900)	-1.42596700	-0.54227200	2.44671400
C(PDBName=C9,ResName=UNK,ResNum=900)	-1.77952500	-0.53277800	-0.03154600
C(PDBName=C10,ResName=UNK,ResNum=900)	-1.19566800	1.54382200	-0.51890000
N(PDBName=N1,ResName=UNK,ResNum=900)	-1.46400100	0.06347700	1.12370600
N(PDBName=N2,ResName=UNK,ResNum=900)	-1.63114200	0.33858900	-1.04050500
S(PDBName=S2,ResName=UNK,ResNum=900)	-2.30528700	-2.19347600	-0.20164200
C(PDBName=C11,ResName=UNK,ResNum=900)	-4.10303400	-1.89839400	-0.33558300
O(PDBName=O3,ResName=UNK,ResNum=900)	1.68919300	-0.41828900	1.70671100
H(PDBName=H1,ResName=UNK,ResNum=900)	0.05273300	4.40291900	1.69103600
H(PDBName=H2,ResName=UNK,ResNum=900)	-0.99011900	2.89496600	-2.20029500
H(PDBName=H3,ResName=UNK,ResNum=900)	-0.19078800	4.72884600	-0.73431600
H(PDBName=H4,ResName=UNK,ResNum=900)	-0.50410100	2.23173800	2.75568300
H(PDBName=H5,ResName=UNK,ResNum=900)	-2.64350800	0.66660900	-2.84123400
H(PDBName=H6,ResName=UNK,ResNum=900)	-0.89159500	0.34611500	-2.98280300
H(PDBName=H7,ResName=UNK,ResNum=900)	-2.00621800	-0.98158800	-2.60167400
H(PDBName=H8,ResName=UNK,ResNum=900)	-1.99346800	0.08120600	3.13655900
H(PDBName=H9,ResName=UNK,ResNum=900)	-1.87371400	-1.53048600	2.39144100
H(PDBName=H10,ResName=UNK,ResNum=900)	-0.38334100	-0.61871500	2.75370200
H(PDBName=H11,ResName=UNK,ResNum=900)	-4.54850600	-2.88698000	-0.44212300
H(PDBName=H12,ResName=UNK,ResNum=900)	-4.48039100	-1.42347900	0.56770800
H(PDBName=H13,ResName=UNK,ResNum=900)	-4.33588900	-1.30163700	-1.21593300

32. TS-benzimidazolium-CF_3SO_3-back

----

opt=(calcfc,ts,noeigen) freq=noraman wb97xd/6-311g(d,p) scrf=(iefpcm
,solvent=dmso) geom=connectivity temperature=363

Zero-point correction= 0.336997 (Hartree/Particle)
Thermal correction to Energy= 0.375374
Thermal correction to Enthalpy= 0.376524
Thermal correction to Gibbs Free Energy= 0.251765
Sum of electronic and zero-point Energies= -2105.919214
Sum of electronic and thermal Energies= -2105.880837
Sum of electronic and thermal Enthalpies= -2105.879687
Sum of electronic and thermal Free Energies= -2106.004446

Title Card Required

0 1

C	0.65730000	1.36738600	1.01775600
C	0.47280400	1.32616700	-0.36154100
N	-0.20002000	0.14971000	-0.63892500
N	0.07774200	0.21939600	1.52980600
C	-0.51709700	-0.29946400	-1.98219600
H	-1.23541900	0.38158700	-2.44062800
H	0.40762400	-0.33434100	-2.55658000
H	-0.93865800	-1.30000300	-1.92298500
C	0.17317600	-0.19260700	2.91691000
H	-0.01099000	0.66847500	3.55815700
H	-0.57672100	-0.95558400	3.10963600
H	1.16861300	-0.59756500	3.10441000
C	-0.41409000	-0.51947800	0.51318800
S	-1.21925000	-2.02895800	0.66617200
S	3.24142500	-1.40171100	-0.47787500
O	2.54259100	-0.97426600	-1.69095900
O	4.04828500	-2.61333600	-0.63125700
C	-3.34892000	-1.18852700	0.25230200

H	-3.77716400	-2.13329100	0.54462200
H	-3.20477300	-0.41771100	0.98936200
H	-3.11994900	-1.00803500	-0.78340400
C	-5.33864200	0.76302400	-0.65707800
C	-6.57008800	1.34881200	-0.90020600
C	-7.71818000	0.65526700	-0.53853600
C	-7.59457700	-0.59571600	0.05149100
C	-6.32403900	-1.10908100	0.25919700
N	-5.22731700	-0.43828800	-0.09024300
H	-8.69659700	1.08575900	-0.71329000
H	-4.41095800	1.26172800	-0.91765700
H	-6.62300900	2.32538200	-1.36290000
H	-8.46367900	-1.16799500	0.34798300
H	-6.17457900	-2.08038700	0.71805800
C	0.92267800	2.34414700	-1.19201400
C	1.31016000	2.42817000	1.63347600
C	1.75957100	3.44706500	0.80823700
C	1.56934000	3.40619100	-0.58046600
H	0.78062100	2.30484700	-2.26430400
H	1.94096100	4.22138700	-1.18936100
H	2.27419300	4.29345600	1.24659400
H	1.46571200	2.45834700	2.70421300
O	2.45232100	-1.34407000	0.75300100
C	4.51015100	-0.07259700	-0.22562000
F	3.93550800	1.11956500	-0.07176900
F	5.24296500	-0.32160800	0.86318500
F	5.33755900	-0.00131900	-1.27195100

33. TS-benzimidazolium-CF_3SO_3-py-v2

----

opt=(calcfc,ts,noeigen) freq=noraman wb97xd/6-311g(d,p) scrf=(iefpcm

,solvent=dmso) geom=connectivity temperature=363

Zero-point correction= 0.337667 (Hartree/Particle)

Thermal correction to Energy= 0.375587

Thermal correction to Enthalpy= 0.376737

Thermal correction to Gibbs Free Energy= 0.256411

Sum of electronic and zero-point Energies= -2105.927556

Sum of electronic and thermal Energies= -2105.889636

Sum of electronic and thermal Enthalpies= -2105.888486

Sum of electronic and thermal Free Energies= -2106.008812

Title Card Required

0 1

C(PDBName=C,ResName=UNK,ResNum=0)	-2.68980500	-1.00030600	1.11512200
C(PDBName=C,ResName=UNK,ResNum=0)	-3.20468600	-0.38230800	-0.02082400
N(PDBName=N,ResName=UNK,ResNum=0)	-2.55003200	-0.93446000	-1.10829900
N(PDBName=N,ResName=UNK,ResNum=0)	-1.75542100	-1.92267600	0.67523600
C(PDBName=C,ResName=UNK,ResNum=0)	-2.76262000	-0.50969800	-2.48072000
C(PDBName=C,ResName=UNK,ResNum=0)	-0.95016300	-2.75649100	1.54709100
C(PDBName=C,ResName=UNK,ResNum=0)	-1.66711100	-1.85774600	-0.67137000
S(PDBName=S,ResName=UNK,ResNum=0)	-0.62747800	-2.79898100	-1.65974200
S(PDBName=S,ResName=UNK,ResNum=0)	0.05396400	1.53888000	-0.70982500
O(PDBName=O,ResName=UNK,ResNum=0)	0.94706400	1.17904300	-1.81491600
O(PDBName=O,ResName=UNK,ResNum=0)	-0.00549100	0.54697400	0.36805500
C(PDBName=C,ResName=UNK,ResNum=0)	1.40243400	-2.13362400	-0.77898500
C(PDBName=C,ResName=UNK,ResNum=0)	4.01041500	-2.22489000	0.73775900
C(PDBName=C,ResName=UNK,ResNum=0)	5.19317700	-1.74069100	1.27398800
C(PDBName=C,ResName=UNK,ResNum=0)	5.55227900	-0.42487900	1.00948400
C(PDBName=C,ResName=UNK,ResNum=0)	4.72037600	0.35665000	0.21904200
C(PDBName=C,ResName=UNK,ResNum=0)	3.55593500	-0.20567000	-0.28092800
N(PDBName=N,ResName=UNK,ResNum=0)	3.21754500	-1.46900200	-0.02083800
C(PDBName=C,ResName=UNK,ResNum=0)	-4.18277400	0.59957800	0.06027300
C(PDBName=C,ResName=UNK,ResNum=0)	-3.11929100	-0.65580000	2.39061200
C(PDBName=C,ResName=UNK,ResNum=0)	-4.09442200	0.32631600	2.47640300
C(PDBName=C,ResName=UNK,ResNum=0)	-4.61829800	0.94257300	1.33082100
O(PDBName=O,ResName=UNK,ResNum=0)	-1.23333200	2.09676200	-1.11860700

C(PDBName=C,ResName=UNK,ResNum=0)	0.91183200	2.97352600	0.09943600	
H(PDBName=H,ResName=UNK,ResNum=0)	-2.06165400	-1.03981300	-3.12022400	
H(PDBName=H,ResName=UNK,ResNum=0)	-3.78419900	-0.74203400	-2.78402000	
H(PDBName=H,ResName=UNK,ResNum=0)	-2.57958600	0.56328300	-2.54155700	
H(PDBName=H,ResName=UNK,ResNum=0)	-0.60483500	-3.62267300	0.98647500	
H(PDBName=H,ResName=UNK,ResNum=0)	-0.09592600	-2.19184300	1.92409700	
H(PDBName=H,ResName=UNK,ResNum=0)	-1.56499600	-3.09377400	2.38034800	
H(PDBName=H,ResName=UNK,ResNum=0)	0.95002700	-1.27146500	-0.31226000	
H(PDBName=H,ResName=UNK,ResNum=0)	1.52870000	-3.05648700	-0.23840800	
H(PDBName=H,ResName=UNK,ResNum=0)	1.81261100	-2.04206900	-1.77056500	
H(PDBName=H,ResName=UNK,ResNum=0)	6.46796500	-0.01387500	1.41729100	
H(PDBName=H,ResName=UNK,ResNum=0)	3.68401100	-3.24381000	0.91750500	
H(PDBName=H,ResName=UNK,ResNum=0)	5.81308100	-2.38247200	1.88622300	
H(PDBName=H,ResName=UNK,ResNum=0)	4.95971300	1.38774500	-0.00569300	
H(PDBName=H,ResName=UNK,ResNum=0)	2.86315000	0.35709800	-0.89932900	
H(PDBName=H,ResName=UNK,ResNum=0)	-4.57706900	1.08109800	-0.82503400	
H(PDBName=H,ResName=UNK,ResNum=0)	-5.37739200	1.70733100	1.44165100	
H(PDBName=H,ResName=UNK,ResNum=0)	-4.45832900	0.62644500	3.45159300	
H(PDBName=H,ResName=UNK,ResNum=0)	-2.71007400	-1.12363100	3.27673700	
F	1.07035700	3.97788200	-0.76321200	
F	2.11798700	2.61510300	0.54593400	
F	0.20241100	3.42079900	1.13572900	2.30281600

TS-benzimidazolium-CF_3SO_3-py(2Step)--

opt=(calcfc,ts,noeigen) freq=noraman wb97xd/6-311g(d,p) scrf=(iefpcm
,solvent=dmso) geom=connectivity temperature=363

Zero-point correction= 0.336361 (Hartree/Particle)
Thermal correction to Energy= 0.374409
Thermal correction to Enthalpy= 0.375558
Thermal correction to Gibbs Free Energy= 0.256228
Sum of electronic and zero-point Energies= -2105.880065
Sum of electronic and thermal Energies= -2105.842017
Sum of electronic and thermal Enthalpies= -2105.840867

Sum of electronic and thermal Free Energies= -2105.960197

Title Card Required

0 1

C(PDBName=C1,ResName=UNK,ResNum=900)	2.69422900	1.26248100	-0.76460700
C(PDBName=C2,ResName=UNK,ResNum=900)	1.32619400	1.37989200	-1.00349400
N(PDBName=N1,ResName=UNK,ResNum=900)	0.89731300	0.14553600	-1.46292600
N(PDBName=N2,ResName=UNK,ResNum=900)	3.04013900	-0.04687200	-1.05405100
C(PDBName=C3,ResName=UNK,ResNum=900)	-0.46746600	-0.18366100	-1.82889800
C(PDBName=C4,ResName=UNK,ResNum=900)	4.38554200	-0.58152000	-0.95314600
C(PDBName=C5,ResName=UNK,ResNum=900)	1.93561300	-0.72081900	-1.45809400
S(PDBName=S1,ResName=UNK,ResNum=900)	1.83243700	-2.38683200	-1.79144900
S(PDBName=S2,ResName=UNK,ResNum=900)	-3.13695500	0.10615100	0.91841900
C(PDBName=C6,ResName=UNK,ResNum=900)	-4.26765400	0.12711900	-0.55560800
O(PDBName=O1,ResName=UNK,ResNum=900)	-2.50902300	-1.21969700	0.83060200
O(PDBName=O2,ResName=UNK,ResNum=900)	-2.22446400	1.22247500	0.66686700
F(PDBName=F1,ResName=UNK,ResNum=900)	-4.90017200	1.29733400	-0.64234200
F(PDBName=F2,ResName=UNK,ResNum=900)	-3.58582900	-0.05986300	-1.68771000
F(PDBName=F3,ResName=UNK,ResNum=900)	-5.18220800	-0.83786600	-0.46036300
C(PDBName=C7,ResName=UNK,ResNum=900)	-0.02556600	-3.14312800	0.03192700
C(PDBName=C8,ResName=UNK,ResNum=900)	0.72564500	-0.49244700	1.63438400
C(PDBName=C9,ResName=UNK,ResNum=900)	1.44364900	0.42863000	2.38396800
C(PDBName=C10,ResName=UNK,ResNum=900)	2.74585100	0.11860100	2.74881000
C(PDBName=C11,ResName=UNK,ResNum=900)	3.28377100	-1.10115600	2.35505000
C(PDBName=C12,ResName=UNK,ResNum=900)	2.50367500	-1.95510900	1.59157300
N(PDBName=N3,ResName=UNK,ResNum=900)	1.25582000	-1.64931400	1.24164000
C(PDBName=C13,ResName=UNK,ResNum=900)	0.63518700	2.55415400	-0.73247500
C(PDBName=C14,ResName=UNK,ResNum=900)	3.44519300	2.32600700	-0.27974500
C(PDBName=C15,ResName=UNK,ResNum=900)	2.76182800	3.50438700	-0.02277200
C(PDBName=C16,ResName=UNK,ResNum=900)	1.38044300	3.61434300	-0.23996300
O(PDBName=O3,ResName=UNK,ResNum=900)	-4.04189400	0.27956000	2.05117000
H(PDBName=H1,ResName=UNK,ResNum=900)	-1.01246800	-0.57195000	-0.96924000
H(PDBName=H2,ResName=UNK,ResNum=900)	-0.95907000	0.71941500	-2.18330900

H(PDBName=H3,ResName=UNK,ResNum=900)	-0.45216700	-0.92730600	-2.62306400
H(PDBName=H4,ResName=UNK,ResNum=900)	4.32992900	-1.66769100	-0.96235300
H(PDBName=H5,ResName=UNK,ResNum=900)	4.98978900	-0.23808400	-1.79397000
H(PDBName=H6,ResName=UNK,ResNum=900)	4.83189700	-0.24782700	-0.01668100
H(PDBName=H7,ResName=UNK,ResNum=900)	0.44546800	-3.81326800	0.73483600
H(PDBName=H8,ResName=UNK,ResNum=900)	-0.17339300	-3.51178600	-0.96807800
H(PDBName=H9,ResName=UNK,ResNum=900)	3.33586300	0.81602200	3.33091700
H(PDBName=H10,ResName=UNK,ResNum=900)	-0.29745300	-0.29627500	1.33285600
H(PDBName=H11,ResName=UNK,ResNum=900)	0.98469100	1.36902700	2.66007300
H(PDBName=H12,ResName=UNK,ResNum=900)	4.29432500	-1.38417600	2.62015900
H(PDBName=H13,ResName=UNK,ResNum=900)	2.88943200	-2.90276500	1.23007200
H(PDBName=H14,ResName=UNK,ResNum=900)	-0.43651300	2.62342500	-0.86468500
H(PDBName=H15,ResName=UNK,ResNum=900)	0.88235300	4.54851200	-0.01099400
H(PDBName=H16,ResName=UNK,ResNum=900)	3.30551200	4.35780100	0.36359600
H(PDBName=H17,ResName=UNK,ResNum=900)	4.50878200	2.23561400	-0.10040900
H(PDBName=H18,ResName=UNK,ResNum=900)	-0.76746500	-2.44502300	0.39926900

TS-benzimidazolium-CF_3SO_3-py(2Step) -V2--

\# opt=(calcfc,ts,noeigen) freq=noraman wb97xd/6-311g(d,p) scrf=(iefpcm
,solvent=dmso) geom=connectivity temperature=363

Zero-point correction= 0.337586 (Hartree/Particle)
Thermal correction to Energy= 0.375278
Thermal correction to Enthalpy= 0.376427
Thermal correction to Gibbs Free Energy= 0.257580
Sum of electronic and zero-point Energies= -2105.918695
Sum of electronic and thermal Energies= -2105.881003
Sum of electronic and thermal Enthalpies= -2105.879854
Sum of electronic and thermal Free Energies= -2105.998701

Title Card Required

0 1

C(PDBName=C,ResName=UNK,ResNum=0)	-3.32276400	0.60147200	0.49645600

C(PDBName=C,ResName=UNK,ResNum=0)	-2.35780000	0.32104600	1.46100100
N(PDBName=N,ResName=UNK,ResNum=0)	-1.26339900	1.12124300	1.17372600
N(PDBName=N,ResName=UNK,ResNum=0)	-2.77333200	1.54756400	-0.35354500
C(PDBName=C,ResName=UNK,ResNum=0)	-0.03476100	1.14536500	1.94877400
C(PDBName=C,ResName=UNK,ResNum=0)	-3.45427800	2.09611300	-1.51080700
C(PDBName=C,ResName=UNK,ResNum=0)	-1.51939600	1.84955200	0.06139500
S(PDBName=S,ResName=UNK,ResNum=0)	-0.45468500	2.95480300	-0.68898100
S(PDBName=S,ResName=UNK,ResNum=0)	3.25473600	-0.82835800	-0.35110900
O(PDBName=O,ResName=UNK,ResNum=0)	2.21580900	-1.03431600	0.64596400
O(PDBName=O,ResName=UNK,ResNum=0)	2.90308200	0.20699700	-1.38159600
C(PDBName=C,ResName=UNK,ResNum=0)	1.45806500	1.41230600	-1.03796700
C(PDBName=C,ResName=UNK,ResNum=0)	-1.65299700	-0.79753900	-2.27333900
C(PDBName=C,ResName=UNK,ResNum=0)	-2.79280900	-1.58731500	-2.31861300
C(PDBName=C,ResName=UNK,ResNum=0)	-2.95480500	-2.57150900	-1.35119100
C(PDBName=C,ResName=UNK,ResNum=0)	-1.97016500	-2.72724800	-0.38700800
C(PDBName=C,ResName=UNK,ResNum=0)	-0.86343500	-1.88801100	-0.42661200
N(PDBName=N,ResName=UNK,ResNum=0)	-0.70125300	-0.93674700	-1.34761400
C(PDBName=C,ResName=UNK,ResNum=0)	-2.57770200	-0.61941900	2.45892800
C(PDBName=C,ResName=UNK,ResNum=0)	-4.55640400	-0.03548500	0.49039800
C(PDBName=C,ResName=UNK,ResNum=0)	-4.77937600	-0.97468800	1.48598600
C(PDBName=C,ResName=UNK,ResNum=0)	-3.80664500	-1.26168300	2.45273400
O(PDBName=O,ResName=UNK,ResNum=0)	3.86719100	-2.00249000	-0.94075700
C(PDBName=C,ResName=UNK,ResNum=0)	4.61462600	0.02190500	0.58348300
H(PDBName=H,ResName=UNK,ResNum=0)	0.42823800	2.12427800	1.83758200
H(PDBName=H,ResName=UNK,ResNum=0)	-0.28121200	0.98853100	2.99788800
H(PDBName=H,ResName=UNK,ResNum=0)	0.65164100	0.36874200	1.60644800
H(PDBName=H,ResName=UNK,ResNum=0)	-4.21001000	2.81768100	-1.19727800
H(PDBName=H,ResName=UNK,ResNum=0)	-2.72115900	2.58688600	-2.14651800
H(PDBName=H,ResName=UNK,ResNum=0)	-3.92676300	1.28163900	-2.06047600
H(PDBName=H,ResName=UNK,ResNum=0)	1.88839600	1.79369800	-0.12823200
H(PDBName=H,ResName=UNK,ResNum=0)	1.66189200	1.93697500	-1.95778200
H(PDBName=H,ResName=UNK,ResNum=0)	0.73302400	0.60618500	-1.03607800
H(PDBName=H,ResName=UNK,ResNum=0)	-3.83611100	-3.20213400	-1.34806600
H(PDBName=H,ResName=UNK,ResNum=0)	-1.49591100	-0.01215300	-3.00744900

H(PDBName=H,ResName=UNK,ResNum=0)	-3.53554900	-1.42943100	-3.09085700
H(PDBName=H,ResName=UNK,ResNum=0)	-2.05685900	-3.47546300	0.39049000
H(PDBName=H,ResName=UNK,ResNum=0)	-0.06899700	-1.96914300	0.30926300
H(PDBName=H,ResName=UNK,ResNum=0)	-1.82382800	-0.85205700	3.19983300
H(PDBName=H,ResName=UNK,ResNum=0)	-4.01765900	-2.00557000	3.21123200
H(PDBName=H,ResName=UNK,ResNum=0)	-5.72672000	-1.49898700	1.51548700
H(PDBName=H,ResName=UNK,ResNum=0)	-5.30566300	0.18426900	-0.25924600
F	5.04988600	-0.75819300	1.56686400
F	5.63113700	0.30540000	-0.22167400
F	4.15714800	1.15699400	1.11110200

TS-benzimidazolium-CF_3SO_3-py(2Step)-v3--

opt=(calcfc,ts,noeigen) freq=noraman wb97xd/6-311g(d,p) scrf=(iefpcm

,solvent=dmso) geom=connectivity temperature=363

Zero-point correction= 0.337630 (Hartree/Particle)

Thermal correction to Energy= 0.375571

Thermal correction to Enthalpy= 0.376721

Thermal correction to Gibbs Free Energy= 0.256285

Sum of electronic and zero-point Energies= -2105.927593

Sum of electronic and thermal Energies= -2105.889651

Sum of electronic and thermal Enthalpies= -2105.888502

Sum of electronic and thermal Free Energies= -2106.008938

Title Card Required

0 1

C(PDBName=C1,ResName=UNK,ResNum=900)	2.68958700	1.00061900	-1.11518300
C(PDBName=C2,ResName=UNK,ResNum=900)	3.20462100	0.38331000	0.02107000
N(PDBName=N1,ResName=UNK,ResNum=900)	2.54961800	0.93562900	1.10824800
N(PDBName=N2,ResName=UNK,ResNum=900)	1.75479700	1.92280000	-0.67577100
C(PDBName=C3,ResName=UNK,ResNum=900)	2.76224600	0.51156800	2.48087600
C(PDBName=C4,ResName=UNK,ResNum=900)	0.94911500	2.75572900	-1.54806800
C(PDBName=C5,ResName=UNK,ResNum=900)	1.66634600	1.85835700	0.67084100

S(PDBName=S1,ResName=UNK,ResNum=900)	0.62619700	2.79948000	1.65877100
S(PDBName=S2,ResName=UNK,ResNum=900)	-0.05312900	-1.53870100	0.71005000
C(PDBName=C6,ResName=UNK,ResNum=900)	-0.91002600	-2.97375000	-0.09950200
O(PDBName=O1,ResName=UNK,ResNum=900)	1.23428000	-2.09592200	1.11936300
O(PDBName=O2,ResName=UNK,ResNum=900)	0.00626300	-0.54676100	-0.36780400
F(PDBName=F1,ResName=UNK,ResNum=900)	-1.06794500	-3.97835900	0.76296500
F(PDBName=F2,ResName=UNK,ResNum=900)	-2.11638400	-2.61602300	-0.54600100
F(PDBName=F3,ResName=UNK,ResNum=900)	-0.20025800	-3.42038700	-1.13583600
C(PDBName=C7,ResName=UNK,ResNum=900)	-1.40365400	2.13329700	0.77836300
C(PDBName=C8,ResName=UNK,ResNum=900)	-3.55663800	0.20465900	0.28099200
C(PDBName=C9,ResName=UNK,ResNum=900)	-4.72102900	-0.35802600	-0.21868500
C(PDBName=C10,ResName=UNK,ResNum=900)	-5.55336700	0.42323600	-1.00893500
C(PDBName=C11,ResName=UNK,ResNum=900)	-5.19474800	1.73916500	-1.27353700
C(PDBName=C12,ResName=UNK,ResNum=900)	-4.01201700	2.22374000	-0.73759100
N(PDBName=N3,ResName=UNK,ResNum=900)	-3.21872700	1.46810600	0.02081600
C(PDBName=C13,ResName=UNK,ResNum=900)	4.18313000	-0.59819500	-0.05953300
C(PDBName=C14,ResName=UNK,ResNum=900)	3.11932600	0.65575000	-2.39049100
C(PDBName=C15,ResName=UNK,ResNum=900)	4.09487300	-0.32599600	-2.47578600
C(PDBName=C16,ResName=UNK,ResNum=900)	4.61890800	-0.94154500	-1.32989800
O(PDBName=O3,ResName=UNK,ResNum=900)	-0.94685700	-1.17928300	1.81477500
H(PDBName=H1,ResName=UNK,ResNum=900)	2.57956200	-0.56143800	2.54222600
H(PDBName=H2,ResName=UNK,ResNum=900)	3.78371600	0.74439800	2.78416800
H(PDBName=H3,ResName=UNK,ResNum=900)	2.06104100	1.04175600	3.12006000
H(PDBName=H4,ResName=UNK,ResNum=900)	1.56384500	3.09310600	-2.38136500
H(PDBName=H5,ResName=UNK,ResNum=900)	0.09531800	2.19033600	-1.92495800
H(PDBName=H6,ResName=UNK,ResNum=900)	0.60312100	3.62191200	-0.98786900
H(PDBName=H7,ResName=UNK,ResNum=900)	-1.81401600	2.04333400	1.77001600
H(PDBName=H8,ResName=UNK,ResNum=900)	-0.95153100	1.27030400	0.31290500
H(PDBName=H9,ResName=UNK,ResNum=900)	-6.46902100	0.01194100	-1.41652100
H(PDBName=H10,ResName=UNK,ResNum=900)	-2.86348400	-0.35789300	0.89918300
H(PDBName=H11,ResName=UNK,ResNum=900)	-4.96000200	-1.38919200	0.00611400
H(PDBName=H12,ResName=UNK,ResNum=900)	-5.81499400	2.38074800	-1.88563200
H(PDBName=H13,ResName=UNK,ResNum=900)	-3.68600500	3.24277400	-0.91740000
H(PDBName=H14,ResName=UNK,ResNum=900)	4.57754000	-1.07918900	0.82600800

H(PDBName=H15,ResName=UNK,ResNum=900)	5.37831600	-1.70604800	-1.44033900
H(PDBName=H16,ResName=UNK,ResNum=900)	4.45899100	-0.62639300	-3.45081500
H(PDBName=H17,ResName=UNK,ResNum=900)	2.70998500	1.12303100	-3.27685000
H(PDBName=H18,ResName=UNK,ResNum=900)	-1.52953700	3.05543000	0.23645600

TS-benzimidazolium-CF_3SO_3-py-v4--

\# opt=(calcfc,ts,noeigen) freq=noraman wb97xd/6-311g(d,p) scrf=(iefpcm

,solvent=dmso) geom=connectivity temperature=363

Zero-point correction= 0.246145 (Hartree/Particle)

Thermal correction to Energy= 0.275772

Thermal correction to Enthalpy= 0.276922

Thermal correction to Gibbs Free Energy= 0.176764

Sum of electronic and zero-point Energies= -1857.730855

Sum of electronic and thermal Energies= -1857.701227

Sum of electronic and thermal Enthalpies= -1857.700078

Sum of electronic and thermal Free Energies= -1857.800236

Title Card Required

0 1

C(PDBName=C,ResName=UNK,ResNum=0)	3.57714300	-0.15426300	0.25848000
C(PDBName=C,ResName=UNK,ResNum=0)	2.66772100	-0.88379300	-0.50380900
N(PDBName=N,ResName=UNK,ResNum=0)	1.70301900	0.01298300	-0.93795800
N(PDBName=N,ResName=UNK,ResNum=0)	3.12561000	1.15577400	0.27423800
C(PDBName=C,ResName=UNK,ResNum=0)	0.58696700	-0.34045600	-1.80121800
C(PDBName=C,ResName=UNK,ResNum=0)	3.79071500	2.23735000	0.97870900
C(PDBName=C,ResName=UNK,ResNum=0)	1.98749400	1.24683500	-0.45359000
S(PDBName=S,ResName=UNK,ResNum=0)	1.05861000	2.66165100	-0.69483100
S(PDBName=S,ResName=UNK,ResNum=0)	-2.86700400	-0.39746100	1.01407800
O(PDBName=O,ResName=UNK,ResNum=0)	-1.69133000	-1.18326800	0.67213400
O(PDBName=O,ResName=UNK,ResNum=0)	-2.56642600	1.06896400	1.17332800
C(PDBName=C,ResName=UNK,ResNum=0)	-0.99739900	1.76487900	0.34289700
C(PDBName=C,ResName=UNK,ResNum=0)	2.82028000	-2.24859200	-0.71222000

C(PDBName=C,ResName=UNK,ResNum=0)	4.68640100	-0.75176600	0.84317700
C(PDBName=C,ResName=UNK,ResNum=0)	4.84307100	-2.11415700	0.63541600
C(PDBName=C,ResName=UNK,ResNum=0)	3.92621900	-2.84922300	-0.12852500
O(PDBName=O,ResName=UNK,ResNum=0)	-3.73594500	-0.89179800	2.06169500
C(PDBName=C,ResName=UNK,ResNum=0)	-3.90050000	-0.41573000	-0.52830100
H(PDBName=H,ResName=UNK,ResNum=0)	0.21166100	0.56532800	-2.27249400
H(PDBName=H,ResName=UNK,ResNum=0)	0.94345900	-1.02364500	-2.57134000
H(PDBName=H,ResName=UNK,ResNum=0)	-0.20598200	-0.81383600	-1.21985700
H(PDBName=H,ResName=UNK,ResNum=0)	3.15768500	3.11986900	0.93481300
H(PDBName=H,ResName=UNK,ResNum=0)	3.94497100	1.94923900	2.01890000
H(PDBName=H,ResName=UNK,ResNum=0)	4.75117700	2.45014300	0.50789000
H(PDBName=H,ResName=UNK,ResNum=0)	-1.45308800	1.80208500	-0.63058800
H(PDBName=H,ResName=UNK,ResNum=0)	-1.02492000	2.63429300	0.97740300
H(PDBName=H,ResName=UNK,ResNum=0)	-0.40680000	0.91358100	0.63543200
H(PDBName=H,ResName=UNK,ResNum=0)	2.11081200	-2.81865300	-1.29812400
H(PDBName=H,ResName=UNK,ResNum=0)	4.08377100	-3.91186100	-0.26636700
H(PDBName=H,ResName=UNK,ResNum=0)	5.69361400	-2.62097100	1.07449200
H(PDBName=H,ResName=UNK,ResNum=0)	5.39376500	-0.18276800	1.43270200
F	-4.21206400	-1.66544100	-0.84981400
F	-5.01848800	0.27536500	-0.34923500
F	-3.21892300	0.12687100	-1.53741000

TS-CF_3SO_3-py--

opt=(calcfc,ts,noeigen) freq=noraman wb97xd/6-311g(d,p) scrf=(iefpcm
,solvent=dmso) geom=connectivity temperature=363

Zero-point correction= 0.157393 (Hartree/Particle)

Thermal correction to Energy= 0.177970

Thermal correction to Enthalpy= 0.179120

Thermal correction to Gibbs Free Energy= 0.096532

Sum of electronic and zero-point Energies= -1249.408401

Sum of electronic and thermal Energies= -1249.387823

Sum of electronic and thermal Enthalpies= -1249.386674

Sum of electronic and thermal Free Energies= -1249.469261

Title Card Required

0 1

S(PDBName=S,ResName=UNK,ResNum=0) 2.19240500 -0.84886400 -0.12087200

O(PDBName=O,ResName=UNK,ResNum=0) 1.66338200 -1.38578400 1.11956100

O(PDBName=O,ResName=UNK,ResNum=0) 1.12767300 -0.64954600 -1.17446900

C(PDBName=C,ResName=UNK,ResNum=0) -0.62069700 -0.39117300 -0.69843700

C(PDBName=C,ResName=UNK,ResNum=0) -3.38372400 -1.06774500 0.34429700

C(PDBName=C,ResName=UNK,ResNum=0) -4.73088500 -0.93386200 0.64207800

C(PDBName=C,ResName=UNK,ResNum=0) -5.36609200 0.25933700 0.32391600

C(PDBName=C,ResName=UNK,ResNum=0) -4.63313300 1.27239700 -0.28072800

C(PDBName=C,ResName=UNK,ResNum=0) -3.28990900 1.05243600 -0.54272200

N(PDBName=N,ResName=UNK,ResNum=0) -2.68154300 -0.09344200 -0.23491700

O(PDBName=O,ResName=UNK,ResNum=0) 3.38406100 -1.44529200 -0.68434700

C(PDBName=C,ResName=UNK,ResNum=0) 2.66037900 0.90185100 0.28361600

H(PDBName=H,ResName=UNK,ResNum=0) -0.51904600 0.67733200 -0.61326900

H(PDBName=H,ResName=UNK,ResNum=0) -0.96202500 -0.82011200 -1.62413500

H(PDBName=H,ResName=UNK,ResNum=0) -0.58052200 -1.00006300 0.19022300

H(PDBName=H,ResName=UNK,ResNum=0) -6.41805600 0.39808500 0.54196200

H(PDBName=H,ResName=UNK,ResNum=0) -2.84629000 -1.98218900 0.57286600

H(PDBName=H,ResName=UNK,ResNum=0) -5.26382400 -1.74956300 1.11278300

H(PDBName=H,ResName=UNK,ResNum=0) -5.08901000 2.21738500 -0.54545800

H(PDBName=H,ResName=UNK,ResNum=0) -2.67758500 1.81387200 -1.01445100

F 3.58357700 0.91515300 1.23716400

F 3.14085500 1.51348200 -0.79098000

F 1.59024800 1.56766600 0.71379000

---

34. benzimidazolium

----

opt=calcfc freq=noraman wb97xd/6-311g(d,p) scrf=(iefpcm,solvent=dmso

) geom=connectivity temperature=363

Zero-point correction= 0.177944 (Hartree/Particle)

Thermal correction to Energy= 0.193512

Thermal correction to Enthalpy= 0.194661

Thermal correction to Gibbs Free Energy= 0.129130

Sum of electronic and zero-point Energies= -856.497696

Sum of electronic and thermal Energies= -856.482128

Sum of electronic and thermal Enthalpies= -856.480978

Sum of electronic and thermal Free Energies= -856.546509

Title Card Required

0 1

C(PDBName=C1,ResName=UNK,ResNum=900)	0.78067300	0.69707400	0.00004800
C(PDBName=C2,ResName=UNK,ResNum=900)	0.78067000	-0.69709300	-0.00002000
N(PDBName=N1,ResName=UNK,ResNum=900)	-0.54623800	-1.09571200	0.00275200
N(PDBName=N2,ResName=UNK,ResNum=900)	-0.54622600	1.09570300	-0.00265300
C(PDBName=C3,ResName=UNK,ResNum=900)	-0.97409000	-2.48024400	0.00080400
C(PDBName=C4,ResName=UNK,ResNum=900)	-0.97404700	2.48024300	-0.00071500
C(PDBName=C5,ResName=UNK,ResNum=900)	-1.35815600	0.00000100	0.00001500
S(PDBName=S1,ResName=UNK,ResNum=900)	-3.04917700	0.00001900	-0.00007100
C(PDBName=C14,ResName=UNK,ResNum=900)	1.96151700	-1.42517900	-0.00239100
C(PDBName=C15,ResName=UNK,ResNum=900)	1.96151300	1.42517000	0.00236000
C(PDBName=C16,ResName=UNK,ResNum=900)	3.14738800	0.69947800	0.00143600
C(PDBName=C17,ResName=UNK,ResNum=900)	3.14738900	-0.69948100	-0.00150000
H(PDBName=H1,ResName=UNK,ResNum=900)	-0.67345600	-2.96346500	-0.93052100
H(PDBName=H2,ResName=UNK,ResNum=900)	-0.52422300	-3.00275000	0.84635900
H(PDBName=H3,ResName=UNK,ResNum=900)	-2.05721400	-2.50553700	0.09160900
H(PDBName=H4,ResName=UNK,ResNum=900)	-0.67168200	2.96387600	0.92982900
H(PDBName=H5,ResName=UNK,ResNum=900)	-2.05734000	2.50548400	-0.08953000
H(PDBName=H6,ResName=UNK,ResNum=900)	-0.52570600	3.00235200	-0.84733100
H(PDBName=H15,ResName=UNK,ResNum=900)	1.96129600	-2.50798100	-0.00578900
H(PDBName=H16,ResName=UNK,ResNum=900)	4.09199800	-1.22981000	-0.00320900
H(PDBName=H17,ResName=UNK,ResNum=900)	4.09199700	1.22980800	0.00309900
H(PDBName=H18,ResName=UNK,ResNum=900)	1.96127600	2.50797200	0.00571100

35. benzimidazole

----

opt=calcfc freq=noraman wb97xd/6-311g(d,p) scrf=(iefpcm,solvent=dmso

) geom=connectivity temperature=363

Zero-point correction= 0.218283 (Hartree/Particle)

Thermal correction to Energy= 0.236417

Thermal correction to Enthalpy= 0.237567

Thermal correction to Gibbs Free Energy= 0.167017

Sum of electronic and zero-point Energies= -896.191669

Sum of electronic and thermal Energies= -896.173534

Sum of electronic and thermal Enthalpies= -896.172385

Sum of electronic and thermal Free Energies= -896.242934

Title Card Required

1 1

C(PDBName=C1,ResName=UNK,ResNum=900)	-1.13666600	0.68401200	-0.01560700
C(PDBName=C3,ResName=UNK,ResNum=900)	-2.35442800	1.34565300	0.12055400
C(PDBName=C4,ResName=UNK,ResNum=900)	-2.18842800	-1.51016800	0.04234500
C(PDBName=C5,ResName=UNK,ResNum=900)	-3.39765800	-0.85520300	0.17851800
C(PDBName=C6,ResName=UNK,ResNum=900)	-3.47941500	0.54863300	0.21677200
C(PDBName=C7,ResName=UNK,ResNum=900)	0.80629800	-2.37644800	-0.33211500
C(PDBName=C8,ResName=UNK,ResNum=900)	0.51064000	2.57740600	-0.11044500
C(PDBName=C9,ResName=UNK,ResNum=900)	0.99099100	0.11790700	-0.24285800
C(PDBName=C10,ResName=UNK,ResNum=900)	-1.05593000	-0.70577600	-0.05750600
N(PDBName=N1,ResName=UNK,ResNum=900)	0.15819100	1.16304300	-0.12726700
N(PDBName=N2,ResName=UNK,ResNum=900)	0.28301200	-1.02210500	-0.21431900
S(PDBName=S2,ResName=UNK,ResNum=900)	2.72352900	0.23482800	-0.45269100
C(PDBName=C11,ResName=UNK,ResNum=900)	3.27410600	-0.38075500	1.17716200
H(PDBName=H1,ResName=UNK,ResNum=900)	-4.44986800	1.01635500	0.32573700
H(PDBName=H2,ResName=UNK,ResNum=900)	-2.12570900	-2.58996800	0.01593700
H(PDBName=H3,ResName=UNK,ResNum=900)	-4.30767200	-1.43652500	0.25876600
H(PDBName=H4,ResName=UNK,ResNum=900)	-2.41850100	2.42542300	0.14945500

H(PDBName=H5,ResName=UNK,ResNum=900)	0.84465800	-2.84682400	0.65029000
H(PDBName=H6,ResName=UNK,ResNum=900)	0.15316500	-2.94614400	-0.99061200
H(PDBName=H7,ResName=UNK,ResNum=900)	1.80131300	-2.33178800	-0.76857500
H(PDBName=H8,ResName=UNK,ResNum=900)	0.08432600	3.03505000	0.78115700
H(PDBName=H9,ResName=UNK,ResNum=900)	1.59271600	2.67044300	-0.08849000
H(PDBName=H10,ResName=UNK,ResNum=900)	0.11566900	3.05781400	-1.00482600
H(PDBName=H11,ResName=UNK,ResNum=900)	4.35771500	-0.27096200	1.16486800
H(PDBName=H12,ResName=UNK,ResNum=900)	2.85189700	0.23176100	1.97015700
H(PDBName=H13,ResName=UNK,ResNum=900)	3.01834900	-1.43002000	1.30937900

CF_3SO_3--

opt=calcfc freq=noraman wb97xd/6-311g(d,p) scrf=(iefpcm,solvent=dmso

) geom=connectivity temperature=363

Zero-point correction= 0.027735 (Hartree/Particle)

Thermal correction to Energy= 0.037412

Thermal correction to Enthalpy= 0.038561

Thermal correction to Gibbs Free Energy= -0.013681

Sum of electronic and zero-point Energies= -961.578763

Sum of electronic and thermal Energies= -961.569086

Sum of electronic and thermal Enthalpies= -961.567937

Sum of electronic and thermal Free Energies= -961.620180

Title Card Required

-1 1

S(PDBName=S,ResName=UNK,ResNum=0)	-0.90525900	0.00000500	0.00000000
O(PDBName=O,ResName=UNK,ResNum=0)	-1.23930500	0.71390700	-1.23296100
O(PDBName=O,ResName=UNK,ResNum=0)	-1.23890100	-1.42488500	-0.00177400
O(PDBName=O,ResName=UNK,ResNum=0)	-1.23929500	0.71083000	1.23473900
C(PDBName=C,ResName=UNK,ResNum=0)	0.94991400	0.00000800	-0.00000200
F	1.42693500	1.24738200	0.00151300
F	1.42679000	-0.62494300	1.07935000
F	1.42679300	-0.62232200	-1.08086500

CH_3SO_3-py--

\# opt=calcfc freq=noraman wb97xd/6-311g(d,p) scrf=(iefpcm,solvent=dmso

) geom=connectivity temperature=363

Zero-point correction= 0.050917 (Hartree/Particle)

Thermal correction to Energy= 0.057801

Thermal correction to Enthalpy= 0.058950

Thermal correction to Gibbs Free Energy= 0.015071

Sum of electronic and zero-point Energies= -663.833203

Sum of electronic and thermal Energies= -663.826320

Sum of electronic and thermal Enthalpies= -663.825170

Sum of electronic and thermal Free Energies= -663.869049

Title Card Required

-1 1

S(PDBName=S,ResName=UNK,ResNum=0)	-0.15577700	0.00051800	0.00000000
O(PDBName=O,ResName=UNK,ResNum=0)	-0.55365900	-0.71088200	1.23189100
O(PDBName=O,ResName=UNK,ResNum=0)	-0.55365900	1.42289900	0.00000000
O(PDBName=O,ResName=UNK,ResNum=0)	-0.55365900	-0.71088200	-1.23189100
C	1.63614300	-0.00158700	0.00000000
H	1.98883400	0.51291100	-0.89330100
H	1.98883400	0.51291100	0.89330100
H	1.98571900	-1.03366200	0.00000000

36. TFSI

\# opt=calcfc freq=noraman wb97xd/6-311g(d,p) scrf=(iefpcm,solvent=dmso

) geom=connectivity temperature=363

Zero-point correction= 0.054554 (Hartree/Particle)

Thermal correction to Energy= 0.074004

Thermal correction to Enthalpy= 0.075154

Thermal correction to Gibbs Free Energy= -0.001478

Sum of electronic and zero-point Energies= -1827.261578

Sum of electronic and thermal Energies= -1827.242128

Sum of electronic and thermal Enthalpies= -1827.240979

Sum of electronic and thermal Free Energies= -1827.317610

Title Card Required

-1 1

S(PDBName=S2,ResName=UNK,ResNum=900)	-1.14363400	-0.84361000	0.08877700
S(PDBName=S3,ResName=UNK,ResNum=900)	1.14378000	0.84380300	0.08877500
C(PDBName=C6,ResName=UNK,ResNum=900)	2.54101400	-0.38235000	-0.03926700
C(PDBName=C7,ResName=UNK,ResNum=900)	-2.54116100	0.38220000	-0.03926600
O(PDBName=O1,ResName=UNK,ResNum=900)	-1.62994100	-1.84116500	1.02132300
O(PDBName=O2,ResName=UNK,ResNum=900)	-0.87385400	-1.24269200	-1.28005100
O(PDBName=O3,ResName=UNK,ResNum=900)	1.63028000	1.84125600	1.02132500
O(PDBName=O4,ResName=UNK,ResNum=900)	0.87412600	1.24293800	-1.28006200
F(PDBName=F1,ResName=UNK,ResNum=900)	2.17197800	-1.43945100	-0.75122700
F(PDBName=F2,ResName=UNK,ResNum=900)	3.57610300	0.19901000	-0.63687700
F(PDBName=F3,ResName=UNK,ResNum=900)	2.91301900	-0.78519500	1.16864500
F(PDBName=F4,ResName=UNK,ResNum=900)	-2.91303800	0.78521500	1.16862700
F(PDBName=F5,ResName=UNK,ResNum=900)	-2.17251800	1.43923300	-0.75152600
F(PDBName=F6,ResName=UNK,ResNum=900)	-3.57622200	-0.19954500	-0.63655400
N(PDBName=N3,ResName=UNK,ResNum=900)	-0.00003400	0.00024300	0.81718400

37. py

----

opt=calcfc freq=noraman wb97xd/6-311g(d,p) scrf=(iefpcm,solvent=dmso

) geom=connectivity temperature=363

Zero-point correction= 0.089448 (Hartree/Particle)

Thermal correction to Energy= 0.095560

Thermal correction to Enthalpy= 0.096710

Thermal correction to Gibbs Free Energy= 0.054780

Sum of electronic and zero-point Energies= -248.168181

Sum of electronic and thermal Energies= -248.162068

Sum of electronic and thermal Enthalpies= -248.160918

Sum of electronic and thermal Free Energies= -248.202849

Title Card Required

0 1

C(PDBName=C9,ResName=UNK,ResNum=900)	-1.13936000	-0.71861100	-0.00001600
C(PDBName=C10,ResName=UNK,ResNum=900)	-1.19356300	0.67000000	-0.00001300
C(PDBName=C11,ResName=UNK,ResNum=900)	0.00024900	1.37927200	0.00000800
C(PDBName=C12,ResName=UNK,ResNum=900)	1.19381700	0.66954800	-0.00003400
C(PDBName=C13,ResName=UNK,ResNum=900)	1.13908600	-0.71901000	-0.00000600
N(PDBName=N4,ResName=UNK,ResNum=900)	-0.00025300	-1.41471400	0.00001800
H(PDBName=H10,ResName=UNK,ResNum=900)	0.00051800	2.46319500	0.00008200
H(PDBName=H11,ResName=UNK,ResNum=900)	-2.05696200	-1.30015500	0.00003900
H(PDBName=H12,ResName=UNK,ResNum=900)	-2.15042200	1.17722400	0.00001900
H(PDBName=H13,ResName=UNK,ResNum=900)	2.15082100	1.17647900	0.00006600
H(PDBName=H14,ResName=UNK,ResNum=900)	2.05645100	-1.30093900	0.00003400

References

[1] R. Guterman, H. Miao, M. Antonietti, *J. Org. Chem.* **2018**, *83*, 684–689.

[2] H. Lin, **2012**, *39*, 161–170.

[3] A. D. Becke, *J. Chem. Phys.* **1993**, *98*, 5648–5652.

[4] B. Herce-Sesa, J. A. López-López, J. J. Pinto, C. Moreno, *Chem. Eng. J.* **2017**, *308*, 649–655.

[5] J. Tomasi, B. Mennucci, E. Cance, **2018**, *464*, 211–226.

[6] M. Moosavi, F. Khashei, E. Sedghamiz, *Phys. Chem. Chem. Phys.* **2017**, *20*, 435–448.

[7] M. O. Sinnokrot, C. D. Sherrill, *J. Phys. Chem. A* **2006**, *110*, 10656–10668.

[8] M. O. Sinnokrot, C. D. Sherrill, *J. Am. Chem. Soc.* **2004**, *126*, 7690–7697.

[9] J. Da Chai, M. Head-Gordon, *Phys. Chem. Chem. Phys.* **2008**, *10*, 6615–6620.

Author Contributions

The project was designed by RG, SMT and JFT. Experimental data was collected by VAC, computational calculations by SMT, DOSY NMR data were collected by AS. Funding acquired and project administration by JFT and RG. VAC and SMT wrote the first draft, edited by JFT, RG and SMT. SMT and VAC contributed equally to the publication as lead authors, JFT and RG contributed equally as senior authors.
